# Body weight in autism spectrum disorder: A systematic review and meta‐analysis

**DOI:** 10.1002/jcv2.70151

**Published:** 2026-08-04

**Authors:** Victoria Nieuwenhuys‐Ruiz, Leyre Gambra, Samuele Cortese, Pablo Lizoain, Diana Rodriguez‐Romero, Ursula Paiva, Carmen Gándara, Sara Magallón, Gonzalo Arrondo

**Affiliations:** ^1^ Mind‐Brain Group Institute for Culture and Society (ICS) University of Navarra Pamplona Spain; ^2^ Laboratoire de Psychopathologie et Processus de Santé Université Paris Cité Boulogne‐Billancourt France; ^3^ Laboratoire de Cognitions Humaine et Artificielle (CHArt–UR 4004) CY Cergy Paris Université Cergy‐Pontoise France; ^4^ Faculty of Education and Psychology University of Navarra Pamplona Spain; ^5^ Centre for Innovation in Mental Health School of Psychology Faculty of Environmental and Life Sciences University of Southampton Southampton UK; ^6^ Clinical and Experimental Sciences (CNS and Psychiatry) Faculty of Medicine University of Southampton Southampton UK; ^7^ Solent NHS Trust Southampton UK; ^8^ DiMePRe‐J‐Department of Precision and Regenerative Medicine‐Jonic Area University of Bari “Aldo Moro” Bari Italy; ^9^ Departamento de Psicología Universidad Villanueva Madrid España

**Keywords:** autism, meta‐analysis, obesity, overweight, systematic review, underweight

## Abstract

**Background:**

Evidence on the relationship of autism spectrum disorder (ASD) to overweight/obesity and underweight is mixed. Moreover, it is unclear how this relationship changes with age.

**Methods:**

We carried out a systematic review (SR) with meta‐analysis on the association between ASD and obesity, overweight and underweight across the life span. The study was based on a pre‐registered protocol (PROSPERO: CRD42024524084). Web of Science and PubMed were searched until the 8th of April 2025. We assessed the quality of the included studies with the Newcastle‐Ottawa scales (NOS).

**Results:**

A total of 9075 records were screened, of which 64 studies reporting data on obesity, overweight and/or underweight on ASD and controls general participants were included in the SR and 62 meta‐analyzed. We found a significant association between ASD and obesity (OR = 1.93; CI = 1.70–2.19; *i* ^2^ = 99.02%), ASD and overweight (OR = 1.73; CI = 1.43–2.09; *i* ^2^ = 95.44%) but, at the group level, not ASD and underweight (OR = 1.22; CI = 0.91–1.64; *i* ^2^ = 78.37%). There was a statistically significant association between ASD and underweight in adolescents (OR = 1.32; CI = 1.12–1.56) and adults (OR = 2.14; CI = 1.5–3.04). The score on the Newcastle‐Ottawa Scale showed that, overall, studies had a medium risk of bias (median = 6, range 1–10).

**Conclusion:**

Individuals with ASD have an elevated risk of being obese or overweight, and, in adolescents and adults, of underweight. Our study may contribute to the development and implementation of targeted prevention and intervention strategies, aimed at enhancing the physical well‐being of individuals with ASD and their caregivers, ultimately improving their quality of life.

## Key Points

**What's known?**

- Previous evidence on the relationship between ASD and weight status (overweight, obesity, and underweight) has been inconsistent, with potential age‐related variations.

**What's new?**

- This systematic review of 64 studies, of which 62 were meta‐analyzed, provides a comprehensive examination of the associations between ASD and obesity, overweight, and underweight across the lifespan. Significant associations were found for obesity and overweight, and age‐specific associations for underweight in adolescents and adults.

**What's relevant?**

- These findings highlight the need for targeted prevention and intervention strategies to support healthy weight management in individuals with ASD. The results have implications for clinical practice, public health policy, and educational or service‐based programs, potentially contributing to improved physical well‐being and quality of life.

## INTRODUCTION

Autism Spectrum Disorder (ASD) is a neurodevelopmental condition characterized by symptoms that appear in early development. It involves persistent deficits in social communication and interaction, along with restricted, repetitive patterns of behavior, interests, or activities (American Psychiatric Association, 2022).

In addition, ASD may be associated with a range of comorbidities, including psychiatric and medical conditions (Micai et al., 2023) such as anxiety, ADHD, and depressive disorder (Lai et al., 2019), dermatitis (Tsai et al., 2020), food allergy (Li et al., 2021), diabetes (Cortese et al., 2022) and cardiometabolic diseases (Dhanasekara et al., 2023). Understanding the differential risk of these comorbidities is clinically important, as early recognition supports more timely assessment and management (Cortese et al., 2025; Micai et al., 2023).

One area of growing concern is the relationship between ASD and abnormal body weight. Both elevated and low body weight can have substantial effects on health and quality of life across the lifespan, and their prevention and management are key public health aims (Kolotkin & Andersen, 2017; Lorem et al., 2017). Individuals with ASD may be particularly vulnerable to the consequences of abnormal body weight because these difficulties interact with core features and common comorbidities of the condition (Dhaliwal et al., 2019; Kahathuduwa et al., 2019, 2022). Excess weight can exacerbate cardiometabolic problems, reduce mobility, and worsen sleep disturbances (Figorilli et al., 2025), issues that are already more prevalent in autistic populations (Sammels et al., 2022) and can further impair daily functioning and participation. Obesity may also exacerbate gastrointestinal symptoms and fatigue, contributing to increased sedentary behavior and challenging behavioral patterns (Bora et al., 2024; Eslick, 2012). Conversely, low body weight may lead to nutrient deficiencies, weakened immunity, and reduced bone and muscle development (Allen & Saunders, 2023). In children and adolescents, underweight can also negatively affect growth, energy levels, and cognitive development (Roberts et al., 2022), potentially amplifying existing developmental vulnerabilities associated with ASD. Together, these consequences highlight the clinical relevance of monitoring and addressing both overweight and underweight in individuals with ASD across the lifespan.

Multiple mechanisms related to ASD may increase vulnerability to weight alterations, including food selectivity, gastrointestinal symptoms, medication effects, and lifestyle factors (Zheng et al., 2017). With respect to selective eating, children with ASD often exhibit sensory sensitivities that lead to aversions to specific colors, smells, temperatures, and textures, predisposing them to diets with limited variety (Evans et al., 2012). Additionally, individuals with ASD tend to engage in lower levels of physical activity and present with more sedentary behaviour, further contributing to overweight and obesity risk. Regarding medication use, so called “second generation antipsychotics”, such as risperidone, which are used to reduce disruptive behavior in these children, can have a negative side effect of weight gain (Correll, 2009), whereas medications for ADHD symptoms, a highly common comorbidity, can reduce appetite (Petruzzelli et al., 2026). Moreover, there is a growing interest in the possible link between eating disorders (especially anorexia nervosa) and ASD, due to the presence of ASD traits among eating disorder patients, and abnormal eating behaviors reported in some individuals with ASD (Dell’Osso et al., 2018; Westwood et al., 2016; Westwood & Tchanturia, 2017).

Despite these plausible mechanisms, existing evidence on the direction and magnitude of the association between ASD and body‐weight status remains mixed. Some studies report higher rates of obesity or overweight in ASD (Bandini et al., 2013; Hand et al., 2020), while others highlight underweight risk in subgroups, potentially linked to feeding difficulties or comorbid eating‐disorder traits (Amin et al., 2022; Molina‐López et al., 2021). Meta‐analyses have shown an increased risk of overweight and obesity in children and adolescents with ASD (Kahathuduwa et al., 2019; Sammels et al., 2022; Zheng et al., 2017), and an umbrella review suggests such associations may be part of a broader transdiagnostic pattern across psychiatric conditions (Arrondo et al., 2022). However, prior reviews differ considerably in their inclusion criteria, populations studied, and operationalization of weight categories, making comparisons difficult. Evidence on underweight is scarce and inconclusive: the only meta‐analysis on this outcome combined ten studies and reported no significant association, without disaggregating results by age (Kahathuduwa et al., 2022).

Crucially, no previous meta‐analysis has simultaneously examined obesity, overweight, and underweight using a unified protocol and analytic strategy across the full lifespan, despite many studies reporting data across all weight categories. Age may be particularly relevant, as developmental changes in eating behavior, autonomy, activity patterns, and medication exposure might alter the direction of weight‐related risk across childhood, adolescence, and adulthood in neurodevelopmental disorders (Gambra et al., 2024).

Therefore, the present systematic review and meta‐analysis aimed to provide the most comprehensive and age‐specific synthesis to date of the association between ASD and obesity, overweight, and underweight. By applying consistent inclusion criteria and statistical methods across all weight categories and age groups, our study addressed the limitations of previous reviews and offers a clearer picture of weight‐related risks in ASD, with implications for prevention and clinical intervention.

## METHODS

We followed the 2020 PRISMA guidelines (M. J. Page et al., 2021), and pre‐registered the protocol in PROSPERO (CRD42024524084) on 8 April 2024. In the original protocol, we planned to carry out a full meta‐analysis on underweight and an umbrella review synthesizing existing meta‐analysis on obesity and overweight. However, it was finally decided to conduct meta‐analysis for all weight categories (obesity, overweight and underweight) to provide a more comprehensive synthesis of the literature.

### Search strategy

PubMed (Medline Plus) and Web of Science (Core Collection, MEDLINE, SciELO) were searched in March 2024 and again on the 8th of April 2025. The search combined keywords for ASD and for body weight, namely: (Autism OR ASD OR autistic OR Asperger OR “pervasive developmental disorder”) AND (underweight OR weight OR obesity OR overweight OR anorexia OR BMI OR “Body Mass Index”). No restrictions regarding date of publication, type of document or language were implemented. Database searches were complemented with a review of references present in previous meta‐analyses.

### Eligibility

The present systematic review with meta‐analysis was conducted following the PECO framework: Population, individuals of all ages from the general population; Exposure, diagnosis of ASD; Comparator, individuals without ASD from a non‐clinical population; Outcomes, prevalence of obesity, overweight, and underweight.

We accepted the following definitions of ASD: 1‐ a clinical diagnosis of ASD (or related labels) according to the DSM (III, III‐R, IV, IV‐TR or 5 and 5‐TR) or ICD (9, 10, or 11); 2‐ a diagnosis based on the Autism Diagnostic Observation Schedule (ADOS), the Autism Diagnostic Interview‐Revised (ADI‐R) or similar tools, 3‐ a self‐reported diagnosis (e.g.,: a positive answer to the question: “Did your doctor ever tell you that you have ASD?”); 4‐ a diagnosis of ASD recorded in medical files/registries; 5‐ a result over a pre‐specified threshold in a validated symptom scale.

Regarding weight, we followed the original definitions used by primary study authors but aimed to harmonize them into a common framework broadly consistent with WHO criteria, in which weight status is defined using age‐ and sex‐adjusted BMI percentiles or z‐scores, allowing comparability across populations and studies. WHO classifies children as obese (≥95th percentile or *Z* ≥ 2), overweight (≥85th percentile or *Z* ≥ 1 to 1.5), and underweight (≤5th percentile or *Z* ≤ −2). In the case of adults, WHO defines obesity as a BMI of 30 kg/m^2^ or higher, overweight above 25 kg/m^2^ and underweight as a BMI below 18,5 kg/m^2^ (Bray et al., 2018; Enomoto et al., 2020; Voulgarakis et al., 2017). While most studies followed this characterization, in some cases the definition used by the original authors was not stated or differed.

We included any type of primary study (e.g., not meta‐analysis, reviews, case‐reports) that reported risk measures, or the data to calculate them (namely, the number of individuals in the different weight categories for cases and controls), on the association of obesity (odds ratios, risk ratios), overweight or underweight in ASD and controls from the general population. Studies reporting Body Mass Index (BMI) as a continuous measure were excluded to ensure consistency with our focus on clinically defined weight categories and directly comparable odds ratios.

### Selection and data collection processes

Screening and full text evaluation was carried out independently by two authors (V.N., L.G., P.L., D.R., S.M. U.P, C.G, G.A) in Covidence (Covidence Systematic Review Software, 2025), which was followed by comparing decisions and consensus seeking regarding inclusion. If consensus was not reached, the senior author (G.A.) decided on a given record or study.

Data extraction was carried out in Google Sheets (V.N., L.G., P.L., D.R., S.M. U.P, C.G, G.A). The following data were extracted: publication details (year, country), design (case‐controlled, cross‐sectional or nested case‐controlled study within a cohort), and details of the overall, ASD, and non‐ASD samples (age, gender distribution, socio‐economic status and ethnicity). The operationalization of ASD diagnosis and weight categories were also registered. Finally, the number of individuals with and without ASD and with and without obesity/overweight/underweight were also extracted.

In cases where data were unclear, corresponding authors were contacted at least twice to ask for additional details.

### Risk of bias assessment

A modified version of the Newcastle‐Ottawa scale (NOS) (Wells et al., 2011) was used to evaluate the risk of bias of studies respectively (Supporting Information S1: Table S1). This tool evaluates the risk of bias in the selection and representativeness of the groups (4 items), their comparability (1 item), and the definition of outcomes (5 items in our version). Independent double utilization of the tool followed by comparison and discussion were also implemented at this stage (V.N., G.A).

### Data synthesis

Analyses were conducted in STATA 18 (StataCorp, 2023) by G.A. We pooled odds ratio (ORs) for the association between ASD and obesity, overweight or underweight with three restricted maximum likelihood (REML) random‐effects meta‐analyses. As a final analysis, we reported the association between ASD and abnormal weight, that is, of individuals being either obese, overweight or underweight.

These analyses were operationalized as follows. To enable comparability across studies, we defined each outcome using binary contrasts based on commonly used thresholds. For obesity, we compared individuals above versus below the study‐specific high‐weight cut‐off (typically around the ≥95th percentile or equivalent, such as *Z* ≥ 2). For overweight, we compared those at or above the ≥85th percentile (including individuals with obesity) versus those below this threshold, thus capturing overweight and obesity combined. For underweight, we compared individuals below the ≤5th percentile (or equivalent) versus all others. When studies used alternative but comparable definitions, we applied the closest equivalent threshold. Whenever studies reported zero cases for a given weight category, 0.5 was added to all cells of the confusion matrix to be able to calculate odds ratios in these cases.

Heterogeneity was evaluated using tau and I^2^ metrics. Funnel plots and Egger tests were utilized to detect small‐sample bias. Additionally, a leave‐one‐out (LOU) method was used to assess the impact of individual studies on the overall analysis. The main subgroup analysis corresponded to the effect across age ranges (children, adolescents or adults). Other subgroup analyses were community‐based studies vs hospital‐based studies, studies using a gold standard diagnosis for ASD, or studies with a definition of weight categories that explicitly followed those of WHO. Similarly, we carried out two sensitivity analyses: in one of them, we eliminated those studies with a weight category with no cases; in the second, we evaluated the influence of studies with very few cases per weight category by eliminating those studies that had less than five individuals in any of the reported weight categories for cases or controls. The rationale for these two analyses was that studies with a small number of cases in a given category might be led to a noisier estimation of the effects. We investigated the effect of sex (percentage of males in the ASD and overall group) and mean age of the overall group in meta‐regression analyses.

## RESULTS

### Search results

Our search yielded 28787 references. After duplicate removal, screening and full‐text evaluation, 64 studies were selected (see Supporting Information S1: Table S2 for the full list of included studies).

**FIGURE 1:**
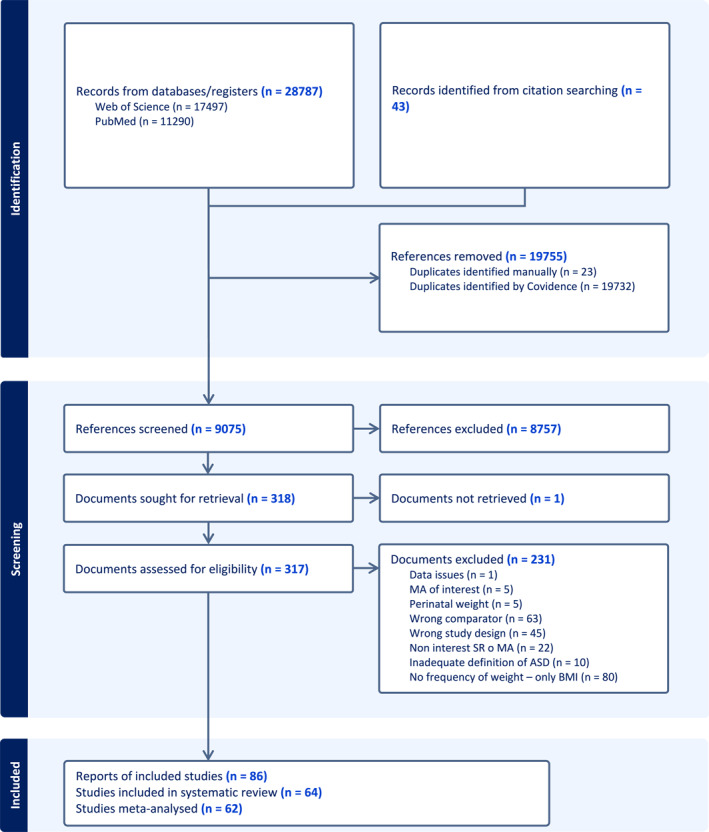
Flow diagram of the selection process following the PRISMA guidelines.

Our study selection process is depicted in Figure 1. Further details on a study that was unavailable and the reasons for exclusion of studies during the full text evaluation can be found in Supporting Information S1: Tables S3 and S4. The 64 included primary studies derived from 86 reports (Supporting Information S1: Table S5). We were unable to include a study due to data concerns that went unanswered by the study authors (Supporting Information S1: Table S6) (Hammouda et al., 2018). Two studies that explicitly excluded cases of underweight from the final sample were summarized qualitatively, but their results were not meta‐analyzed along the other studies due to the risk of bias derived from selection bias (Eliasziw et al., 2021; Must, Eliasziw, Bowling, et al., 2023).

### Study characteristics

The main study characteristics of included studies are shown in Table 1, while Supporting Information S1: Tables S7 to S9 provide additional information on the overall sample of participants, the ASD group and the non‐ASD group. The most frequent country where the studies had been conducted was the United States (*k* = 30), followed by Spain (*k* = 5) and Italy (*k* = 4). Thirty‐nine studies were case‐control, sixteen studies were cross‐sectional and nine were nested case‐controlled studies. The study samples were mainly based on community (*k* = 44) or hospital (*k* = 18) settings. To diagnose ASD, twenty‐three studies used a clinical method, 15 studies used the Autism Diagnostic Observation Schedule (ADOS) and/or the Autism Diagnostic Interview‐Revised (ADI‐R), 12 studies used self‐report, and four studies used a symptom rating scale. Obesity was most commonly defined as > pc95 (*k* = 32), whereas other studies used > Z2 (*k* = 16) or an unclear/other definition (*k* = 12). The most frequent definition of overweight was >pc85 (*k* = 30), whereas other studies used > Z1.5 (*k* = 13) or an unclear/other definition (*k* = 4). Across studies, underweight was typically defined as < pc5 (*k* = 25), although some used < Z2 (*k* = 13) or an unclear/other definition (*k* = 3). The total sample size included 3,346,170 participants (median = 440.5; range = 98‐3,141,696), of which 54,957 individuals had ASD. Most studies had a sample of children (*k* = 37), while then studies evaluated BMI in children and adolescents, five in adolescents and eight in adults. The mean age of the total sample ranged between 4.15 and 29 years (median = 8.85 years), and the median overall proportion of males was 64.53%. The score on the Newcastle‐Ottawa Scale showed that, overall, studies had a medium risk of bias (median = 6, range 1–10). The rating for each study and item is reported in Supporting Information S1: Table S10.

**TABLE 1:** Description of all the included studies (*N* = 64).

Author year	Country	Database acronym	Design	Setting	Method of diagnosis of ASD	*N*	Mean age	Age category	Gender (% M)
Ahlberg (2022)	Sweden	MBR/STPR/MGR/SNPR/SPDR	cs	Community‐based	Administrative database	3141696	21.16	Adults	51.93
Al‐Kindi (2016)	Oman		cc	Hospital‐based	Clinical	375	8.5^a^	Children	62.14
Alkhalidy (2021)	Jordan		cc	Community‐based	Clinical	103	4.29	Children	61.2
Ames (2022)	USA	KPNC	nccs	Community‐based	Administrative database	16577	29^b^	Adults	73.10
Amin (2022)	Pakistan		cc	Community‐based	Clinical	180	9.20	Children	52.7
Arija (2023)	Spain	EPINED	cs	Community‐based	ADI‐ADOS	450	8.41	Children	63.08
Bandini (2013)	USA	CHAMPS	cs	Community‐based	ADI‐ADOS	111	6.65	Children	80.39
Barnhill (2017)	USA		cc	Other (describe in notes)	ADI‐ADOS	143	5.79	Children	88.01
Bener (2014)	Qatar		cc	Hospital‐based	Clinical	508	5.64	Children	60.80
Błazewicz (2020)	Poland		cc	Hospital‐based	Clinical	287	8.09	Children	71.43
Broder‐Fingert (2014)	USA	RPDR	cs	Community‐based	Administrative database	6672	11.26	children + adol + adults	62.99
Buro (2022)	USA	NSCH 2017–2018	cs	Community‐based	Self‐reported	27157	13.81	Children + adolescents	51.14
Castro (2016)	Brazil		cc	Hospital‐based	Clinical	98	10.26	Children	100
Chen (2016)	Taiwan	NHIRD	nccs	Community‐based	Clinical	30610	15.25	adolescents + adults	80
Coppola (2024)	Italy	NAFRA	cc	Hospital‐based	Clinical	200	4.15	Children	77.10
Corbett (2021)	USA		cc	Community‐based	Clinical	241	11.55	Children	65.98
Criado (2018)	USA		nccs	Community‐based	Clinical	820	10.5^a^	Children + adolescents	84.40
Curtin (2010)	USA	NSCH 2003–2004	cs	Community‐based	Self‐reported	84923	10^a^	Children + adolescents	51.15
Davignon (2018)	USA	KPNC	nccs	Community‐based	Administrative database	24738	18.4	adolescents + adults	80.67
Eliasziw (2021)	USA	ECLS‐K	cs	Community‐based	Self‐reported	9750	6.1	Children	51.40
Esposito (2019)	Italy		cc	Other (describe in notes)	Clinical	100	4.59	Children	81.00
Esteban‐Figuerola (2021)	Spain	EPINED	cc	Community‐based	ADI‐ADOS	431	8.49	Children	61.95
Flygare Wallen (2018)	Sweden	VAL	cs	Community‐based	Administrative database	2023128	42.1^c^	children + adol + adults	49.40
Fuentes‐Albero (2024)	Spain		cc	Hospital‐based	Clinical	40	9.9	Children + adolescents	80
Garcia (2023)	USA	NSCH 2019–2020	cs	Community‐based	Self‐reported	27645736	13.49	Children	51.15
Hand (2020)	USA	SAF	nccs	Community‐based	Administrative database	51535	71.2	Adults	67.77
Healy (2017)	Ireland	GUI	cs	Community‐based	Self‐reported	141	13	Adolescents	65.24
Herndon (2009)	USA		cc	Hospital‐based	ADI‐ADOS	77	4.79	Children	87.04
Hill (2015)	USA and Canada	ATN	cc	Hospital‐based	ADI‐ADOS	13897	7.5^a^	Children + adolescents	
Hyman (2012)	USA	ATN	cc	Hospital‐based	ADI‐ADOS	926	5.37	Children	34.08
Islam (2024)	Bangladesh		cc	Community‐based	Clinical	344	7.9	Children	70.4
Kerekes (2015)	Sweden	CATSS	cs	Community‐based	Rating/symptom scale	11355	10.57	Children	51.9
Kral (2015)	USA		cc	Community‐based	Clinical	55	5.11	Children	58.36
Kummer (2016)	Brazil		cc	Hospital‐based	Clinical	88	8.44	Children	68.14
Levy (2019)	USA	SEED	cc	Community‐based	ADI‐ADOS	2466	4.92	Children	82
Liu (2016)	China		cc	Community‐based	Clinical	227	5.09	Children	91.63
Mari‐Bauset (2015)	Spain		cc	Community‐based	ADI‐ADOS	600	7.93	Children	59.78
Mathew (2022)	Australia	AAB/DISH	cc	Hospital‐based	ADI‐ADOS	367	7.68	Children	62.7
Mayes (2024)	USA		cc	Community‐based	Clinical	1652	8.34	Children + adolescents	68.61
McCoy (2016)	USA	NSCH 2011–2012	cs	Community‐based	Self‐reported	42747	13.66	Adolescents	51.80
McCoy (2020)	USA	NSCH 2016–2017	cs	Community‐based	Self‐reported	32 312	13.80	Adolescents	50.98
Mehra (2024)	USA	NSCH 2018–2019	cs	Community‐based	Self‐reported	31344	13.5^a^	Children + adolescents	52.18
Mendive (2022)	Uruguay		cc	Community‐based	Clinical	65	6.46	Children	
Mills (2007)	USA		cc	Hospital‐based	ADI‐ADOS	130	6.55	Children	100
MMetwally (2024)	Egypt		cc	Community‐based	Clinical	509	6.69	Children	
Molina‐Lopez (2021)	Spain		cc	Community‐based	Clinical	144	11.66	Children	
Must (2023a)	USA	SSC	cc	Community‐based	Administrative database	2756	10.5^a^	Children + adolescents	66.30
Must (2023b)	USA	ABCD	nccs	Community‐based	Self‐reported	1116	9.55	Children	85.50
Phillips (2014)	USA	NHIS	cs	Community‐based	Self‐reported	9619	14.5^a^	Adolescents	50
Raspini (2021)	Italy		cc	Hospital‐based	ADI‐ADOS	147	3.66	Children	71.41
Riccio (2020)	Italy		cc	Hospital‐based	ADI‐ADOS	91	4.67	Children	63.73
Rimmer (2010)	USA	YRBS data	cc	Community‐based	Self‐reported	13132	15^a^	Adolescents	
Rouphael (2024)	Lebanon		cc	Community‐based	Rating/symptom scale	172	10.4	Children + adolescents	86
Schott (2022)	USA	CMS, MAX	nccs	Community‐based	Administrative database	622468	30.47	Adults	74.1
Sedgewick (2019)	UK		cc	Community‐based	Rating/symptom scale	665	33.37	Adults	15.91
Shea (2024)	USA	CMS	cc	Community‐based	Administrative database	452143	26.3	Adults	0
Shedlock (2016)	USA	MHS	cs	Community‐based	Administrative database	292572	10^a^	Children + adolescents	79.79
Trinh (2023)	Vietnam		cc	Community‐based	Clinical	186		Children	65.59
Tyler (2011)	USA	IIDDEA	nccs	Hospital‐based	Clinical	314	29	Adults	71.63
Wei (2018)	China		nccs	Hospital‐based	Clinical	166	3.5^a^	Children	90.36
Weir (2021)	Worldwide	CARD/others	cc	Community‐based	Self‐reported	2386	41.45	Adults	34.16
Zhu (2020)	China		cc	Hospital‐based	Rating/symptom scale	1040	4.36	Children	75.10
Zimmer (2012)	USA		cc	Hospital‐based	ADI‐ADOS	44	8.15	Children	68.00
Zurita (2020)	Ecuador		cc	Community‐based	ADI‐ADOS	60	8.65	Children	93.32

*Note*: Age data typically refers to the age of measurement of BMI.

Abbreviations: AAB/DISH, Australian Autism Biobank/Dietary Intake Study in Children; ABCD, Adolescent Brain Cognition Development Study; ATN, Autism Speaks Autism Treatment Network; CARD, Cambridge Autism Research Database; CATSS, Child and Adolescent Twin Study in Sweden; cc, case‐controlled study; CHAMPS, Children's Activity and Meal Patterns Study; CMS, Centers for Medicare and Medicaid Services; cs, cross‐sectional study; ECLS‐K, Early Childhood Longitudinal Study (Kindergarten Class); EPINED, Neurodevelopmental Disorders Epidemiological Research Project; GUI, Growing Up in Ireland; IIDDEA, Individuals With Intellectual and Other Developmental Disabilities Electronic Health Record Analysis; KPNC, Kaiser Permanente Northern California; MAX, Medicaid Analytic eXtract; MBR, Medical Birth Register; MGR; Cause of Death Register and the Multi‐Generation Register; MHS, Military Health System Database; NAFRA, Nutritional status and Adverse Food Reactions in children with Autism Spectrum Disorder; nccs, nested case‐controlled study; NHIRD, Taiwan National Health Insurance Research Database; NHIS, National Health Interview Survey; NSCH, National Survey of Children's Health; RPDR, Partners HealthCare System Research Patient Database Repository; SAF, Medicare Standard Analytic File; SEED, Study to Explore Early Development; SNPR, Swedish National Patient Register; SPDR, Swedish Prescribed Drug Register; SSC, Simons Simplex Collection Version 15.3; STPR, Swedish Total Population Register; VAL, Central administrative database in Stockholm County; YRBS data, Youth Risk Behavior Survey.

^a^ Midpoint of the interval.

^b^ Obtained from: Croen et al. (2015).

^c^ Estimated as Sweden average age in 2018.

### Data synthesis

The number and prevalence of individuals in the different weight categories can be found in Supporting Information S1: Tables S11–S14.

#### Obesity

**FIGURE 2:**
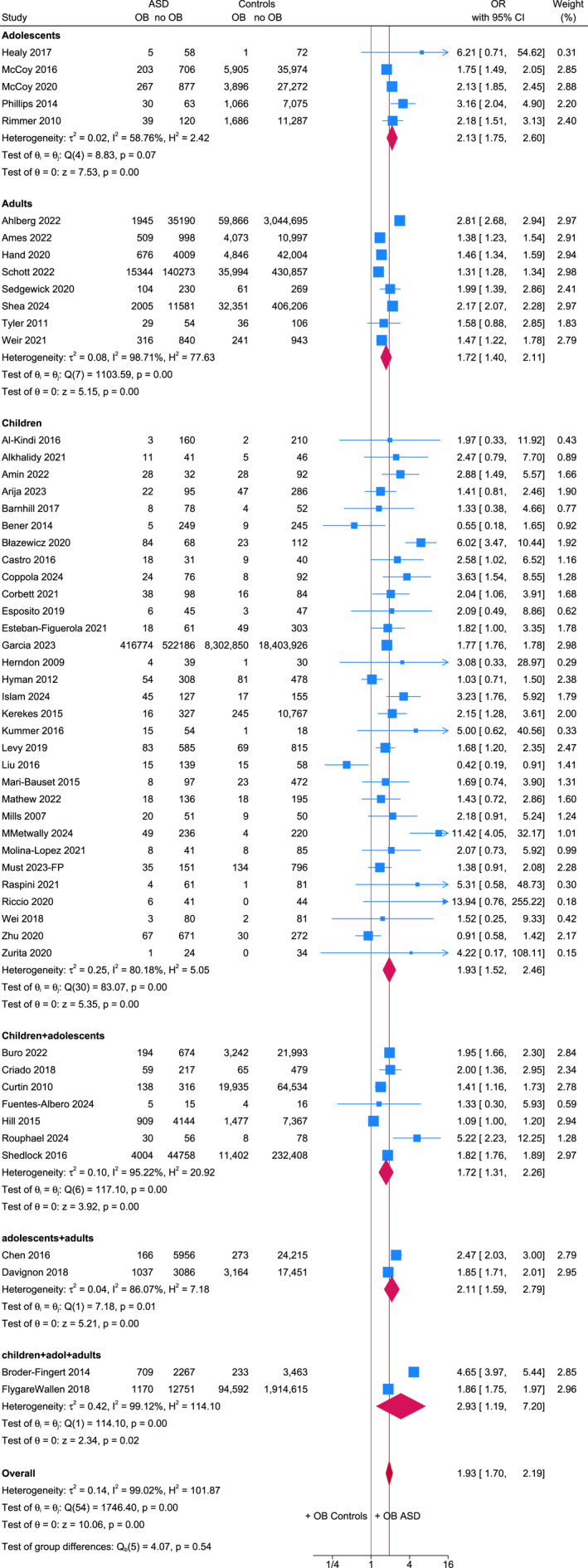
Risk of obesity. Results are presented according to age group. OB: number of individuals with obesity. No OB: number of individuals without obesity. +OB Controls: higher odds of obesity in the control group. +OB ASD: higher odds of obesity in the ASD group.

Our results show that individuals with ASD had a significant association with obesity, as seen in Figure 2. The overall OR was 1.93 (*k* = 55; Confidence interval (CI) = 1.70–2.19) with high heterogeneity (*i* ^2^ = 99.02%).

There was not a clear effect of age. The OR for children was 1.93 (*k* = 31; CI = 1.52–2.46), the OR for adolescents was 2.13 (*k* = 5; CI = 1.75–2.60) and the OR for adults was 1.72 (*k* = 8; CI = 1.40–2.11).

Visual inspection of the funnel plot (Supporting Information S1: Figure S1) showed a tendency towards right asymmetry, which could potentially indicate small‐sample/publication bias. However, the Egger test was not significant (*p* = 0.09). The leave‐one‐out analysis showed that results were robust to the elimination of single studies (Supporting Information S1: Figure S2), likely due to the number of studies included in the analysis. The largest effect was derived from the elimination of Błażewicz et al. (2020), Broder‐Fingert et al. (2014) and M. Metwally et al. (2024) which lowered the mean effect size to 1.88 (CI = 1.66–2.12), 1.86 (CI = 1.66–2.09) and 1.88 (CI = 1.67–2.13) respectively. Community‐based studies (Supporting Information S1: Figure S3) showed a greater risk of obesity (*k* = 36; OR = 1.98; CI = 1.75–2.25; *i* ^2^ = 99.08%) than hospital‐based studies (*k* = 17; OR = 1.79; CI = 1.24–2.58; *i* ^2^ = 78.12%) (Supporting Information S1: Figure S4). When only gold‐standard diagnoses of ASD were considered (Supporting Information S1: Figure S5), the risk was slightly higher than in the main analysis (*k* = 32; OR = 1.97; CI = 1.56–2.48; *i* ^2^ = 79.23%), with the same occurring when only a definition of obesity of pc > 95 or SD > 2 was accepted (*k* = 41; OR = 1.96; CI = 1.65–2.33; *i* ^2^ = 97.60%) (Supporting Information S1: Figure S6).

In the studies without a correction for having zero individuals in a given weight category and group (Supporting Information S1: Figure S7), the OR was 1.92 (*k* = 53; CI = 1.69–2.18; *i* ^2^ = 99.05%), which was higher than when limiting studies to those that had at least five cases per weight category and group (*k* = 43; OR = 1.87; CI = 1.64–2.12; *i* ^2^ = 99.18%) (Supporting Information S1: Figure S8).

The different meta‐regression analyses did not yield any significant results. Neither the sex (male in overall sample, *p* = 0.84, Supporting Information S1: Figure S9; males in ASD sample, *p* = 0.75, Supporting Information S1: Figure S10), and age (*p* = 0.41, Supporting Information S1: Figure S11) was a significant predictor.

#### Overweight

**FIGURE 3:**
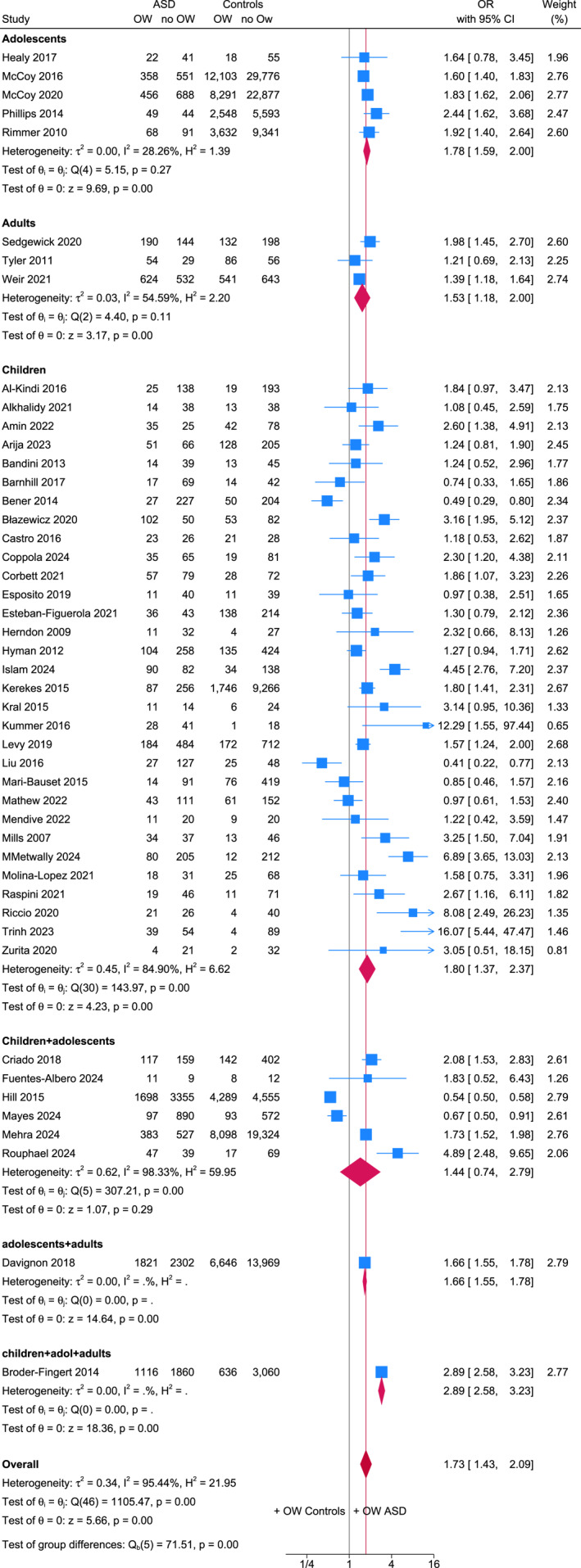
Risk of overweight. Results are presented according to age group. OW: number of individuals with overweight. No OW: number of individuals without overweight. +OW Controls: higher odds of overweight in the control group. +OW ASD: higher odds of overweight in the ASD group.

We found that individuals with ASD had a significant association with overweight, as seen in Figure 3. The overall OR was 1.73 (*k* = 47; CI = 1.43–2.09) with high heterogeneity (*i* ^2^ = 95.44%). Similarly to the risk of obesity, we did not find a clear pattern with age. The OR for children was 1.80 (*k* = 31; CI = 1.37–2.37), the OR for adolescents was 1.78 (*k* = 5; CI = 1.59–2.00) and the OR for adults was 1.53 (*k* = 3; CI = 1.18–2.00).

Visual inspection of the funnel plot (Supporting Information S1: Figure S12) showed a tendency towards right asymmetry, which could potentially indicate small‐sample/publication bias. However, the Egger test was not significant (*p* = 0.03). The leave‐one‐out analysis showed that results were robust to the elimination of single studies (Supporting Information S1: Figure S13), likely due to the number of studies included in the analysis. The biggest effect was derived from the elimination of Islam et al. (2024), Metwally et al. (2024) and Trinh et al. (2023) which lowered the mean effect size to 1.68 (CI = 1.39–2.03), 1.67 (CI = 1.39–2.00) and 1.66 (CI = 1.39–1.99) respectively.

Community‐based studies (Supporting Information S1: Figure S14) showed a greater risk of overweight (*k* = 30; OR = 1.84; CI = 1.49–2.29; *i* ^2^ = 95.14%) than hospital‐based studies (*k* = 15; OR = 1.66; CI = 1.12–2.47; *i* ^2^ = 89.93%) (Supporting Information S1: Figure S15). When only gold‐standard diagnoses of ASD were considered (Supporting Information S1: Figure S16), the risk was slightly lower than in the main analysis (*k* = 35; OR = 1.66; CI = 1.27–2.15; *i* ^2^ = 90.93%), with the same occurring when only a definition of overweight of *p* ≥ 85 or SD ≥ 1 to 1.5 was accepted (*k* = 41; OR = 1.76; CI = 1.46–2.12; *i* ^2^ = 93.08%) (Supporting Information S1: Figure S17).

Removing studies with a correction for having zero individuals in a given weight category and group (Supporting Information S1: Figure S18), led the same risk of overweight as the main analysis (*k* = 47; OR = 1.73; CI = 1.43–2.09; *i* ^2^ = 95.44%), whereas limiting studies to those that had at least five cases per weight category and group, decreased the OR (*k* = 42; OR = 1.60; CI = 1.33–1.91; *i* ^2^ = 95.01%) (Supporting Information S1: Figure S19).

The meta‐regression analysis showed no significant association between sex or age and effect size (male in overall sample, *p* = 0.95, Supporting Information S1: Figure S20; males in ASD sample, *p* = 0.62, Supporting Information S1: Figure S21; age *p* = 0.89, Supporting Information S1: Figure S22).

#### Underweight

**FIGURE 4:**
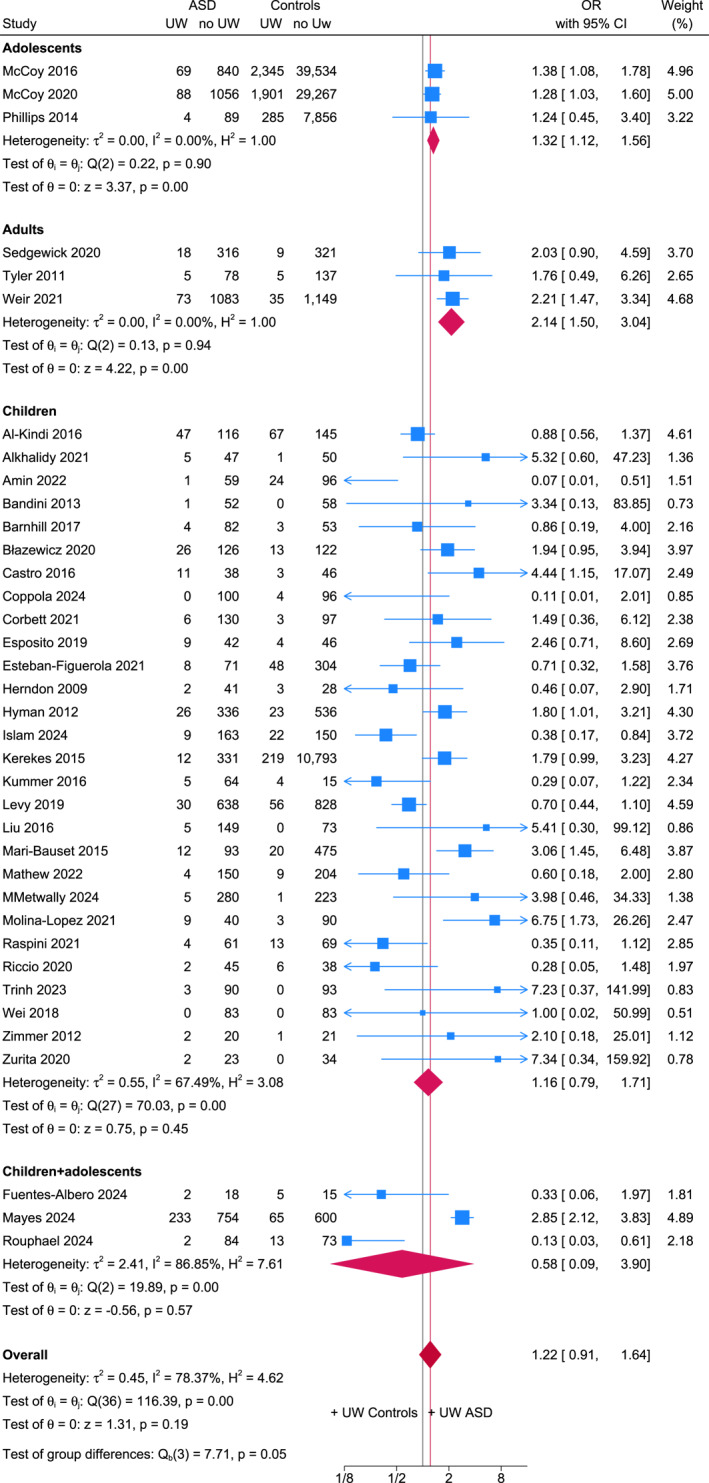
Risk of underweight. Results are presented according to age group. UW: number of individuals with underweight. No UW: number of individuals without underweight. +UW Controls: higher odds of underweight in the control group. +UW ASD: higher odds of underweight in the ASD group.

We did not find evidence for individuals with ASD having a significant association of underweight, as seen in Figure 4. The overall OR was 1.22 (*k* = 37; CI = 0.91–1.64) with substantial heterogeneity (*i* ^2^ = 78.37%). However, there was partial evidence for this risk increasing with age. The effect was lower and not statistically significant in children (*k* = 28; OR = 1.16; CI = 0.79–1.71) but was greater and statistically significant for adolescents (*k* = 3; OR = 1.32; CI = 1.12–1.56) and adults (*k* = 3; OR = 2.14; CI = 1.50–3.04).

Visual inspection of the funnel plot (Supporting Information S1: Figure S23) showed a slight asymmetry, which could potentially indicate a potential publication bias. However, the Egger test was not significant (*p* = 0.60).

The leave‐one‐out analysis (Supporting Information S1: Figure S24) showed that results were robust to the elimination of single studies, likely due to the number of studies included in the analysis. The biggest effect was found after excluding Marí‐Bauset et al. (2015), Mayes et al. (2024) and Molina‐López et al. (2021) which lowered the mean effect size to 1.17 (CI = 0.87–1.58), 1.16 (CI = 0.86–1.57) and 1.17 (CI = 0.87–1.56) respectively.

Community‐based studies (Supporting Information S1: Figure S25) showed a greater risk of underweight (*k* = 21; OR = 1.44; CI = 0.96–2.16; *i* ^2^ = 85.87%) ant the hospital‐based studies (Supporting Information S1: Figure S26) a less risk (*k* = 14; OR = 0.89; CI = 0.54–1.45; *i* ^2^ = 57.85%) than the overall sample, but not significant as well. When only gold‐standard diagnoses of ASD were considered (Supporting Information S1: Figure S27), the risk was slightly lower than in the main analysis (*k* = 30; OR = 1.18; CI = 0.80–1.72; *i* ^2^ = 72.47%), with a similar result when only a definition of underweight of *p* ≤ 5 or SD ≤ −2 was accepted (*k* = 34; OR = 1.14; CI = 0.84–1.56; *i* ^2^ = 76.56%) (Supporting Information S1: Figure S28).

Excluding studies with a correction for having zero individuals in a given weight category and group (Supporting Information S1: Figure S29) led to a lower risk of underweight (*k* = 31; OR = 1.18; CI = 0.87–1.61; *i* ^2^ = 81.35%), whereas limiting studies to those that had at least five cases per in a given weight category and group (Supporting Information S1: Figure S30), increased the risk (*k* = 14; OR = 1.42; CI = 1.06–1.92; *i* ^2^ = 82.13%).

The different meta‐regression analyses did not yield any significant results (male in overall sample, *p* = 0.23, Supporting Information S1: Figure S31; males in ASD sample, *p* = 0.45, Supporting Information S1: Figure S32; age *p* = 0.13, Supporting Information S1: Figure S33).

## DISCUSSION

We investigated meta‐analytically, for the first time, the association between ASD and abnormal weight across the age span. We conducted a systematic review and meta‐analysis pooling data from 64 primary studies, of which 62 provided sufficient data for quantitative synthesis, reporting the frequency of obesity, overweight and/or underweight in ASD and control groups.

Our findings demonstrated that individuals with ASD are at a higher risk of being obese or overweight, with a similar risk of obesity across age groups. Results in relation to underweight were more nuanced: while overall results were not statistically significant, there was evidence for an increased risk in studies with adolescents and adults. Moreover, an overall increased risk of underweight was shown when those studies with a very small number of individuals in a given weight category were eliminated.

Age was thus found to be key factor influencing the relationship between ASD and abnormal weight. As individuals with ASD grow older, changes in lifestyle, autonomy in food choices, and increased exposure to psychotropic medications may contribute to shifting risks. One hypothetical explanation for the age‐related differences in underweight risk is the changing role of caregivers in managing nutrition. In childhood, parents or guardians often play a central role in meal planning, food selection, and monitoring intake, which may help prevent insufficient nutrition despite feeding challenges commonly seen in ASD. As individuals transition into adolescence and adulthood, increased autonomy, reduced supervision, and potential difficulties in self‐regulating dietary habits may contribute to a higher risk of underweight. In contrast, excessive nutrition may be harder for caregivers to control, especially when preferences for energy‐dense foods, limited physical activity, or medication side effects are present, which could lead to excessive weight early in development. Alternatively, age‐related differences may also reflect an underlying genetic vulnerability that becomes more evident later in life.

Our study has several strengths compared to previous meta‐analyses. First, it is, to our knowledge, the most comprehensive to date in terms of weight categories and age coverage, being the first to simultaneously examine obesity, overweight, and underweight across all age groups. Second, our systematic search strategy (combining database queries with reference screening and inclusion of studies reporting on any of the three weight categories) allowed us to identify and include many more primary studies than prior meta‐analyses (Arrondo et al., 2022; Kahathuduwa et al., 2019, 2022; Sammels et al., 2022; Zheng et al., 2017). This broader inclusion enhances the robustness and generalizability of our findings.

Among the key factors limiting the interpretability of our results, heterogeneity of the results in the primary studies, especially in the analyses involving low weight, should be considered. When smaller studies were excluded from the analysis, the level of heterogeneity was reduced. Another limitation was the limited number of studies for some our sub‐analysis and their sample sizes. For example, few studies have been carried out in adults or adolescents, while our results point to this being a key variable.

This study is relevant for understanding the comorbidity between mental and physical health, an area that has gained considerable attention and scientific validation in recent years. According to Cortese et al. (2020), this relationship is complex: in some cases, somatic conditions may contribute to mental disorders, in others, mental conditions may lead to the onset of medical diseases, and in some cases, both somatic and mental conditions share the same risk factors. In the present study, the second scenario is plausible, with the characteristics of ASD contribute to the onset of medical conditions (abnormal weight).

Future studies should analyze if there are individual factors or variables that predict, especially in the long term, the likelihood of individuals of ASD of falling outside the normal limits of weight whether it is over or underweight. Indeed, factors such as medication intake or food selectivity could interact with other individual of social variables to lead to under or overweight in specific individuals. Beyond body weight, future studies should also consider the broader nutritional status of individuals with ASD. Food selectivity is a well‐documented feature in a subgroup of individuals with ASD, who may refuse a broad range of many foods and eat only a very narrow range (Esposito et al., 2023; Marí‐Bauset et al., 2014; Rodrigues et al., 2023). This together with the possible co‐occurrence of eating disorders such as Avoidant/Restrictive Food Intake Disorder (ARFID) (Sader et al., 2025), may lead to significant macro‐ and micronutrient deficiencies even in individuals with a normal body weight. Specifically, children with ASD may show lower intakes of protein, calcium, phosphorus, selenium, omega‐3, and vitamins D, A, B1, B2, B9, and B12 compared with controls (Alhrbi et al., 2025; Esteban‐Figuerola et al., 2019). These atypical eating patterns often emerge early in development and are closely linked to sensory processing difficulties, especially oral, taste, smell and tactile sensitivity (S. Page et al., 2022; Riccio et al., 2025; Rodrigues et al., 2023), and challenging mealtime behaviors, cognitive rigidity, and lower adaptive skills (Mirizzi et al., 2025; S. Page et al., 2022; Sarnataro et al., 2025). The atypical eating patterns often include strong preferences for specific textures, colors, tastes, smells, brand or presentation (Byrska et al., 2023; Mirizzi et al., 2025) and frequent refusal of fruits and vegetables and more limited overall variety (Esposito et al., 2023; Ferrara et al., 2025). As such, they carry important long‐term implications for physical health and overall wellbeing, further underscoring the clinical and public health relevance of monitoring nutritional status, and not merely body weight, in this population. Moreover, for future studies, more detailed stratification variables can be incorporated (e.g., intellectual disability, medication status).

The results of this study have important implications from a clinical and public health standpoint. The finding that people with ASD are at risk of having abnormal weight, and thus an unhealthy weight that negatively affects both their physical and psychological health, has actionable implications. Families and healthcare professionals must be aware of this risk to intervene and promote behaviors that encourage healthy weight, thereby preventing the harmful effects it has on this population's health. This will enhance the well‐being and improve the quality of life for people with ASD and those around them. Parents play an important role in preventing the development of unhealthy weight in children. Given the challenges associated with feeding in this population, it is essential to provide parents with psychoeducation and ongoing support throughout the process. Moreover, qualitative studies of parents of children with ASD highlight unmet needs when seeking weight management support: families want individualized, multidisciplinary support that integrates disability and weight expertise, beyond generic dietary recommendations (McPherson et al., 2022). Parental involvement is particularly critical, as parent‐focused training targeting diet, physical activity, and feeding practices has shown benefits (Markowitz & Buzby, 2021).

Additionally, healthcare professionals are encouraged to offer parents clear guidelines, training, and educational programs. Specifically, nutritional intervention programs or age‐specific training programs on appropriate feeding practices should be considered to address these needs (Eow et al., 2022). In their systematic review about weight management interventions for youth with ASD, Healy et al. (2019) indicated that comprehensive, multidisciplinary programs combining nutrition, physical activity, and behavioral strategies are the most promising approaches, although overall evidence quality remains limited. Similarly, the Healthy Weight Research Network developed recommendations for pediatric primary care for managing overweight and obesity in children with ASD. They emphasized routine BMI screening from age 2 (earlier than recommended for the general pediatric population), non‐stigmatizing, positive, and health‐oriented manner communication with families, and a staged intervention approach adapted to the specific behavioral and sensory profile of this population (Curtin et al., 2020). These recommendations can assist pediatric providers in providing tailored guidance on weight management to children with ASD and their families.

## CONCLUSION

Our systematic review with meta‐analysis summarizes the current evidence supporting that those with ASD have an elevated risk of having an abnormal weight, compared to those without ASD, as well as its connection to individual age. We identified the complex relationship between the disorder and BMI. These findings have implications not only for the clinical practice but also in terms of public health initiatives.

## AUTHOR CONTRIBUTIONS

**Victoria Nieuwenhuys‐Ruiz**: Conceptualization; methodology; investigation; writing—original draft; writing—review and editing. **Leyre Gambra**: Investigation; writing—original draft. **Samuele Cortese**: Conceptualization; writing—review and editing. **Pablo Lizoain**: Investigation; writing—review and editing. **Diana Rodriguez‐Romero**: Writing—review and editing; investigation. **Ursula Paiva**: Investigation; writing—review and editing. **Carmen Gándara**: Investigation; writing—review and editing. **Sara Magallón**: Investigation; writing—review and editing. **Gonzalo Arrondo**: Conceptualization; methodology; formal analysis; investigation; resources; writing—original draft; writing—review and editing; supervision; project administration.

## CONFLICT OF INTEREST STATEMENT

The authors declare no conflicts of interest.

## ETHICAL CONSIDERATIONS

Ethical approval was not required for this study because it is based exclusively on previously published studies and does not involve the collection of data from human participants.

## Supporting information

Supporting Information S1

## Data Availability

The data that supports the findings of this study are available in the supplementary material of this article.

## References

[jcv270151-bib-0001] Ahlberg, R. , Garcia‐Argibay, M. , Hirvikoski, T. , Boman, M. , Chen, Q. , Taylor, M. J. , Frans, E. , Bölte, S. , & Larsson, H. (2022). Shared familial risk factors between autism spectrum disorder and obesity—A register‐based familial coaggregation cohort study. Journal of Child Psychology and Psychiatry, 63(8), 890–899. 10.1111/jcpp.13538 34881437

[jcv270151-bib-0002] Alhrbi, A. , Vlachopoulos, D. , Healey, E. , Massoud, A. T. , Morris, C. , & Revuelta Iniesta, R. (2025). Nutritional status of children diagnosed With autism spectrum disorder: A systematic review and meta‐analysis. Journal of Human Nutrition and Dietetics, 38(4), e70099. 10.1111/jhn.70099 40708203 PMC12290316

[jcv270151-bib-0003] Alkhalidy, H. , Abushaikha, A. , Alnaser, K. , Obeidat, M. D. , & Al‐Shami, I. (2021). Nutritional status of pre‐school children and determinant factors of autism: A case‐control study. Frontiers in Nutrition, 8, 627011. 10.3389/fnut.2021.627011 33681277 PMC7933547

[jcv270151-bib-0004] Al‐Kindi, N. M. , Al‐Farsi, Y. M. , Waly, M. I. , A‐Shafaee, M. S. , & Bakheit, C. S. (2016). Dietary Intake and Food Preferences of Autistic Children Versus Children with Typical Development: A Comparative Cross‐Sectional Study, 6, 72–85.

[jcv270151-bib-0005] Allen, B. , & Saunders, J. (2023). Malnutrition and undernutrition: Causes, consequences, assessment and management. Medicine, 51(7), 461–468. 10.1016/j.mpmed.2023.04.004

[jcv270151-bib-0006] American Psychiatric Association . (2022). Diagnostic and statistical manual of mental disorders (5th ed.). American Psychiatric Publishing.

[jcv270151-bib-0007] Ames, J. L. , Morgan, E. H. , Giwa Onaiwu, M. , Qian, Y. , Massolo, M. L. , & Croen, L. A. (2022). Racial/ethnic differences in psychiatric and medical diagnoses among autistic adults. Autism in Adulthood, 4(4), 290–305. 10.1089/aut.2021.0083 36601333 PMC9807255

[jcv270151-bib-0008] Amin, M. , Faiyaz, S. I. , Butt, M. S. , Javed, A. , Saleem, J. , & Saeed, A. (2022). Food preferences and eating behavior among children with autism spectrum disorder: A causal‐comparativestudy in lahore. Avicenna, 2022(2). 10.5339/avi.2022.9

[jcv270151-bib-0009] Arija, V. , Esteban‐Figuerola, P. , Morales‐Hidalgo, P. , Jardí, C. , & Canals‐Sans, J. (2023). Nutrient intake and adequacy in children with autism spectrum disorder: EPINED epidemiological study. Autism, 27(2), 371–388. 10.1177/13623613221098237 35722960

[jcv270151-bib-0010] Arrondo, G. , Solmi, M. , Dragioti, E. , Eudave, L. , Ruiz‐Goikoetxea, M. , Ciaurriz‐Larraz, A. M. , Magallon, S. , Carvalho, A. F. , Cipriani, A. , Fusar‐Poli, P. , Larsson, H. , Correll, C. U. , & Cortese, S. (2022). Associations between mental and physical conditions in children and adolescents: An umbrella review. Neuroscience and Biobehavioral Reviews, 137, 104662. 10.1016/j.neubiorev.2022.104662 35427644

[jcv270151-bib-0011] Bandini, L. G. , Gleason, J. , Curtin, C. , Lividini, K. , Anderson, S. E. , Cermak, S. A. , Maslin, M. , & Must, A. (2013). Comparison of physical activity between children with autism spectrum disorders and typically developing children. Autism, 17(1), 44–54. 10.1177/1362361312437416 22807562 PMC3690470

[jcv270151-bib-0012] Barnhill, K. , Gutierrez, A. , Ghossainy, M. , Marediya, Z. , Marti, C. N. , & Hewitson, L. (2017). Growth status of children with autism spectrum disorder: A case–control study. Journal of Human Nutrition and Dietetics, 30(1), 59–65. 10.1111/jhn.12396 27412698

[jcv270151-bib-0013] Bener, A. , Khattab, A. , & Al‐Dabbagh, M. (2014). Is high prevalence of vitamin D deficiency evidence for autism disorder?: In a highly endogamous population. Journal of Pediatric Neurosciences, 9(3), 227. 10.4103/1817-1745.147574 25624924 PMC4302541

[jcv270151-bib-0014] Błażewicz, A. , Szymańska, I. , Dolliver, W. , Suchocki, P. , Turło, J. , Makarewicz, A. , & Skórzyńska‐Dziduszko, K. (2020). Are Obese patients with autism spectrum disorder more likely to be selenium deficient? Research findings on Pre‐ and post‐pubertal children. Nutrients, 12(11), 3581. 10.3390/nu12113581 33266486 PMC7700552

[jcv270151-bib-0015] Bora, N. , K, V. , Verma, A. , Bharti, A. K. , & Sinha, M. K. (2024). Physical activity and sedentary behavior perceptions in overweight and Obese adults: A systematic review of qualitative study. F1000Research, 13, 787. 10.12688/f1000research.152905.1 39131836 PMC11316175

[jcv270151-bib-0016] Bray, G. A. , Heisel, W. E. , Afshin, A. , Jensen, M. D. , Dietz, W. H. , Long, M. , Kushner, R. F. , Daniels, S. R. , Wadden, T. A. , Tsai, A. G. , Hu, F. B. , Jakicic, J. M. , Ryan, D. H. , Wolfe, B. M. , & Inge, T. H. (2018). The science of obesity management: An endocrine society scientific statement. Endocrine Reviews, 39(2), 132. 10.1210/er.2017-00253 PMC5888222 29518206

[jcv270151-bib-0017] Broder‐Fingert, S. , Brazauskas, K. , Lindgren, K. , Iannuzzi, D. , & Van Cleave, J. (2014). Prevalence of overweight and obesity in a large clinical sample of children with autism. Academic Pediatrics, 14(4), 408–414. 10.1016/j.acap.2014.04.004 24976353

[jcv270151-bib-0018] Buro, A. W. , Salinas‐Miranda, A. , Marshall, J. , Gray, H. L. , & Kirby, R. S. (2022). Correlates of obesity in adolescents with and without autism spectrum disorder: The 2017–2018 national survey of Children’s health. Disability and Health Journal, 15(2), 101221. 10.1016/j.dhjo.2021.101221 34654677

[jcv270151-bib-0019] Byrska, A. , Błażejczyk, I. , Faruga, A. , Potaczek, M. , Wilczyński, K. M. , & Janas‐Kozik, M. (2023). Patterns of food selectivity among children with autism spectrum disorder. Journal of Clinical Medicine, 12(17), 5469. 10.3390/jcm12175469 37685537 PMC10488249

[jcv270151-bib-0020] Castro, K. , Faccioli, L. S. , Baronio, D. , Gottfried, C. , Perry, I. S. , & Riesgo, R. (2016). Feeding behavior and dietary intake of Male children and adolescents with autism spectrum disorder: A case‐control study. International Journal of Developmental Neuroscience, 53(1), 68–74. 10.1016/j.ijdevneu.2016.07.003 27432261

[jcv270151-bib-0021] Chen, M.‐H. , Lan, W.‐H. , Hsu, J.‐W. , Huang, K.‐L. , Su, T.‐P. , Li, C.‐T. , Lin, W.‐C. , Tsai, C.‐F. , Tsai, S.‐J. , Lee, Y.‐C. , Chen, Y.‐S. , Pan, T.‐L. , Chang, W.‐H. , Chen, T.‐J. , & Bai, Y.‐M. (2016). Risk of developing type 2 diabetes in adolescents and young adults with autism spectrum disorder: A nationwide longitudinal study. Diabetes Care, 39(5), 788–793. 10.2337/dc15-1807 27006513

[jcv270151-bib-0022] Coppola, S. , Nocerino, R. , Oglio, F. , Golia, P. , Falco, M. C. , Riccio, M. P. , Carucci, L. , Rea, T. , Simeone, S. , Garotti, R. , Marani, N. , Bravaccio, C. , & Canani, R. B. (2024). Adverse food reactions and alterations in nutritional status in children with autism spectrum disorders: Results of the NAFRA project. Italian Journal of Pediatrics, 50(1), 228. 10.1186/s13052-024-01794-8 39497088 PMC11533279

[jcv270151-bib-0023] Corbett, B. A. , Muscatello, R. A. , Horrocks, B. K. , Klemencic, M. E. , & Tanguturi, Y. (2021). Differences in body mass index (BMI) in early adolescents with autism spectrum disorder compared to youth with typical development. Journal of Autism and Developmental Disorders, 51(8), 2790–2799. 10.1007/s10803-020-04749-0 33051783 PMC8041918

[jcv270151-bib-0024] Correll, C. U. (2009). Cardiometabolic risk of second‐generation antipsychotic medications during first‐time use in children and adolescents. JAMA, 302(16), 1765. 10.1001/jama.2009.1549 19861668 PMC3055794

[jcv270151-bib-0025] Cortese, S. , Bellato, A. , Gabellone, A. , Marzulli, L. , Matera, E. , Parlatini, V. , Petruzzelli, M. G. , Persico, A. M. , Delorme, R. , Fusar‐Poli, P. , Gosling, C. J. , Solmi, M. , & Margari, L. (2025). Latest clinical frontiers related to autism diagnostic strategies. Cell Reports Medicine, 6(2), 101916. 10.1016/j.xcrm.2024.101916 39879991 PMC11866554

[jcv270151-bib-0026] Cortese, S. , Gabellone, A. , Marzulli, L. , Iturmendi‐Sabater, I. , De La Chica‐Duarte, D. , Piqué, I. M. , Solmi, M. , Shin, J. I. , Margari, L. , & Arrondo, G. (2022). Association between autism spectrum disorder and diabetes: Systematic review and meta‐analysis. Neuroscience and Biobehavioral Reviews, 136, 104592. 10.1016/j.neubiorev.2022.104592 35217107

[jcv270151-bib-0027] Cortese, S. , Solmi, M. , Arrondo, G. , Cipriani, A. , Fusar‐Poli, P. , Larsson, H. , & Correll, C. (2020). Association between mental disorders and somatic conditions: Protocol for an umbrella review. Evidence‐Based Mental Health, 23(4), 135–139. 10.1136/ebmental-2020-300158 32900790 PMC10231570

[jcv270151-bib-0028] Covidence Systematic Review Software . (2025). Covidence [Logiciel]. Retrieved from https://www.covidence.org

[jcv270151-bib-0029] Criado, K. K. , Sharp, W. G. , McCracken, C. E. , De Vinck‐Baroody, O. , Dong, L. , Aman, M. G. , McDougle, C. J. , McCracken, J. T. , Eugene Arnold, L. , Weitzman, C. , Leventhal, J. M. , Vitiello, B. , & Scahill, L. (2018). Overweight and Obese status in children with autism spectrum disorder and disruptive behavior. Autism, 22(4), 450–459. 10.1177/1362361316683888 28325061 PMC5581311

[jcv270151-bib-0030] Croen, L. A. , Zerbo, O. , Qian, Y. , Massolo, M. L. , Rich, S. , Sidney, S. , & Kripke, C. (2015). The health status of adults on the autism spectrum. Autism, 19(7), 814–823. 10.1177/1362361315577517 25911091

[jcv270151-bib-0031] Curtin, C. , Anderson, S. E. , Must, A. , & Bandini, L. (2010). The prevalence of obesity in children with autism: A secondary data analysis using nationally representative data from the national survey of Children’s health. BMC Pediatrics, 10(1), 11. 10.1186/1471-2431-10-11 20178579 PMC2843677

[jcv270151-bib-0032] Curtin, C. , Hyman, S. L. , Boas, D. D. , Hassink, S. , Broder‐Fingert, S. , Ptomey, L. T. , Gillette, M. D. , Fleming, R. K. , Must, A. , & Bandini, L. G. (2020). Weight management in primary care for children with autism: Expert recommendations. Pediatrics, 145(Supplement_1), S126–S139. 10.1542/peds.2019-1895P 32238539

[jcv270151-bib-0033] Davignon, M. N. , Qian, Y. , Massolo, M. , & Croen, L. A. (2018). Psychiatric and medical conditions in transition‐aged individuals with ASD. Pediatrics, 141(Supplement_4), S335–S345. 10.1542/peds.2016-4300K 29610415

[jcv270151-bib-0034] Dell’Osso, L. , Carpita, B. , Gesi, C. , Cremone, I. M. , Corsi, M. , Massimetti, E. , Muti, D. , Calderani, E. , Castellini, G. , Luciano, M. , Ricca, V. , Carmassi, C. , & Maj, M. (2018). Subthreshold autism spectrum disorder in patients with eating disorders. Comprehensive Psychiatry, 81, 66–72. 10.1016/j.comppsych.2017.11.007 29268154

[jcv270151-bib-0035] Dhaliwal, K. K. , Orsso, C. E. , Richard, C. , Haqq, A. M. , & Zwaigenbaum, L. (2019). Risk factors for unhealthy weight gain and obesity among children with autism spectrum disorder. International Journal of Molecular Sciences, 20(13), 3285. 10.3390/ijms20133285 31277383 PMC6650879

[jcv270151-bib-0036] Dhanasekara, C. S. , Ancona, D. , Cortes, L. , Hu, A. , Rimu, A. H. , Robohm‐Leavitt, C. , Payne, D. , Wakefield, S. M. , Mastergeorge, A. M. , & Kahathuduwa, C. N. (2023). Association between autism spectrum disorders and cardiometabolic diseases: A systematic review and meta‐analysis. JAMA Pediatrics, 177(3), 248. 10.1001/jamapediatrics.2022.5629 36716018 PMC9887535

[jcv270151-bib-0037] Eliasziw, M. , Kral, T. V. E. , Segal, M. , Sikich, L. , Phillips, S. , Tybor, D. J. , Bandini, L. G. , Curtin, C. , & Must, A. (2021). Healthy‐weight kindergarten children with autism spectrum disorder may become overweight and Obese during the first few years of elementary school. The Journal of Pediatrics, X(7), 100074. 10.1016/j.ympdx.2021.100074 PMC10236540 37333885

[jcv270151-bib-0038] Enomoto, A. , Saito, A. , Takahashi, O. , Kimura, T. , Tajima, R. , Rahman, M. , & Iida, K. (2020). Associations between health literacy and underweight and overweight among Japanese adults aged 20 to 39 years: A cross‐sectional study. Health Education and Behavior, 47(4), 631‐639–639. 10.1177/1090198120919675 32449373

[jcv270151-bib-0039] Eow, S. Y. , Gan, W. Y. , Lim, P. Y. , Awang, H. , & Mohd Shariff, Z. (2022). Parental feeding practices and child‐related factors are associated with overweight and obesity in children and adolescents with autism spectrum disorder. Journal of Autism and Developmental Disorders, 52(8), 3655–3667. 10.1007/s10803-021-05247-7 34453670

[jcv270151-bib-0040] Eslick, G. D. (2012). Gastrointestinal symptoms and obesity: A meta‐analysis. Obesity Reviews, 13(5), 469–479. 10.1111/j.1467-789X.2011.00969.x 22188520

[jcv270151-bib-0041] Esposito, M. , Mirizzi, P. , Fadda, R. , Pirollo, C. , Ricciardi, O. , Mazza, M. , & Valenti, M. (2023). Food selectivity in children with autism: Guidelines for assessment and clinical interventions. International Journal of Environmental Research and Public Health, 20(6), 5092. 10.3390/ijerph20065092 36982001 PMC10048794

[jcv270151-bib-0042] Esposito, M. , Sloan, J. , Nappo, R. , Fadda, R. , Fotia, F. , Napoli, E. , Mazzone, L. , Valeri, G. , & Vicari, S. (2019). Sensory processing, gastrointestinal symptoms and parental feeding practices in the explanation of food selectivity: Clustering children with and without autism (Vol. 120).

[jcv270151-bib-0043] Esteban‐Figuerola, P. , Canals, J. , Fernández‐Cao, J. C. , & Arija Val, V. (2019). Differences in food consumption and nutritional intake between children with autism spectrum disorders and typically developing children: A meta‐analysis. Autism, 23(5), 1079–1095. 10.1177/1362361318794179 30345784

[jcv270151-bib-0044] Esteban‐Figuerola, P. , Morales‐Hidalgo, P. , Arija‐Val, V. , & Canals‐Sans, J. (2021). Are there anthropometric and body composition differences between children with autism spectrum disorder and children with typical development? Analysis by age and spectrum severity in a school population. Autism, 25(5), 1307–1320. 10.1177/1362361320987724 33487005

[jcv270151-bib-0045] Evans, E. W. , Must, A. , Anderson, S. E. , Curtin, C. , Scampini, R. , Maslin, M. , & Bandini, L. (2012). Dietary patterns and body mass index in children with autism and typically developing children. Research in Autism Spectrum Disorders, 6(1), 399–405. 10.1016/j.rasd.2011.06.014 22936951 PMC3427936

[jcv270151-bib-0046] Ferrara, R. , Iovino, L. , Ricci, L. , Avallone, A. , Latina, R. , & Ricci, P. (2025). Food selectivity and autism: A systematic review. World Journal of Clinical Pediatrics, 14(3). 10.5409/wjcp.v14.i3.101974 PMC12304907 40881072

[jcv270151-bib-0047] Figorilli, M. , Velluzzi, F. , & Redolfi, S. (2025). Obesity and sleep disorders: A bidirectional relationship. Nutrition, Metabolism, and Cardiovascular Diseases, 35(6), 104014. 10.1016/j.numecd.2025.104014 40180826

[jcv270151-bib-0048] Flygare Wallén, E. , Ljunggren, G. , Carlsson, A. C. , Pettersson, D. , & Wändell, P. (2018). High prevalence of diabetes mellitus, hypertension and obesity among persons with a recorded diagnosis of intellectual disability or autism spectrum disorder. Journal of Intellectual Disability Research, 62(4), 269–280. 10.1111/jir.12462 29280230

[jcv270151-bib-0049] Fuentes‐Albero, M. , Mafla‐España, M. A. , Martínez‐Raga, J. , & Cauli, O. (2024). Autistic children/adolescents have lower adherence to the mediterranean diet and higher salivary IL‐6 concentration: Potential diet–inflammation links? Pathophysiology, 31(3), 376–387. 10.3390/pathophysiology31030028 39189164 PMC11348102

[jcv270151-bib-0050] Gambra, L. , Cortese, S. , Lizoain, P. , Romero, D. R. , Paiva, U. , Gándara, C. , Arrondo, G. , & Magallón, S. (2024). Excessive body weight in developmental coordination disorder: A systematic review and meta‐analysis. Neuroscience and Biobehavioral Reviews, 164, 105806. 10.1016/j.neubiorev.2024.105806 38986892

[jcv270151-bib-0051] Garcia, J. M. , & Odahowski, C. L. (2023). An urban versus rural comparison of obesity between youth with and without autism spectrum disorder. Autism Research, 16(1), 200–207. 10.1002/aur.2856 36412055

[jcv270151-bib-0052] Hammouda, S. A. I. , Al areefy, A. A. E. H. , Al‐Thbiany, A. , Farghal, S. , Al‐Harbi, G. , Abduallah, M. , Al‐Rehaly, R. , & Al‐Johani, G. (2018). Assessment of nutritional risk factors predisposing to autism among Saudi children (Vol. 17, pp. 23–32).

[jcv270151-bib-0053] Hand, B. N. , Angell, A. M. , Harris, L. , & Carpenter, L. A. (2020). Prevalence of physical and mental health conditions in Medicare‐enrolled, autistic older adults. Autism, 24(3), 755–764. 10.1177/1362361319890793 31773968 PMC7433648

[jcv270151-bib-0054] Healy, S. , Haegele, J. A. , Grenier, M. , & Garcia, J. M. (2017). Physical activity, screen‐time behavior, and obesity among 13‐Year olds in Ireland with and without autism spectrum disorder. Journal of Autism and Developmental Disorders, 47(1), 49–57. 10.1007/s10803-016-2920-4 27671801

[jcv270151-bib-0055] Healy, S. , Pacanowski, C. R. , & Williams, E. (2019). Weight management interventions for youth with autism spectrum disorder: A systematic review. International Journal of Obesity, 43(1), 1–12. 10.1038/s41366-018-0233-8 30305689

[jcv270151-bib-0056] Herndon, A. C. , DiGuiseppi, C. , Johnson, S. L. , Leiferman, J. , & Reynolds, A. (2009). Does nutritional intake differ between children with autism spectrum disorders and children with typical development? Journal of Autism and Developmental Disorders, 39(2), 212–222. 10.1007/s10803-008-0606-2 18600441

[jcv270151-bib-0057] Hill, A. P. , Zuckerman, K. E. , & Fombonne, E. (2015). Obesity and autism. Pediatrics, 136(6), 1051–1061. 10.1542/peds.2015-1437 26527551 PMC4657601

[jcv270151-bib-0058] Hyman, S. L. , Stewart, P. A. , Schmidt, B. , Cain, U. , Lemcke, N. , Foley, J. T. , Peck, R. , Clemons, T. , Reynolds, A. , Johnson, C. , Handen, B. , James, S. J. , Courtney, P. M. , Molloy, C. , & Ng, P. K. (2012). Nutrient intake from food in children with autism. Pediatrics, 130(2), 145–153. 10.1542/peds.2012-0900L PMC4536585 23118245

[jcv270151-bib-0059] Islam, M. N. , Chowdhury, S. R. , Kabir, H. , Bari, F. S. , & Hossain, A. (2024). Dietary diversity and nutritional status of children with and without autism spectrum disorder: A comparative cross‐sectional study in Bangladesh. Discover Social Science and Health, 4(1), 76. 10.1007/s44155-024-00140-x

[jcv270151-bib-0060] Kahathuduwa, C. N. , Dhanasekara, C. S. , Wakefield, S. , Moustaid‐Moussa, N. , & Mastergeorge, A. (2022). Autism spectrum disorder is associated with an increased risk of development of underweight in children and adolescents: A systematic review and meta‐analysis. Research in Autism Spectrum Disorders, 94, 101969. 10.1016/j.rasd.2022.101969

[jcv270151-bib-0061] Kahathuduwa, C. N. , West, B. D. , Blume, J. , Dharavath, N. , Moustaid‐Moussa, N. , & Mastergeorge, A. (2019). The risk of overweight and obesity in children with autism spectrum disorders: A systematic review and meta‐analysis. Obesity Reviews, 20(12), 1667–1679. 10.1111/obr.12933 31595678

[jcv270151-bib-0062] Kerekes, N. , Tajnia, A. , Lichtenstein, P. , Lundström, S. , Anckarsäter, H. , Nilsson, T. , & Råstam, M. (2015). Neurodevelopmental problems and extremes in BMI. PeerJ, 3, e1024. 10.7717/peerj.1024 26207189 PMC4511820

[jcv270151-bib-0063] Kolotkin, R. L. , & Andersen, J. R. (2017). A systematic review of reviews: Exploring the relationship between obesity, weight loss and health‐related quality of life. Clinical Obesity, 7(5), 273–289. 10.1111/cob.12203 28695722 PMC5600094

[jcv270151-bib-0064] Kral, T. V. E. , Souders, M. C. , Tompkins, V. H. , Remiker, A. M. , Eriksen, W. T. , & Pinto‐Martin, J. A. (2015). Child eating behaviors and caregiver feeding practices in children with autism spectrum disorders. Public Health Nursing, 32(5), 488–497. 10.1111/phn.12146 25112438

[jcv270151-bib-0065] Kummer, A. , Barbosa, I. G. , Rodrigues, D. H. , Rocha, N. P. , Da Silva Rafael, M. , Pfeilsticker, L. , Simõese Silva, A. C. , & Teixeira, A. L. (2016). Frequency of overweight and obesity in children and adolescents with autism and attention deficit/hyperactivity disorder. Revista Paulista de Pediatria, 34(1), 71–77. 10.1016/j.rppede.2015.12.006 26525687 PMC4795724

[jcv270151-bib-0066] Lai, M.‐C. , Kassee, C. , Besney, R. , Bonato, S. , Hull, L. , Mandy, W. , Szatmari, P. , & Ameis, S. H. (2019). Prevalence of co‐occurring mental health diagnoses in the autism population: A systematic review and meta‐analysis. The Lancet Psychiatry, 6(10), 819–829. 10.1016/S2215-0366(19)30289-5 31447415

[jcv270151-bib-0067] Levy, S. E. , Pinto‐Martin, J. A. , Bradley, C. B. , Chittams, J. , Johnson, S. L. , Pandey, J. , Pomykacz, A. , Ramirez, A. , Reynolds, A. , Rubenstein, E. , Schieve, L. A. , Shapira, S. K. , Thompson, A. , Young, L. , & Kral, T. V. E. (2019). Relationship of weight outcomes, Co‐Occurring conditions, and severity of autism spectrum disorder in the study to explore early development. The Journal of Pediatrics, 205, 202–209. 10.1016/j.jpeds.2018.09.003 30314662 PMC6348122

[jcv270151-bib-0068] Li, H. , Liu, H. , Chen, X. , Zhang, J. , Tong, G. , & Sun, Y. (2021). Association of food hypersensitivity in children with the risk of autism spectrum disorder: A meta‐analysis. European Journal of Pediatrics, 180(4), 999–1008. 10.1007/s00431-020-03826-x 33145704

[jcv270151-bib-0069] Liu, X. , Liu, J. , Xiong, X. , Yang, T. , Hou, N. , Liang, X. , Chen, J. , Cheng, Q. , & Li, T. (2016). Correlation between nutrition and symptoms: Nutritional survey of children with autism spectrum disorder in chongqing, China. Nutrients, 8(5), 294. 10.3390/nu8050294 27187463 PMC4882707

[jcv270151-bib-0070] Lorem, G. F. , Schirmer, H. , & Emaus, N. (2017). What is the impact of underweight on self‐reported health trajectories and mortality rates: A cohort study. Health and Quality of Life Outcomes, 15(1), 191. 10.1186/s12955-017-0766-x 28969649 PMC5625617

[jcv270151-bib-0071] Marí‐Bauset, S. , Llopis‐González, A. , Zazpe, I. , Mari‐Sanchis, A. , & Morales‐Suárez‐Varela, M. (2015). Anthropometric measures of Spanish children with autism spectrum disorder. Research in Autism Spectrum Disorders, 9, 26–33. 10.1016/j.rasd.2014.09.013

[jcv270151-bib-0072] Marí‐Bauset, S. , Zazpe, I. , Mari‐Sanchis, A. , Llopis‐González, A. , & Morales‐Suárez‐Varela, M. (2014). Food selectivity in autism spectrum disorders: A systematic review. Journal of Child Neurology, 29(11), 1554–1561. 10.1177/0883073813498821 24097852

[jcv270151-bib-0073] Markowitz, G. , & Buzby, M. (2021). Weighing in on children with autism: Rethinking strategies for weight management. Journal of Pediatric Nursing, 59, 209–210. 10.1016/j.pedn.2021.04.015 33934938

[jcv270151-bib-0074] Mathew, N. E. , Mallitt, K. , Masi, A. , Katz, T. , Walker, A. K. , Morris, M. J. , & Ooi, C. Y. (2022). Dietary intake in children on the autism spectrum is altered and linked to differences in autistic traits and sensory processing styles. Autism Research, 15(10), 1824–1839. 10.1002/aur.2798 36054787 PMC9804726

[jcv270151-bib-0075] Mayes, S. D. , Calhoun, S. L. , Kallus, R. , Baweja, R. , & Waschbusch, D. A. (2024). Cognitive disengagement syndrome (formerly sluggish cognitive tempo) and comorbid symptoms in child autism, ADHD, and elementary school samples. Journal of Psychopathology and Behavioral Assessment, 46(3), 857–865. 10.1007/s10862-024-10145-0

[jcv270151-bib-0076] McCoy, S. M. , Jakicic, J. M. , & Gibbs, B. B. (2016). Comparison of obesity, physical activity, and sedentary behaviors between adolescents with autism spectrum disorders and without. Journal of Autism and Developmental Disorders, 46(7), 2317–2326. 10.1007/s10803-016-2762-0 26936162

[jcv270151-bib-0077] McCoy, S. M. , & Morgan, K. (2020). Obesity, physical activity, and sedentary behaviors in adolescents with autism spectrum disorder compared with typically developing peers. Autism, 24(2), 387–399. 10.1177/1362361319861579 31364386

[jcv270151-bib-0078] McPherson, A. C. , Perez, A. , Buchholz, A. , Forhan, M. , & Ball, G. D. C. (2022). “It’s not a simple answer.” A qualitative study to explore how healthcare providers can best support families with a child with autism spectrum disorder and overweight or obesity. Disability & Rehabilitation, 44(14), 3540–3546. 10.1080/09638288.2020.1867909 33399017

[jcv270151-bib-0079] Mehra, S. , Salinas‐Miranda, A. A. , Buro, A. W. , Marshall, J. , & Kirby, R. S. (2024). The role of adverse childhood experiences in obesity among adolescents with autism spectrum disorder: National survey of Children’s health 2018‐2019. Disability and Health Journal, 17(2), 101550. 10.1016/j.dhjo.2023.101550 37968201

[jcv270151-bib-0080] Mendive Dubourdieu, P. , & Guerendiain, M. (2022). Dietary intake, nutritional status and sensory profile in children with autism spectrum disorder and typical development. Nutrients, 14(10), 2155. 10.3390/nu14102155 35631297 PMC9147144

[jcv270151-bib-0081] Metwally, M. , Helmy, M. , Aboulghate, A. , Abu‐Mandil Hassan, N. , S. Mahmoud, W. , Ahmed, H. , Mabrok, B. , Elshaarawy, G. , Elsaied, A. A. , Ashaat, E. , El Rifay, S. , Abdelhady, S. E. , Eldeeb, S. , Abdelhady, S. , M. El‐Saied, M. , A. El‐Masry, S. , E Hassan, N. , & M. Alian, K. (2024). The odds of having obesity in Egyptian children with autism spectrum disorders is higher than stunting compared to healthy developing peers: A national survey. BMC Pediatrics, 24(1), 465. 10.1186/s12887-024-04934-5 39033272 PMC11264811

[jcv270151-bib-0082] Micai, M. , Fatta, L. M. , Gila, L. , Caruso, A. , Salvitti, T. , Fulceri, F. , Ciaramella, A. , D’Amico, R. , Del Giovane, C. , Bertelli, M. , Romano, G. , Schünemann, H. J. , & Scattoni, M. L. (2023). Prevalence of co‐occurring conditions in children and adults with autism spectrum disorder: A systematic review and meta‐analysis. Neuroscience and Biobehavioral Reviews, 155, 105436. 10.1016/j.neubiorev.2023.105436 37913872

[jcv270151-bib-0083] Mills, J. L. , Hediger, M. L. , Molloy, C. A. , Chrousos, G. P. , Manning‐Courtney, P. , Yu, K. F. , Brasington, M. , & England, L. J. (2007). Elevated levels of growth‐related hormones in autism and autism spectrum disorder. Clinical Endocrinology, 67(2), 230–237. 10.1111/j.1365-2265.2007.02868.x 17547689

[jcv270151-bib-0084] Mirizzi, P. , Esposito, M. , Ricciardi, O. , Bove, D. , Fadda, R. , Caffò, A. O. , Mazza, M. , & Valenti, M. (2025). Food selectivity in children with autism spectrum disorder and in typically developing peers: Sensory processing, parental practices, and gastrointestinal symptoms. Nutrients, 17(17), 2798. 10.3390/nu17172798 40944186 PMC12430742

[jcv270151-bib-0085] Molina‐López, J. , Leiva‐García, B. , Planells, E. , & Planells, P. (2021). Food selectivity, nutritional inadequacies, and mealtime behavioral problems in children with autism spectrum disorder compared to neurotypical children. International Journal of Eating Disorders, 54(12), 2155–2166. 10.1002/eat.23631 34704615

[jcv270151-bib-0086] Must, A. , Eliasziw, M. , Bowling, A. , Magaña, S. , Stanish, H. , Bandini, L. , Curtin, C. , & Kral, T. V. E. (2023). Relationship between childhood obesity and autism spectrum disorder varies by child’s age and parents’ weight status in a sample of sibling dyads. Childhood Obesity, 19(5), 309–315. 10.1089/chi.2022.0064 35994016

[jcv270151-bib-0087] Must, A. , Eliasziw, M. , Stanish, H. , Curtin, C. , Bandini, L. G. , & Bowling, A. (2023b). Passive and social screen time in children with autism and in association with obesity. Frontiers in Pediatrics, 11, 1198033. 10.3389/fped.2023.1198033 37492602 PMC10364473

[jcv270151-bib-0088] Page, M. J. , McKenzie, J. E. , Bossuyt, P. M. , Boutron, I. , Hoffmann, T. C. , Mulrow, C. D. , Shamseer, L. , Tetzlaff, J. M. , Akl, E. A. , Brennan, S. E. , Chou, R. , Glanville, J. , Grimshaw, J. M. , Hróbjartsson, A. , Lalu, M. M. , Li, T. , Loder, E. W. , Mayo‐Wilson, E. , McDonald, S. , … Whiting, P. (2021). The PRISMA 2020 statement: An updated guideline for reporting systematic reviews. BMJ, n71, n71. 10.1136/bmj.n71 PMC8005924 33782057

[jcv270151-bib-0089] Page, S. , Souders, M. C. , Kral, T. V. E. , Chao, A. M. , & Pinto‐Martin, J. (2022). Correlates of feeding difficulties among children with autism spectrum disorder: A systematic review. Journal of Autism and Developmental Disorders, 52(1), 255–274. 10.1007/s10803-021-04947-4 33666799

[jcv270151-bib-0090] Petruzzelli, M. G. , Matera, E. , Margari, L. , Marzulli, L. , Gabellone, A. , Cotugno, C. , Annecchini, F. , & Cortese, S. (2026). An update on the comorbidity of attention deficit/hyperactivity disorder (ADHD) and autism spectrum disorder (ASD) and its clinical management. Expert Review of Neurotherapeutics, 26(1), 75–89. 10.1080/14737175.2025.2599856 41388592

[jcv270151-bib-0091] Phillips, K. L. , Schieve, L. A. , Visser, S. , Boulet, S. , Sharma, A. J. , Kogan, M. D. , Boyle, C. A. , & Yeargin‐Allsopp, M. (2014). Prevalence and impact of unhealthy weight in a national sample of US adolescents with autism and other learning and behavioral disabilities. Maternal and Child Health Journal, 18(8), 1964–1975. 10.1007/s10995-014-1442-y 24553796 PMC5328414

[jcv270151-bib-0092] Raspini, B. , Prosperi, M. , Guiducci, L. , Santocchi, E. , Tancredi, R. , Calderoni, S. , Morales, M. A. , Morelli, M. , Simione, M. , Fiechtner, L. , Muratori, F. , & Cena, H. (2021). Dietary patterns and weight status in Italian preschoolers with autism spectrum disorder and typically developing children. Nutrients, 13(11), 4039. 10.3390/nu13114039 34836294 PMC8617730

[jcv270151-bib-0093] Riccio, M. P. , Catone, G. , Siracusano, R. , Occhiati, L. , Bernardo, P. , Sarnataro, E. , Corrado, G. , & Bravaccio, C. (2020). Vitamin D deficiency is not related to eating habits in children with autistic spectrum disorder. AIMS Public Health, 7(4), 792–803. 10.3934/publichealth.2020061 33294482 PMC7719555

[jcv270151-bib-0094] Riccio, M. P. , Marino, M. , Garotti, R. , Tassiello, A. , Maffettone, V. , Pezone, M. , & Bravaccio, C. (2025). Food selectivity in autism spectrum disorder: Implications of eating, sensory and behavioural profile. Frontiers in Psychiatry, 16, 1587454. 10.3389/fpsyt.2025.1587454 40530068 PMC12171170

[jcv270151-bib-0095] Rimmer, J. H. , Yamaki, K. , Lowry, D. M. D. , Wang, E. , & Vogel, L. C. (2010). Obesity and obesity‐related secondary conditions in adolescents with intellectual/developmental disabilities. Journal of Intellectual Disability Research, 54(9), 787–794. 10.1111/j.1365-2788.2010.01305.x 20630017

[jcv270151-bib-0096] Roberts, M. , Tolar‐Peterson, T. , Reynolds, A. , Wall, C. , Reeder, N. , & Rico Mendez, G. (2022). The effects of nutritional interventions on the cognitive development of preschool‐age children: A systematic review. Nutrients, 14(3), 532. 10.3390/nu14030532 35276891 PMC8839299

[jcv270151-bib-0097] Rodrigues, J. , Poli, M. , Petrilli, P. , Dornelles, R. , Turcio, K. , & Theodoro, L. (2023). Food selectivity and neophobia in children with autism spectrum disorder and neurotypical development: A systematic review. Nutrition Reviews, 81(8), 1034–1050. 10.1093/nutrit/nuac112 36633300

[jcv270151-bib-0098] Rouphael, M. , Sacre, Y. , Bitar, T. , Andres, C. R. , & Hleihel, W. (2024). Body composition and anthropometric measurements in children and adolescents with autism spectrum disorder: A case–control study in Lebanon. Nutrients, 16(6), 847. 10.3390/nu16060847 38542757 PMC10976266

[jcv270151-bib-0099] Sader, M. , Weston, A. , Buchan, K. , Kerr‐Gaffney, J. , Gillespie‐Smith, K. , Sharpe, H. , & Duffy, F. (2025). The co‐occurrence of autism and avoidant/restrictive food intake disorder (ARFID): A prevalence‐based meta‐analysis. International Journal of Eating Disorders, 58(3), 473–488. 10.1002/eat.24369 39760303 PMC11891632

[jcv270151-bib-0100] Sammels, O. , Karjalainen, L. , Dahlgren, J. , & Wentz, E. (2022). Autism spectrum disorder and obesity in children: A systematic review and meta‐analysis. Obesity Facts, 15(3), 305–320. 10.1159/000523943 35263756 PMC9210004

[jcv270151-bib-0101] Sarnataro, R. , Siracusano, M. , Campanile, R. , Marcovecchio, C. , Babolin, S. , Riccioni, A. , Arturi, L. , & Mazzone, L. (2025). Relationship between food selectivity, adaptive functioning and behavioral profile in individuals with autism spectrum disorder. Behavioral Sciences, 15(12), 1664. 10.3390/bs15121664 41464007 PMC12730102

[jcv270151-bib-0102] Schott, W. , Tao, S. , & Shea, L. (2022). Co‐occurring conditions and racial‐ethnic disparities: Medicaid enrolled adults on the autism spectrum. Autism Research, 15(1), 70–85. 10.1002/aur.2644 34854249 PMC8812993

[jcv270151-bib-0103] Sedgewick, F. , Leppanen, J. , & Tchanturia, K. (2019). Autistic adult outcomes on weight and body mass index: A large‐scale online study. Eating and Weight Disorders ‐ Studies on Anorexia, Bulimia and Obesity, 25(3), 795–801. 10.1007/s40519-019-00695-8 PMC7256089 31065975

[jcv270151-bib-0104] Shea, L. , Sadowsky, M. , Tao, S. , Rast, J. , Schendel, D. , Chesnokova, A. , & Headen, I. (2024). Perinatal and postpartum health among people with intellectual and developmental disabilities. JAMA Network Open, 7(8), e2428067. 10.1001/jamanetworkopen.2024.28067 39145975 PMC11327882

[jcv270151-bib-0105] Shedlock, K. , Susi, A. , Gorman, G. H. , Hisle‐Gorman, E. , Erdie‐Lalena, C. R. , & Nylund, C. M. (2016). Autism spectrum disorders and metabolic complications of obesity. Journal of Pediatrics, 178, 183–187. 10.1016/j.jpeds.2016.07.055 27592097

[jcv270151-bib-0106] StataCorp . (2023). Stata (release 18). StataCorp LLC.

[jcv270151-bib-0107] Trinh, N. B. , Phan, N. D. T. , Bui, A. T. , Phan, H. T. , Nguyen, L. T. T. , Nguyen, L. H. T. , Do, K. N. , & Dang, A. K. (2023). Nutritional status and eating behavior of children with autism spectrum disorders in Vietnam: A case‐control study. Nutrition and Health, 31(1), 111–119. 10.1177/02601060231152278 36706790

[jcv270151-bib-0108] Tsai, T. , Chao, Y. , Hsieh, C. , & Huang, Y. (2020). Association between atopic dermatitis and autism spectrum disorder: A systematic review and meta‐analysis. Acta Dermato‐Venereologica, 100(10), adv00146. 10.2340/00015555-3501 32399577 PMC9137390

[jcv270151-bib-0109] Tyler, C. V. , Schramm, S. C. , Karafa, M. , Tang, A. S. , & Jain, A. K. (2011). Chronic disease risks in young adults with autism spectrum disorder: Forewarned is forearmed. American Journal on Intellectual and Developmental Disabilities, 116(5), 371–380. 10.1352/1944-7558-116.5.371 21905805

[jcv270151-bib-0110] Voulgarakis, H. , Bendell‐Estroff, D. , & Field, T. (2017). Prevalence of obesity and autism spectrum disorder. Behavioral Development Bulletin, 22(1), 209–214. 10.1037/bdb0000054 PMC5476317 28642808

[jcv270151-bib-0111] Wei, H. , Cheng, Q. , Mei, Q. , Zhang, X. , Chen, L. , Liu, X. , Dai, Y. , Yu, T. , Li, Y. , Zhang, Y. , Chen, J. , Miao, Y. , & Li, T. (2018). Head circumference, body growth and development quotient in autism spectrum disorders are related in chongqing, China. Research in Autism Spectrum Disorders, 54, 83–89. 10.1016/j.rasd.2018.07.003

[jcv270151-bib-0112] Weir, E. , Allison, C. , Ong, K. K. , & Baron‐Cohen, S. (2021). An investigation of the diet, exercise, sleep, BMI, and health outcomes of autistic adults. Molecular Autism, 12(1), 31. 10.1186/s13229-021-00441-x 33964967 PMC8106173

[jcv270151-bib-0113] Wells, G. , Shea, B. , O’Connell, D. , Peterson, je , Welch, V. , Losos, M. , & Tugwell, P. (2011). The Newcastle–Ottawa scale (NOS) for assessing the quality of non‐randomized studies in meta‐analysis. Ottawa Hospital Research Institute. Retrieved from http://www.ohri.ca/programs/clinical_epidemiology/oxford.asp

[jcv270151-bib-0114] Westwood, H. , Eisler, I. , Mandy, W. , Leppanen, J. , Treasure, J. , & Tchanturia, K. (2016). Using the autism‐spectrum quotient to measure autistic traits in anorexia nervosa: A systematic review and meta‐analysis. Journal of Autism and Developmental Disorders, 46(3), 964–977. 10.1007/s10803-015-2641-0 26542816 PMC4746216

[jcv270151-bib-0115] Westwood, H. , & Tchanturia, K. (2017). Autism spectrum disorder in anorexia nervosa: An updated literature review. Current Psychiatry Reports, 19(7), 41. 10.1007/s11920-017-0791-9 28540593 PMC5443871

[jcv270151-bib-0116] Zheng, Z. , Zhang, L. , Li, S. , Zhao, F. , Wang, Y. , Huang, L. , Huang, J. , Zou, R. , Qu, Y. , & Mu, D. (2017). Association among obesity, overweight and autism spectrum disorder: A systematic review and meta‐analysis. Scientific Reports, 7(1), 1–9. 10.1038/s41598-017-12003-4 28916794 PMC5601947

[jcv270151-bib-0117] Zhu, J. , Guo, M. , Yang, T. , Lai, X. , Tang, T. , Chen, J. , Li, L. , & Li, T. (2020). Nutritional status and symptoms in preschool children with autism spectrum disorder: A two‐center comparative study in chongqing and Hainan province, China. Frontiers in Pediatrics, 8, 469. 10.3389/fped.2020.00469 33014918 PMC7494825

[jcv270151-bib-0118] Zimmer, M. H. , Hart, L. C. , Manning‐Courtney, P. , Murray, D. S. , Bing, N. M. , & Summer, S. (2012). Food variety as a predictor of nutritional status among children with autism. Journal of Autism and Developmental Disorders, 42(4), 549–556. 10.1007/s10803-011-1268-z 21556968 PMC8108121

[jcv270151-bib-0119] Zurita, M. F. , Cárdenas, P. A. , Sandoval, M. E. , Peña, M. C. , Fornasini, M. , Flores, N. , Monaco, M. H. , Berding, K. , Donovan, S. M. , Kuntz, T. , Gilbert, J. A. , & Baldeón, M. E. (2020). Analysis of gut microbiome, nutrition and immune status in autism spectrum disorder: A case‐control study in Ecuador. Gut Microbes, 11(3), 453–464. 10.1080/19490976.2019.1662260 31530087 PMC7524316

