# Supplementary material for: Body weight in autism spectrum disorder: A systematic review and meta‐analysis
Source: JCPP Adv. 2026 Aug 4:e70151. Online ahead of print. doi: 10.1002/jcv2.70151 (PMC13438009; doi:10.1002/jcv2.70151)
Supplement: Supplementary file 1 [file JCV2-9999-e70151-s001.docx]

**Body Weight in Autism Spectrum Disorder:**

**A systematic review and meta-analysis**

**Supporting Information**

# Index of contents

[*Table S1 Modified Newcastle-Ottawa scale*	1](#_Toc235283550)

[*Table S2 Included studies*	3](#_Toc235283551)

[*Table S3 Reports not retrieved*	11](#_Toc235283552)

[*Table S4 Excluded studies with reasons*	12](#_Toc235283553)

[*Table S5 Included studies with multiple reports*	51](#_Toc235283554)

[*Table S6 Data issues found in studies during data extraction*	56](#_Toc235283555)

[*Table S7 Additional details on the overall samples of participants*	57](#_Toc235283556)

[*Table S8 Description of the ASD group in studies*	65](#_Toc235283557)

[*Table S9 Description of the Non-ASD group in studies*	73](#_Toc235283558)

[*Table S10 Risk of Bias rating (Newcastle-Ottawa scale)*	82](#_Toc235283559)

[*Table S11 Outcomes: number of individuals per weight category in the group with and without ASD*	84](#_Toc235283560)

[*Table S12 Outcomes: number and prevalence of individuals with obesity in the group with and without ASD*	87](#_Toc235283561)

[*Table S13 Outcomes: number and prevalence of individuals with overweight in the group with and without ASD*	90](#_Toc235283562)

[*Table S14 Outcomes: number and prevalence of individuals with underweight in the group with and without ASD*	93](#_Toc235283563)

[*Figure S1 Risk of obesity in ASD, funnel plot*	96](#_Toc235306771)

[*Figure S2 Risk of obesity in ASD, leave-one-out forest plot*	97](#_Toc235306772)

[*Figure S3 Risk of obesity in ASD, community-based studies only, forest plot*	98](#_Toc235306773)

[*Figure S4 Risk of obesity in ASD, hospital-based studies only, forest plot*	99](#_Toc235306774)

[*Figure S5 Risk of obesity in ASD, quality diagnosis of ASD studies only, forest plot*	100](#_Toc235306775)

[*Figure S6 Risk of obesity in ASD, quality definition of weight studies only, forest plot*	101](#_Toc235306776)

[*Figure S7 Risk of obesity in ASD, eliminating studies with 0 cells only, forest plot*	102](#_Toc235306777)

[*Figure S8 Risk of obesity in ASD, limiting analysis to studies with at least 5 participants per cell, forest plot*	103](#_Toc235306778)

[*Figure S9 Risk of obesity in ASD, Meta-regression with percentage of males in overall sample*	104](#_Toc235306779)

[*Figure S10 Risk of obesity in ASD, Meta-regression with percentage of males in ASD sample*	105](#_Toc235306780)

[*Figure S11 Risk of obesity in ASD, Meta-regression with mean age*	106](#_Toc235306781)

[*Figure S12 Risk of overweight in ASD, funnel plot*	107](#_Toc235306782)

[*Figure S13 Risk of overweight in ASD, leave-one-out forest plot*	108](#_Toc235306783)

[*Figure S14 Risk of overweight in ASD, community-based studies only, forest plot*	109](#_Toc235306784)

[*Figure S15 Risk of overweight in ASD, hospital-based studies only, forest plot*	110](#_Toc235306785)

[*Figure S16 Risk of overweight in ASD, quality diagnosis of ASD studies only, forest plot*	111](#_Toc235306786)

[*Figure S17 Risk of overweight in ASD, quality definition of weight studies only, forest plot*	112](#_Toc235306787)

[*Figure S18 Risk of overweight in ASD, eliminating studies with 0 cells only, forest plot*	113](#_Toc235306788)

[*Figure S19 Risk of overweight in ASD, limiting analysis to studies with at least 5 participants per cell, forest plot*	114](#_Toc235306789)

[*Figure S20 Risk of overweight in ASD, Meta-regression with percentage of males in overall sample*	115](#_Toc235306790)

[*Figure S21 Risk of overweight in ASD, Meta-regression with percentage of males in ASD sample*	116](#_Toc235306791)

[*Figure S22 Risk of overweight in ASD, Meta-regression with mean age*	117](#_Toc235306792)

[*Figure S23 Risk of underweight in ASD, funnel plot*	118](#_Toc235306793)

[*Figure S24 Risk of underweight in ASD, leave-one-out forest plot*	119](#_Toc235306794)

[*Figure S25 Risk of underweight in ASD, community-based studies only, forest plot*	120](#_Toc235306795)

[*Figure S26 Risk of underweight in ASD, hospital-based studies only, forest plot*	121](#_Toc235306796)

[*Figure S27 Risk of underweight in ASD, quality diagnosis of ASD studies only, forest plot*	122](#_Toc235306797)

[*Figure S28 Risk of underweight in ASD, quality definition of weight studies only, forest plot*	123](#_Toc235306798)

[*Figure S29 Risk of underweight in ASD, eliminating studies with 0 cells only, forest plot*	124](#_Toc235306799)

[*Figure S30 Risk of underweight in ASD, limiting analysis to studies with at least 5 participants per cell, forest plot*	125](#_Toc235306800)

[*Figure S31 Risk of underweight in ASD, Meta-regression with percentage of males in overall sample*	126](#_Toc235306801)

[*Figure S32 Risk of underweight in ASD, Meta-regression with percentage of males in ASD sample*	127](#_Toc235306802)

[*Figure S33 Risk of underweight in ASD, Meta-regression with mean age*	128](#_Toc235306803)

*Table S1 Modified Newcastle-Ottawa scale*

Items were modified from the original case-control Newcastle-Ottawa scale (NOS) to be useful in the current project.

**Selection and Representativeness**

**Representativeness of the overall sample**

a) Truly representative of the average in the target population (random sample or whole population)*

b) Somewhat representative of the average in the target population (purposive sampling or evidence that the sample is representative of the source population) *

c) Selected group of users

d) No description of the sampling strategy

**Representativeness of the ASD group**

a) Truly representative of the average group of individuals with ASD in the target population (random sample or whole population)*

b) Somewhat representative of the average group of individuals with ASD in the target population (purposive sampling or evidence that the sample is representative of the source population)*

c) Selected group of users

d) No description of the derivation of the exposed group

**Selection of the non-ASD group**

a) Drawn from the same community as the exposed cohort (those with ASD) *

b) Drawn from a different source

c) No description of the derivation of the non-exposed cohort

**Ascertainment of exposure (ASD)**

a) Clinical Diagnosis/ADI-R ADOS*

b) Administrative registry *

c) Other (Self report, questionnaire, scale)

**Comparability**

**Comparability of cohorts (those with ASD and without) based on the design or analysis**

a) study controls for age and gender (matching or p>0.1 or analysis separated by gender) *
b) study controls for any additional factor *

**Outcome**

**Sample size**

a) Adequately powered to detect a difference (at least 5 events per cell of the confusion matrix) *

b) Not justified

**Ascertainment of outcome (weight category)**

a) Clinical record *

b) Measurement *

c) Self/other report

d) No description/other

**Clear definition of obesity**

a) PC 95/Z2/Mention to Who*

b) Other

**Clear definition of overweight**

a) PC 85/Z1.5/Mention to Who*

b) Other

**Clear definition of underweight**

a) PC 5/Z2/Mention to Who*

b) Other

Table *S2* Included studies

| **Author year** | **Reference** |
| --- | --- |
| Ahlberg et al., 2022 | Ahlberg, R., Garcia‐Argibay, M., Hirvikoski, T., Boman, M., Chen, Q., Taylor, M. J., Frans, E., Bölte, S., & Larsson, H. (2022). Shared familial risk factors between autism spectrum disorder and obesity – a register‐based familial coaggregation cohort study. *Journal of Child Psychology and Psychiatry*, *63*(8), 890‑899. <https://doi.org/10.1111/jcpp.13538> |
| Alkhalidy et al., 2021 | Alkhalidy, H., Abushaikha, A., Alnaser, K., Obeidat, M. D., & Al-Shami, I. (2021). Nutritional Status of Pre-school Children and Determinant Factors of Autism : A Case-Control Study. *Frontiers in Nutrition*, *8*, 627011. https://doi.org/10.3389/fnut.2021.627011 |
| Al-Kindi et al., 2016 | Al-Kindi, N. M., Al-Farsi, Y. M., Waly, M. I., A-Shafaee, M. S., & Bakheit, C. S. (2016). *Dietary Intake and Food Preferences of Autistic Children Versus Children with Typical Development : A Comparative Cross-Sectional Study*. *6*, 72‑85. |
| Ames et al., 2022 | Ames, J. L., Morgan, E. H., Giwa Onaiwu, M., Qian, Y., Massolo, M. L., & Croen, L. A. (2022). Racial/Ethnic Differences in Psychiatric and Medical Diagnoses Among Autistic Adults. *Autism in Adulthood*, *4*(4), 290‑305. https://doi.org/10.1089/aut.2021.0083 |
| Amin et al., 2022 | Amin, M., Faiyaz, S. I., Butt, M. S., Javed, A., Saleem, J., & Saeed, A. (2022). Food preferences and eating behavior among children with autism spectrum disorder : A causal-comparativestudy in Lahore. *Avicenna*, *2022*(2). https://doi.org/10.5339/avi.2022.9 |
| Arija et al., 2023 | Arija, V., Esteban-Figuerola, P., Morales-Hidalgo, P., Jardí, C., & Canals-Sans, J. (2023). Nutrient intake and adequacy in children with autism spectrum disorder : EPINED epidemiological study. *Autism*, *27*(2), 371‑388. https://doi.org/10.1177/13623613221098237 |
| Bandini et al., 2013 | Bandini, L. G., Gleason, J., Curtin, C., Lividini, K., Anderson, S. E., Cermak, S. A., Maslin, M., & Must, A. (2013). Comparison of physical activity between children with autism spectrum disorders and typically developing children. *Autism*, *17*(1), 44‑54. https://doi.org/10.1177/1362361312437416 |
| Barnhill et al., 2017 | Barnhill, K., Gutierrez, A., Ghossainy, M., Marediya, Z., Marti, C. N., & Hewitson, L. (2017). Growth status of children with autism spectrum disorder : A case–control study. *Journal of Human Nutrition and Dietetics*, *30*(1), 59‑65. https://doi.org/10.1111/jhn.12396 |
| Bener et al., 2014 | Bener, A., Khattab, A., & Al-Dabbagh, M. (2014). Is high prevalence of Vitamin D deficiency evidence for autism disorder? : In a highly endogamous population. *Journal of Pediatric Neurosciences*, *9*(3), 227. https://doi.org/10.4103/1817-1745.147574 |
| Błażewicz et al., 2020 | Błażewicz, A., Szymańska, I., Dolliver, W., Suchocki, P., Turło, J., Makarewicz, A., & Skórzyńska-Dziduszko, K. (2020). Are Obese Patients with Autism Spectrum Disorder More Likely to Be Selenium Deficient? Research Findings on Pre- and Post-Pubertal Children. *Nutrients*, *12*(11), 3581. https://doi.org/10.3390/nu12113581 |
| Broder-Fingert et al., 2014 | Broder-Fingert, S., Brazauskas, K., Lindgren, K., Iannuzzi, D., & Van Cleave, J. (2014). Prevalence of Overweight and Obesity in a Large Clinical Sample of Children With Autism. *Academic Pediatrics*, *14*(4), 408‑414. https://doi.org/10.1016/j.acap.2014.04.004 |
| Buro et al., 2022 | Buro, A. W., Salinas-Miranda, A., Marshall, J., Gray, H. L., & Kirby, R. S. (2022). Correlates of obesity in adolescents with and without autism spectrum disorder : The 2017–2018 National Survey of Children’s Health. *Disability and Health Journal*, *15*(2), 101221. https://doi.org/10.1016/j.dhjo.2021.101221 |
| Castro et al., 2016 | Castro, K., Faccioli, L. S., Baronio, D., Gottfried, C., Perry, I. S., & Riesgo, R. (2016). Feeding behavior and dietary intake of male children and adolescents with autism spectrum disorder : A case-control study. *International Journal of Developmental Neuroscience*, *53*, 68‑74. https://doi.org/10.1016/j.ijdevneu.2016.07.003 |
| Chen et al., 2016 | Chen, M.-H., Lan, W.-H., Hsu, J.-W., Huang, K.-L., Su, T.-P., Li, C.-T., Lin, W.-C., Tsai, C.-F., Tsai, S.-J., Lee, Y.-C., Chen, Y.-S., Pan, T.-L., Chang, W.-H., Chen, T.-J., & Bai, Y.-M. (2016). Risk of Developing Type 2 Diabetes in Adolescents and Young Adults With Autism Spectrum Disorder : A Nationwide Longitudinal Study. *Diabetes Care*, *39*(5), 788‑793. https://doi.org/10.2337/dc15-1807 |
| Coppola et al., 2024 | Coppola, S., Nocerino, R., Oglio, F., Golia, P., Falco, M. C., Riccio, M. P., Carucci, L., Rea, T., Simeone, S., Garotti, R., Marani, N., Bravaccio, C., & Canani, R. B. (2024). Adverse food reactions and alterations in nutritional status in children with autism spectrum disorders : Results of the NAFRA project. *Italian Journal of Pediatrics*, *50*(1), 228. https://doi.org/10.1186/s13052-024-01794-8 |
| Corbett et al., 2021 | Corbett, B. A., Muscatello, R. A., Horrocks, B. K., Klemencic, M. E., & Tanguturi, Y. (2021). Differences in Body Mass Index (BMI) in Early Adolescents with Autism Spectrum Disorder Compared to Youth with Typical Development. *Journal of Autism and Developmental Disorders*, *51*(8), 2790‑2799. https://doi.org/10.1007/s10803-020-04749-0 |
| Criado et al., 2018 | Criado, K. K., Sharp, W. G., McCracken, C. E., De Vinck-Baroody, O., Dong, L., Aman, M. G., McDougle, C. J., McCracken, J. T., Eugene Arnold, L., Weitzman, C., Leventhal, J. M., Vitiello, B., & Scahill, L. (2018). Overweight and obese status in children with autism spectrum disorder and disruptive behavior. *Autism*, *22*(4), 450‑459. https://doi.org/10.1177/1362361316683888 |
| Curtin et al., 2010 | Curtin, C., Anderson, S. E., Must, A., & Bandini, L. (2010). The prevalence of obesity in children with autism : A secondary data analysis using nationally representative data from the National Survey of Children’s Health. *BMC Pediatrics*, *10*(1), 11. https://doi.org/10.1186/1471-2431-10-11 |
| Davignon et al., 2018 | Davignon, M. N., Qian, Y., Massolo, M., & Croen, L. A. (2018). Psychiatric and Medical Conditions in Transition-Aged Individuals With ASD. *Pediatrics*, *141*(Supplement_4), S335‑S345. https://doi.org/10.1542/peds.2016-4300K |
| Eliasziw et al., 2021 | Eliasziw, M., Kral, T. V. E., Segal, M., Sikich, L., Phillips, S., Tybor, D. J., Bandini, L. G., Curtin, C., & Must, A. (2021). Healthy-Weight Kindergarten Children with Autism Spectrum Disorder May Become Overweight and Obese during the First Few Years of Elementary School. *The Journal of Pediatrics: X*, *7*, 100074. https://doi.org/10.1016/j.ympdx.2021.100074 |
| Esposito et al., 2019 | Esposito, M., Sloan, J., Nappo, R., Fadda, R., Fotia, F., Napoli, E., Mazzone, L., Valeri, G., & Vicari, S. (2019). *Sensory Processing, Gastrointestinal Symptoms and Parental Feeding Practices in The Explanation of Food Selectivity : Clustering Children with and Without Autism*. *120*. |
| Esteban-Figuerola et al., 2021 | Esteban-Figuerola, P., Morales-Hidalgo, P., Arija-Val, V., & Canals-Sans, J. (2021). Are there anthropometric and body composition differences between children with autism spectrum disorder and children with typical development ? Analysis by age and spectrum severity in a school population. *Autism*, *25*(5), 1307‑1320. https://doi.org/10.1177/1362361320987724 |
| Flygare Wallén et al., 2018 | Flygare Wallén, E., Ljunggren, G., Carlsson, A. C., Pettersson, D., & Wändell, P. (2018). High prevalence of diabetes mellitus, hypertension and obesity among persons with a recorded diagnosis of intellectual disability or autism spectrum disorder. *Journal of Intellectual Disability Research*, *62*(4), 269‑280. https://doi.org/10.1111/jir.12462 |
| Fuentes-Albero et al., 2024 | Fuentes-Albero, M., Mafla-España, M. A., Martínez-Raga, J., & Cauli, O. (2024). Autistic Children/Adolescents Have Lower Adherence to the Mediterranean Diet and Higher Salivary IL-6 Concentration : Potential Diet–Inflammation Links? *Pathophysiology*, *31*(3), 376‑387. https://doi.org/10.3390/pathophysiology31030028 |
| Garcia & Odahowski, 2023 | Garcia, J. M., & Odahowski, C. L. (2023). An urban versus rural comparison of obesity between youth with and without autism spectrum disorder. *Autism Research*, *16*(1), 200‑207. https://doi.org/10.1002/aur.2856 |
| Hand et al., 2020 | Hand, B. N., Angell, A. M., Harris, L., & Carpenter, L. A. (2020). Prevalence of physical and mental health conditions in Medicare-enrolled, autistic older adults. *Autism*, *24*(3), 755‑764. https://doi.org/10.1177/1362361319890793 |
| Healy et al., 2017 | Healy, S., Haegele, J. A., Grenier, M., & Garcia, J. M. (2017). Physical Activity, Screen-Time Behavior, and Obesity Among 13-Year Olds in Ireland with and without Autism Spectrum Disorder. *Journal of Autism and Developmental Disorders*, *47*(1), 49‑57. https://doi.org/10.1007/s10803-016-2920-4 |
| Herndon et al., 2009 | Herndon, A. C., DiGuiseppi, C., Johnson, S. L., Leiferman, J., & Reynolds, A. (2009). Does Nutritional Intake Differ Between Children with Autism Spectrum Disorders and Children with Typical Development? *Journal of Autism and Developmental Disorders*, *39*(2), 212‑222. https://doi.org/10.1007/s10803-008-0606-2 |
| Hill et al., 2015 | Hill, A. P., Zuckerman, K. E., & Fombonne, E. (2015). Obesity and Autism. *PEDIATRICS*, *136*(6), 1051‑1061. https://doi.org/10.1542/peds.2015-1437 |
| Hyman et al., 2012 | Hyman, S. L., Stewart, P. A., Schmidt, B., Cain, U., Lemcke, N., Foley, J. T., Peck, R., Clemons, T., Reynolds, A., Johnson, C., Handen, B., James, S. J., Courtney, P. M., Molloy, C., & Ng, P. K. (2012). Nutrient Intake From Food in Children With Autism. *Pediatrics*, *130*(Supplement_2), S145‑S153. https://doi.org/10.1542/peds.2012-0900L |
| Islam et al., 2024 | Islam, M. N., Chowdhury, S. R., Kabir, H., Bari, F. S., & Hossain, A. (2024). Dietary diversity and nutritional status of children with and without autism spectrum disorder : A comparative cross-sectional study in Bangladesh. *Discover Social Science and Health*, *4*(1), 76. https://doi.org/10.1007/s44155-024-00140-x |
| Kerekes et al., 2015 | Kerekes, N., Tajnia, A., Lichtenstein, P., Lundström, S., Anckarsäter, H., Nilsson, T., & Råstam, M. (2015). Neurodevelopmental problems and extremes in BMI. *PeerJ*, *3*, e1024. https://doi.org/10.7717/peerj.1024 |
| Kral et al., 2015 | Kral, T. V. E., Souders, M. C., Tompkins, V. H., Remiker, A. M., Eriksen, W. T., & Pinto-Martin, J. A. (2015). Child Eating Behaviors and Caregiver Feeding Practices in Children with Autism Spectrum Disorders. *Public Health Nursing*, *32*(5), 488‑497. https://doi.org/10.1111/phn.12146 |
| Kummer et al., 2016 | Kummer, A., Barbosa, I. G., Rodrigues, D. H., Rocha, N. P., Da Silva Rafael, M., Pfeilsticker, L., Simõese Silva, A. C., & Teixeira, A. L. (2016). Frequency of overweight and obesity in children and adolescents with autism and attention deficit/hyperactivity disorder. *Revista Paulista de Pediatria*, *34*(1), 71‑77. https://doi.org/10.1016/j.rppede.2015.12.006 |
| Levy et al., 2019 | Levy, S. E., Pinto-Martin, J. A., Bradley, C. B., Chittams, J., Johnson, S. L., Pandey, J., Pomykacz, A., Ramirez, A., Reynolds, A., Rubenstein, E., Schieve, L. A., Shapira, S. K., Thompson, A., Young, L., & Kral, T. V. E. (2019). Relationship of Weight Outcomes, Co-Occurring Conditions, and Severity of Autism Spectrum Disorder in the Study to Explore Early Development. *The Journal of Pediatrics*, *205*, 202‑209. https://doi.org/10.1016/j.jpeds.2018.09.003 |
| Liu et al., 2016 | Liu, X., Liu, J., Xiong, X., Yang, T., Hou, N., Liang, X., Chen, J., Cheng, Q., & Li, T. (2016). Correlation between Nutrition and Symptoms : Nutritional Survey of Children with Autism Spectrum Disorder in Chongqing, China. *Nutrients*, *8*(5), 294‑294. https://doi.org/10.3390/nu8050294 |
| M. Metwally et al., 2024 | M. Metwally, A., A. Helmy, M., Aboulghate, A., Abu-Mandil Hassan, N., S. Mahmoud, W., S. Ismail, A., M. El Shebini, S., H. Ahmed, N., B. Mabrok, H., H. Mahmoud, M., A. Elshaarawy, G., Elsaied, A., A. Ashaat, E., S. ElRifay, A., Abdelhady, S., E. Eldeeb, S., M. El-Saied, M., A. El-Masry, S., E Hassan, N., … M. Alian, K. (2024). The odds of having obesity in Egyptian children with autism spectrum disorders is higher than stunting compared to healthy developing peers : A national survey. *BMC Pediatrics*, *24*(1), 465. https://doi.org/10.1186/s12887-024-04934-5 |
| Marí-Bauset et al., 2015 | Marí-Bauset, S., Llopis-González, A., Zazpe, I., Mari-Sanchis, A., & Morales-Suárez-Varela, M. (2015). Anthropometric measures of Spanish children with autism spectrum disorder. *Research in Autism Spectrum Disorders*, *9*, 26‑33. https://doi.org/10.1016/j.rasd.2014.09.013 |
| Mathew et al., 2022 | Mathew, N. E., Mallitt, K., Masi, A., Katz, T., Walker, A. K., Morris, M. J., & Ooi, C. Y. (2022). Dietary intake in children on the autism spectrum is altered and linked to differences in autistic traits and sensory processing styles. *Autism Research*, *15*(10), 1824‑1839. https://doi.org/10.1002/aur.2798 |
| Mayes et al., 2024 | Mayes, S. D., Calhoun, S. L., Kallus, R., Baweja, R., & Waschbusch, D. A. (2024). Cognitive Disengagement Syndrome (Formerly Sluggish Cognitive Tempo) and Comorbid Symptoms in Child Autism, ADHD, and Elementary School Samples. *Journal of Psychopathology and Behavioral Assessment*, *46*(3), 857‑865. https://doi.org/10.1007/s10862-024-10145-0 |
| McCoy et al., 2016 | McCoy, S. M., Jakicic, J. M., & Gibbs, B. B. (2016). Comparison of Obesity, Physical Activity, and Sedentary Behaviors Between Adolescents With Autism Spectrum Disorders and Without. *Journal of Autism and Developmental Disorders*, *46*(7), 2317‑2326. https://doi.org/10.1007/s10803-016-2762-0 |
| McCoy & Morgan, 2020 | McCoy, S. M., & Morgan, K. (2020). Obesity, physical activity, and sedentary behaviors in adolescents with autism spectrum disorder compared with typically developing peers. *Autism*, *24*(2), 387‑399. https://doi.org/10.1177/1362361319861579 |
| Mehra et al., 2024 | Mehra, S., Salinas-Miranda, A. A., Buro, A. W., Marshall, J., & Kirby, R. S. (2024). The role of adverse childhood experiences in obesity among adolescents with autism spectrum disorder : National survey of Children’s health 2018-2019. *Disability and Health Journal*, *17*(2), 101550. https://doi.org/10.1016/j.dhjo.2023.101550 |
| Mendive Dubourdieu & Guerendiain, 2022 | Mendive Dubourdieu, P., & Guerendiain, M. (2022). Dietary Intake, Nutritional Status and Sensory Profile in Children with Autism Spectrum Disorder and Typical Development. *Nutrients*, *14*(10), 2155. https://doi.org/10.3390/nu14102155 |
| Mills et al., 2007 | Mills, J. L., Hediger, M. L., Molloy, C. A., Chrousos, G. P., Manning-Courtney, P., Yu, K. F., Brasington, M., & England, L. J. (2007). Elevated levels of growth-related hormones in autism and autism spectrum disorder. *Clinical Endocrinology*, *67*(2), 230‑237. https://doi.org/10.1111/j.1365-2265.2007.02868.x |
| Molina‐López et al., 2021 | Molina‐López, J., Leiva‐García, B., Planells, E., & Planells, P. (2021). Food selectivity, nutritional inadequacies, and mealtime behavioral problems in children with autism spectrum disorder compared to neurotypical children. *International Journal of Eating Disorders*, *54*(12), 2155‑2166. https://doi.org/10.1002/eat.23631 |
| Must, Eliasziw, Bowling, et al., 2023 | Must, A., Eliasziw, M., Bowling, A., Magaña, S., Stanish, H., Bandini, L., Curtin, C., & Kral, T. V. E. (2023). Relationship Between Childhood Obesity and Autism Spectrum Disorder Varies by Child’s Age and Parents’ Weight Status in a Sample of Sibling Dyads. *Childhood Obesity*, *19*(5), 309‑315. https://doi.org/10.1089/chi.2022.0064 |
| Must, Eliasziw, Stanish, et al., 2023 | Must, A., Eliasziw, M., Stanish, H., Curtin, C., Bandini, L. G., & Bowling, A. (2023). Passive and social screen time in children with autism and in association with obesity. *Frontiers in Pediatrics*, *11*, 1198033. https://doi.org/10.3389/fped.2023.1198033 |
| Phillips et al., 2014 | Phillips, K. L., Schieve, L. A., Visser, S., Boulet, S., Sharma, A. J., Kogan, M. D., Boyle, C. A., & Yeargin-Allsopp, M. (2014). Prevalence and Impact of Unhealthy Weight in a National Sample of US Adolescents with Autism and Other Learning and Behavioral Disabilities. *Maternal and Child Health Journal*, *18*(8), 1964‑1975. https://doi.org/10.1007/s10995-014-1442-y |
| Raspini et al., 2021 | Raspini, B., Prosperi, M., Guiducci, L., Santocchi, E., Tancredi, R., Calderoni, S., Morales, M. A., Morelli, M., Simione, M., Fiechtner, L., Muratori, F., & Cena, H. (2021). Dietary Patterns and Weight Status in Italian Preschoolers with Autism Spectrum Disorder and Typically Developing Children. *Nutrients*, *13*(11), 4039. https://doi.org/10.3390/nu13114039 |
| Riccio et al., 2020 | Riccio, M. P., Catone, G., Siracusano, R., Occhiati, L., Bernardo, P., Sarnataro, E., Corrado, G., Bravaccio, C. (2020). Vitamin D deficiency is not related to eating habits in children with Autistic Spectrum Disorder. *AIMS Public Health*, *7*(4), 792‑803. https://doi.org/10.3934/publichealth.2020061 |
| Rimmer et al., 2010 | Rimmer, J. H., Yamaki, K., Lowry, D. M. D., Wang, E., & Vogel, L. C. (2010). Obesity and obesity-related secondary conditions in adolescents with intellectual/developmental disabilities. *Journal of Intellectual Disability Research*, *54*(9), 787‑794. https://doi.org/10.1111/j.1365-2788.2010.01305.x |
| Rouphael et al., 2024 | Rouphael, M., Sacre, Y., Bitar, T., Andres, C. R., & Hleihel, W. (2024). Body Composition and Anthropometric Measurements in Children and Adolescents with Autism Spectrum Disorder : A Case–Control Study in Lebanon. *Nutrients*, *16*(6), 847. https://doi.org/10.3390/nu16060847 |
| Schott et al., 2022 | Schott, W., Tao, S., & Shea, L. (2022). Co‐occurring conditions and racial‐ethnic disparities : Medicaid enrolled adults on the autism spectrum. *Autism Research*, *15*(1), 70‑85. https://doi.org/10.1002/aur.2644 |
| Sedgewick et al., 2019 | Sedgewick, F., Leppanen, J., & Tchanturia, K. (2019). Autistic adult outcomes on weight and body mass index : A large-scale online study. *Eating and Weight Disorders - Studies on Anorexia, Bulimia and Obesity*, *25*(3), 795‑801. https://doi.org/10.1007/s40519-019-00695-8 |
| Shea et al., 2024 | Shea, L., Sadowsky, M., Tao, S., Rast, J., Schendel, D., Chesnokova, A., & Headen, I. (2024). Perinatal and Postpartum Health Among People With Intellectual and Developmental Disabilities. *JAMA Network Open*, *7*(8), e2428067. https://doi.org/10.1001/jamanetworkopen.2024.28067 |
| Shedlock et al., 2016 | Shedlock, K., Susi, A., Gorman, G. H., Hisle-Gorman, E., Erdie-Lalena, C. R., & Nylund, C. M. (2016). Autism Spectrum Disorders and Metabolic Complications of Obesity. *Journal of Pediatrics*, *178*, 183-187.e1. https://doi.org/10.1016/j.jpeds.2016.07.055 |
| Trinh et al., 2023 | Trinh, N. B., Phan, N. D. T., Bui, A. T., Phan, H. T., Nguyen, L. T. T., Nguyen, L. H. T., Do, K. N., & Dang, A. K. (2023). Nutritional status and eating behavior of children with autism spectrum disorders in Vietnam : A case-control study. *Nutrition and Health*, *31*(1), 111‑119. https://doi.org/10.1177/02601060231152278 |
| Tyler et al., 2011 | Tyler, C. V., Schramm, S. C., Karafa, M., Tang, A. S., & Jain, A. K. (2011). Chronic Disease Risks in Young Adults With Autism Spectrum Disorder : Forewarned Is Forearmed. *American Journal on Intellectual and Developmental Disabilities*, *116*(5), 371‑380. https://doi.org/10.1352/1944-7558-116.5.371 |
| Wei et al., 2018 | Wei, H., Cheng, Q., Mei, Q., Zhang, X., Chen, L., Liu, X., Dai, Y., Yu, T., Li, Y., Zhang, Y., Chen, J., Miao, Y., & Li, T. (2018). Head circumference, body growth and development quotient in autism spectrum disorders are related in Chongqing, China. *Research in Autism Spectrum Disorders*, *54*, 83‑89. https://doi.org/10.1016/j.rasd.2018.07.003 |
| Weir et al., 2021 | Weir, E., Allison, C., Ong, K. K., & Baron-Cohen, S. (2021). An investigation of the diet, exercise, sleep, BMI, and health outcomes of autistic adults. *Molecular Autism*, *12*(1), 31. https://doi.org/10.1186/s13229-021-00441-x |
| Zhu et al., 2020 | Zhu, J., Guo, M., Yang, T., Lai, X., Tang, T., Chen, J., Li, L., & Li, T. (2020). Nutritional Status and Symptoms in Preschool Children With Autism Spectrum Disorder : A Two-Center Comparative Study in Chongqing and Hainan Province, China. *Frontiers in Pediatrics*, *8*, 469. https://doi.org/10.3389/fped.2020.00469 |
| Zimmer et al., 2012 | Zimmer, M. H., Hart, L. C., Manning-Courtney, P., Murray, D. S., Bing, N. M., & Summer, S. (2012). Food Variety as a Predictor of Nutritional Status Among Children with Autism. *Journal of Autism and Developmental Disorders*, *42*(4), 549‑556. https://doi.org/10.1007/s10803-011-1268-z |
| Zurita et al., 2020 | Zurita, M. F., Cárdenas, P. A., Sandoval, M. E., Peña, M. C., Fornasini, M., Flores, N., Monaco, M. H., Berding, K., Donovan, S. M., Kuntz, T., Gilbert, J. A., & Baldeón, M. E. (2020). Analysis of gut microbiome, nutrition and immune status in autism spectrum disorder : A case-control study in Ecuador. *Gut Microbes*, *11*(3), 453‑464. https://doi.org/10.1080/19490976.2019.1662260 |

Table S3 Reports not retrieved

Studies for which we were not able to locate the full text after 1-Searching our institutional databases, 2-Searching online for access (including the journal website), 3-asking for an institutional inter-library loan.

**Reference**

Aghaeinejad, M; Djafarian, K; Mahmoudi, M; Maskooni, M. D. (2013) Comparison of energy and macronutrients intake between children with autism and healthy children. *International Research Journal of Applied and Basic Sciences*, 5(6),667-670

Table S4 Excluded studies with reasons

| **id** | **Title** | **Journal** | **Volume** | **Issue** | **Pages** | **DOI** | **Reason for exclusion** |
| --- | --- | --- | --- | --- | --- | --- | --- |
| [Anonymous] 2013 | Girls with anorexia nervosa have increased number of autistic traits | British Journal of Hospital Medicine | 74 | 9 | 490-490 |  | No frequency of weight categories |
| Alfaris 2024 | Nutritional Status and Dietary Behaviors of Children with Intellectual or Developmental Disabilities in Saudi Arabia: A Systematic Review | Journal of Multidisciplinary Healthcare | 17 |  | 3371-3399 | 10.2147/JMDH.S473107 | Other systematic review or meta-analysis |
| Alqoaer 2024 | Gastrointestinal Symptoms in Autism Spectrum Disorder: A Case-Control Study. | Cureus | 16 | 8 | e68292 | 10.7759/cureus.68292 | Wrong comparator |
| Anderson 2021 | Psychiatric disorders in individuals born very preterm / very low-birth weight: An individual participant data (IPD) meta-analysis. | EClinicalMedicine | 42 |  | 101216 | 10.1016/j.eclinm.2021.101216 | Other systematic review or meta-analysis |
| Aran 2019 | Lower circulating endocannabinoid levels in children with autism spectrum disorder. | Mol Autism | 10 |  | 2 | 10.1186/s13229-019-0256-6 | No frequency of weight categories |
| Arkesteyn 2022 | Physical activity correlates in children and adolescents with autism spectrum disorder: a systematic review. | Disabil Rehabil | 44 | 22 | 6539-6550 | 10.1080/09638288.2021.1970251 | Other systematic review or meta-analysis |
| Arrondo 2022 | Associations between mental and physical conditions in children and adolescents: An umbrella review. | Neurosci Biobehav Rev | 137 |  | 104662 | 10.1016/j.neubiorev.2022.104662 | Meta-analysis of interest |
| Arslan 2022 | Effects of a 12-week structured circuit exercise program on physical fitness levels of children with autism spectrum condition and typically developing children. | Int J Dev Disabil | 68 | 4 | 500-510 | 10.1080/20473869.2020.1819943 | No frequency of weight categories |
| Arslan 2024 | The relationship between nutritional problems, eating behaviors, and diet quality in children with autism | International Journal of Developmental Disabilities |  |  |  | 10.1080/20473869.2024.2396692 | Wrong study design |
| Avila-Parcet 2020 | Autism spectrum traits in patients with eating disorder | European Neuropsychopharmacology | 40 |  | S91-S92 | 10.1016/j.euroneuro.2020.09.122 | Inadequate definition of ASD |
| Babb 2022 | A comparison of the eating disorder service experiences of autistic and non-autistic women in the UK. | Eur Eat Disord Rev | 30 | 5 | 616-627 | 10.1002/erv.2930 | Wrong study design |
| Bala 2016 | Plasma amino acid profile in autism spectrum disorder (ASD). | Eur Rev Med Pharmacol Sci | 20 | 5 | 923-9 |  | No frequency of weight categories |
| Bandini 2008 | Dietary Risk Factors for Overweight among Children with Autism Spectrum Disorders and Typically Developing Children | Obesity | 16 |  | S201-S201 |  | Wrong study design |
| Bandini 2013 | Food as a reward and weight status in children with autism | Faseb Journal | 27 |  |  |  | Wrong study design |
| Baraskewich 2021 | Feeding and eating problems in children and adolescents with autism: A scoping review. | Autism | 25 | 6 | 1505-1519 | 10.1177/1362361321995631 | Wrong study design |
| Baron-Cohen 2013 | Do girls with anorexia nervosa have elevated autistic traits? | Mol Autism | 4 | 1 | 24 | 10.1186/2040-2392-4-24 | Inadequate definition of ASD |
| Bauer 2015 | Body Mass Index and Parent-Identified Eating Concerns in Children With Autism Spectrum Disorders | Journal of Developmental and Behavioral Pediatrics | 36 | 2 | S8-S8 |  | Wrong study design |
| Bennett 2021 | Running Biomechanics of Adolescents With Autism Spectrum Disorder. | J Biomech Eng | 143 | 11 |  | 10.1115/1.4051346 | Wrong comparator |
| Bentz 2022 | Case series of family-based treatment for restrictive-type eating disorders and comorbid autism: What can we learn? A brief report. | Eur Eat Disord Rev | 30 | 5 | 641-647 | 10.1002/erv.2938 | Wrong comparator |
| Berkman 2007 | Outcomes of eating disorders: a systematic review of the literature. | Int J Eat Disord | 40 | 4 | 293-309 | 10.1002/eat.20369 | Other systematic review or meta-analysis |
| Bertelli 2025 | Eating and feeding disorders in adults with intellectual developmental disorder with and without autism spectrum disorder. | J Intellect Disabil Res | 69 | 2 | 153-164 | 10.1111/jir.13195 | Wrong comparator |
| Birnbaum 2022 | Clinical Characterization of Copy Number Variants Associated With Neurodevelopmental Disorders in a Large-scale Multiancestry Biobank. | JAMA Psychiatry | 79 | 3 | 250-259 | 10.1001/jamapsychiatry.2021.4080 | Inadequate definition of ASD |
| Björnsdotter 2017 | Grey matter correlates of autistic traits in women with anorexia nervosa. | J Psychiatry Neurosci | 43 | 1 | 1-8 | 10.1503/jpn.170072 | Inadequate definition of ASD |
| Błażewicz 2016 | Iodine in autism spectrum disorders. | J Trace Elem Med Biol | 34 |  | 32-7 | 10.1016/j.jtemb.2015.12.002 | No frequency of weight categories |
| Błażewicz 2020 | Assessment of Changes over Time of Lipid Profile, C-Reactive Protein Level and Body Mass Index in Teenagers and Young Adults on Different Diets Belonging to Autism Spectrum Disorder. | Nutrients | 12 | 9 |  | 10.3390/nu12092594 | Wrong study design |
| Bokkala 2008 | Correlates of periodic limb movements of sleep in the pediatric population. | Pediatr Neurol | 39 | 1 | 33-9 | 10.1016/j.pediatrneurol.2008.03.008 | Other systematic review or meta-analysis |
| Boltri 2021 | Anorexia Nervosa and Autism Spectrum Disorder: A Systematic Review. | Psychiatry Res | 306 |  | 114271 | 10.1016/j.psychres.2021.114271 | Other systematic review or meta-analysis |
| Boyd 2023 | Hypovitaminosis D in persons with Down syndrome and autism spectrum disorder. | J Neurodev Disord | 15 | 1 | 35 | 10.1186/s11689-023-09503-y | No frequency of weight categories |
| Brede 2024 | The clinical characteristics of autistic women with restrictive eating disorders. | BJPsych Open | 10 | 4 | e131 | 10.1192/bjo.2024.65 | Wrong comparator |
| Buro 2022 | Obesity and co-occurring conditions among adolescents with autism spectrum disorder: The National Survey of Children's Health 2017-2018 | Research in Autism Spectrum Disorders | 92 |  |  | 10.1016/j.rasd.2022.101927 | No frequency of weight categories |
| Çag 2021 | Evaluation of children with disabilities: BMI indicators and difficulties experienced by parents Evaluation of disabled children | Annals of Clinical and Analytical Medicine | 12 | 9 | 969-974 | 10.4328/ACAM.20543 | Wrong comparator |
| CalisanKinter 2024 | The Sensory Profiles, Eating Behaviors, and Quality of Life of Children with Autism Spectrum Disorder and Avoidant/Restrictive Food Intake Disorder. | Psychiatr Q | 95 | 1 | 85-106 | 10.1007/s11126-023-10063-6 | Wrong comparator |
| Canals-Sans 2022 | Do Children with Autism Spectrum Disorders Eat Differently and Less Adequately than Those with Subclinical ASD and Typical Development? EPINED Epidemiological Study. | J Autism Dev Disord | 52 | 1 | 361-375 | 10.1007/s10803-021-04928-7 | No frequency of weight categories |
| Carpita 2022 | Investigating the relationship between orthorexia nervosa and autistic traits in a university population. | CNS Spectr | 27 | 5 | 613-620 | 10.1017/S1092852921000420 | Wrong comparator |
| Carton 2014 | Assessing the relationship between eating disorder psychopathology and autistic traits in a non-clinical adult population. | Eat Weight Disord | 19 | 3 | 285-93 | 10.1007/s40519-013-0086-z | Wrong comparator |
| Chapek 2025 | The Prevalence of Persistent Toe Walking in Children With and Without Autism Spectrum Disorder and the Odds of Subsequent Surgery. | J Foot Ankle Surg | 64 | 1 | 16-20 | 10.1053/j.jfas.2024.08.005 | Wrong comparator |
| Cherif 2018 | Feeding Problems in Children with Autism Spectrum Disorders | Journal of Family Medicine | 1 | 1 | 30-39 | 10.14302/issn.2640-690X.jfm-18-2252 | No frequency of weight categories |
| Chieh 2021 | Systematic review investigating the relationship between autism spectrum disorder and metabolic dysfunction. | Res Autism Spectr Disord | 86 |  |  | 10.1016/j.rasd.2021.101821 | Other systematic review or meta-analysis |
| Coffey 2021 | Comparison of fitness levels between elementary school children with autism spectrum disorder and age-matched neurotypically developing children. | Autism Res | 14 | 9 | 2038-2046 | 10.1002/aur.2559 | No frequency of weight categories |
| Coombs 2011 | An investigation into the relationship between eating disorder psychopathology and autistic symptomatology in a non-clinical sample. | Br J Clin Psychol | 50 | 3 | 326-38 | 10.1348/014466510X524408 | Wrong comparator |
| Corbett 2020 | Pubertal Timing During Early Adolescence: Advanced Pubertal Onset in Females with Autism Spectrum Disorder. | Autism Res | 13 | 12 | 2202-2215 | 10.1002/aur.2406 | No frequency of weight categories |
| Corbett 2022 | Examination of pubertal timing and tempo in females and males with autism spectrum disorder compared to typically developing youth. | Autism Res | 15 | 10 | 1894-1908 | 10.1002/aur.2786 | No frequency of weight categories |
| Cornish 1998 | A balanced approach towards healthy eating in autism | Journal of Human Nutrition and Dietetics | 11 | 6 | 501-509 | 10.1046/j.1365-277X.1998.00132.x | Wrong comparator |
| Cortese 2021 | Term Birth Weight and Neurodevelopmental Outcomes. | Epidemiology | 32 | 4 | 583-590 | 10.1097/EDE.0000000000001350 | Perinatal weight |
| Courty 2013 | Levels of autistic traits in anorexia nervosa: a comparative psychometric study. | BMC Psychiatry | 13 |  | 222 | 10.1186/1471-244X-13-222 | Wrong comparator |
| Croteau 2019 | Impact of Psychoactive Drug Use on Developing Obesity among Children and Adolescents with Autism Spectrum Diagnosis: A Nested Case-Control Study. | Child Obes | 15 | 2 | 131-141 | 10.1089/chi.2018.0170 | No frequency of weight categories |
| Curtin 2004 | Prevalence of overweight in youth with attention deficit hyperactivity disorder (ADHD) and autism spectrum disorders (ASD): A chart review | Obesity Research | 12 |  | A226-A227 |  | Wrong comparator |
| Curtin 2005 | Prevalence of overweight in children and adolescents with attention deficit hyperactivity disorder and autism spectrum disorders: a chart review. | BMC Pediatr | 5 |  | 48 | 10.1186/1471-2431-5-48 | Wrong comparator |
| Curtin 2014 | Obesity in children with autism spectrum disorder. | Harv Rev Psychiatry | 22 | 2 | 93-103 | 10.1097/HRP.0000000000000031 | Wrong study design |
| Dahlgren 2021 | Physical activity and screen time among youth with autism: A longitudinal analysis from 9 to 18 years | Autism | 25 | 4 | 1090-1099 | 10.1177/1362361320981314 | No frequency of weight categories |
| daSilva 2024 | Eating Behavior and Nutritional Profile of Children with Autism Spectrum Disorder in a Reference Center in the Amazon. | Nutrients | 16 | 3 |  | 10.3390/nu16030452 | Wrong comparator |
| deHoogd 2012 | Differences in body mass index z-scores and weight status in a Dutch pediatric psychiatric population with and without use of second-generation antipsychotics. | J Child Adolesc Psychopharmacol | 22 | 2 | 166-73 | 10.1089/cap.2011.0079 | Wrong comparator |
| Dell'Osso 2018 | Subthreshold autism spectrum disorder in patients with eating disorders. | Compr Psychiatry | 81 |  | 66-72 | 10.1016/j.comppsych.2017.11.007 | Inadequate definition of ASD |
| Demb 2011 | The effect of long-term use of risperidone on body weight of children with an autism spectrum disorder. | J Clin Psychopharmacol | 31 | 5 | 669-70 | 10.1097/JCP.0b013e31822befa9 | Wrong study design |
| DemirAÇ 2022 | The nutritional behavior of children with autism spectrum disorder, parental feeding styles, and anthropometric measurements. | Nord J Psychiatry | 76 | 1 | 64-70 | 10.1080/08039488.2021.1934109 | No frequency of weight categories |
| Demirdögen 2024 | Serum hypoxia-inducible factor-1 alpha (HIF-1α) and apelin levels in children and adolescents diagnosed with autism spectrum disorder | Research in Autism Spectrum Disorders | 112 |  |  | 10.1016/j.rasd.2024.102327 | No frequency of weight categories |
| Dempsey 2019 | Autism Spectrum Disorder Severity, Developmental Delays, and Overweight/Obese Weight Status. | J Pediatr | 205 |  | 9-11 | 10.1016/j.jpeds.2018.10.046 | Wrong study design |
| deVinck-Baroody 2015 | Overweight and Obesity in a Sample of Children With Autism Spectrum Disorder. | Acad Pediatr | 15 | 4 | 396-404 | 10.1016/j.acap.2015.03.008 | No frequency of weight categories |
| Dinkler 2021 | Anorexia nervosa and autism: a prospective twin cohort study. | J Child Psychol Psychiatry | 62 | 3 | 316-326 | 10.1111/jcpp.13265 | Inadequate definition of ASD |
| Dreyer 2008 | Obesity and Overweight among Patients Diagnosed with Autism Spectrum Disorders | Obesity | 16 |  | S284-S284 |  | Wrong study design |
| Dudova 2014 | Comparison of three screening tests for autism in preterm children with birth weights less than 1,500 grams. | Neuropsychiatr Dis Treat | 10 |  | 2201-8 | 10.2147/NDT.S72921 | Perinatal weight |
| Egan 2013 | Obesity in young children with autism spectrum disorders: prevalence and associated factors. | Child Obes | 9 | 2 | 125-31 | 10.1089/chi.2012.0028 | Wrong study design |
| Ekhlaspour 2016 | Bone Density in Adolescents and Young Adults with Autism Spectrum Disorders. | J Autism Dev Disord | 46 | 11 | 3387-3391 | 10.1007/s10803-016-2871-9 | Wrong comparator |
| Emond 2010 | Feeding symptoms, dietary patterns, and growth in young children with autism spectrum disorders. | Pediatrics | 126 | 2 | e337-42 | 10.1542/peds.2009-2391 | No frequency of weight categories |
| Eow 2021 | Body weight status and dietary intake of Malaysian children with Autism Spectrum Disorder | Research in Autism Spectrum Disorders | 84 |  |  | 10.1016/j.rasd.2021.101768 | Wrong comparator |
| Espinoza 2021 | Not just at school: Inclusion of children with autism spectrum disorder in a weight management program in a community pediatric setting. | Autism | 25 | 3 | 642-655 | 10.1177/1362361321993710 | No frequency of weight categories |
| Esteban-Figuerola 2019 | Differences in food consumption and nutritional intake between children with autism spectrum disorders and typically developing children: A meta-analysis | Autism | 23 | 5 | 1079-1095 | 10.1177/1362361318794179 | Other systematic review or meta-analysis |
| Fornaro 2020 | Prominent autistic traits and subthreshold bipolar/mixed features of depression in severe anorexia nervosa. | Braz J Psychiatry | 42 | 2 | 153-161 | 10.1590/1516-4446-2019-0500 | Wrong comparator |
| Fritz 2014 | DIAGNOSTIC SEVERITY AND FUNCTIONAL LIMITATIONS CONTRIBUTE TO INCREASED PREVALENCE OF OBESITY IN AUTISTIC YOUTH | Annals of Behavioral Medicine | 47 |  | S230-S230 |  | Wrong study design |
| Garcia-Argibay 2024 | Trends in Body Mass Index Among Individuals With Neurodevelopmental Disorders. | JAMA Netw Open | 7 | 9 | e2431543 | 10.1001/jamanetworkopen.2024.31543 | Wrong study design |
| Gesi 2017 | Autistic traits in patients with anorexia nervosa, bulimia nervosa or binge eating disorder: A pilot study | European Psychiatry | 41 |  | S100-S100 | 10.1016/j.eurpsy.2017.01.310 | Wrong study design |
| Ghaffari 2009 | Obesity in Children with Autism Spectrum Disorders | Journal of Child and Adolescent Psychopharmacology | 19 | 6 | 788-788 |  | Wrong study design |
| Gholamalizadeh 2022 | The association of body mass index and dietary fat intake with autism in children: a case-control study | Nutrition & Food Science |  |  |  | 10.1108/NFS-12-2021-0366 | No frequency of weight categories |
| Giles 2021 | Do orthorexia and intolerance of uncertainty mediate the relationship between autism spectrum traits and disordered eating symptoms? | Eat Weight Disord | 26 | 7 | 2309-2316 | 10.1007/s40519-020-01094-0 | No frequency of weight categories |
| Gilmore 2022 | The Association Between Obesity and Key Health or Psychosocial Outcomes Among Autistic Adults: A Systematic Review. | J Autism Dev Disord | 52 | 9 | 4035-4043 | 10.1007/s10803-021-05275-3 | Other systematic review or meta-analysis |
| Gonçalves 2023 | Increased monocytes are associated with overweight in children and adolescents with autism spectrum disorder. | Nutr Hosp | 40 | 6 | 1136-1143 | 10.20960/nh.04472 | Wrong comparator |
| Graf-Myles 2013 | Dietary Adequacy of Children with Autism Compared with Controls and the Impact of Restricted Diet | Journal of Developmental & Behavioral Pediatrics | 34 | 7 | 449-459 | 10.1097/DBP.0b013e3182a00d17 | No frequency of weight categories |
| Granich 2016 | Obesity and associated factors in youth with an autism spectrum disorder. | Autism | 20 | 8 | 916-926 | 10.1177/1362361315616345 | Wrong comparator |
| Gray 2024 | Recruitment feasibility and dietary and behavioral patterns in toddlers with ASD: Preliminary results from the Autism Eats program. | Contemp Clin Trials | 146 |  | 107688 | 10.1016/j.cct.2024.107688 | Wrong study design |
| Green 2015 | A review of physical growth in children and adolescents with Autism Spectrum Disorder | Developmental Review | 36 |  | 156-178 | 10.1016/j.dr.2015.02.001 | Wrong study design |
| Guloksuz 2017 | Elevated plasma concentrations of S100 calcium-binding protein B and tumor necrosis factor alpha in children with autism spectrum disorders. | Braz J Psychiatry | 39 | 3 | 195-200 | 10.1590/1516-4446-2015-1843 | No frequency of weight categories |
| Haegele 2021 | Accelerometer measured physical activity among youth with autism and age, sex, and body mass index matched peers: A preliminary study. | Disabil Health J | 14 | 3 | 101102 | 10.1016/j.dhjo.2021.101102 | Wrong comparator |
| Halls 2022 | Examining the relationship between autistic spectrum disorder characteristics and structural brain differences seen in anorexia nervosa. | Eur Eat Disord Rev | 30 | 5 | 459-473 | 10.1002/erv.2910 | Wrong comparator |
| Halls 2023 | Longitudinal exploration of biopsychosocial profiles in individuals with anorexia nervosa. | J Psychiatr Res | 167 |  | 16-22 | 10.1016/j.jpsychires.2023.09.001 | No frequency of weight categories |
| Hambrook 2008 | Empathy, systemizing, and autistic traits in anorexia nervosa: a pilot study. | Br J Clin Psychol | 47 | Pt 3 | 335-9 | 10.1348/014466507X272475 | Inadequate definition of ASD |
| Hammouda 2018 | Assessment of nutritional risk factors predisposing to autism among Saudi children | Nutrafoods | 17 |  | 27-32 |  | Data issues |
| Harris 2022 | Child ADHD and autistic traits, eating behaviours and weight: A population-based study. | Pediatr Obes | 17 | 11 | 12951 | 10.1111/ijpo.12951 | Wrong comparator |
| Hendy 2010 | Parent mealtime actions that mediate associations between children's fussy-eating and their weight and diet. | Appetite | 54 | 1 | 191-5 | 10.1016/j.appet.2009.10.006 | No frequency of weight categories |
| HernándezFernández 2023 | [Nutritional status and food intake frequency in children with autism spectrum disorder]. | Nutr Hosp | 40 | 2 | 347-353 | 10.20960/nh.04258 | Wrong comparator |
| Hsieh 2014 | Obesity and associated factors in adults with intellectual disability | Journal of Intellectual Disability Research | 58 | 9 | 851-863 | 10.1111/jir.12100 | Wrong comparator |
| Huke 2013 | Autism spectrum disorders in eating disorder populations: a systematic review. | Eur Eat Disord Rev | 21 | 5 | 345-51 | 10.1002/erv.2244 | Other systematic review or meta-analysis |
| Huke 2014 | The clinical implications of high levels of autism spectrum disorder features in anorexia nervosa: a pilot study. | Eur Eat Disord Rev | 22 | 2 | 116-21 | 10.1002/erv.2269 | No frequency of weight categories |
| Hwang 2022 | Association between body mass index and subcortical volume in pre-adolescent children with autism spectrum disorder: An exploratory study. | Autism Res | 15 | 12 | 2238-2249 | 10.1002/aur.2834 | No frequency of weight categories |
| Ikejiri 2016 | Reduced growth during early infancy in very low birth weight children with autism spectrum disorder. | Early Hum Dev | 98 |  | 23-7 | 10.1016/j.earlhumdev.2016.05.001 | Perinatal weight |
| Inoue 2016 | Investigating autism spectrum traits in adolescents with anorexia nervosa and avoidant/restrictive food intake disorder. | Journal of the American Academy of Child and Adolescent Psychiatry | 55 | 10 | S151-S151 | 10.1016/j.jaac.2016.09.161 | Wrong study design |
| Inoue 2021 | Prevalence of autism spectrum disorder and autistic traits in children with anorexia nervosa and avoidant/restrictive food intake disorder. | Biopsychosoc Med | 15 | 1 | 9 | 10.1186/s13030-021-00212-3 | Wrong comparator |
| Jacob 2014 | Are there body mass index differences between autistic children and other normally developing children in South India? | Indian Journal of Psychiatry | 56 | 5 | S70-S70 |  | Wrong study design |
| Jeremias 2021 | Diagnostic challenges presented by women with anorexia nervosa and elevated rates of autistic traits | European Psychiatry | 64 |  | S699-S700 | 10.1192/j.eurpsy.2021.1852 | Wrong study design |
| Johnson 2008 | Eating Habits and Dietary Status in Young Children with Autism | Journal of Developmental and Physical Disabilities | 20 | 5 | 437-448 | 10.1007/s10882-008-9111-y | No frequency of weight categories |
| Jones 2016 | A description of medical conditions in adults with autism spectrum disorder: A follow-up of the 1980s Utah/UCLA Autism Epidemiologic Study. | Autism | 20 | 5 | 551-61 | 10.1177/1362361315594798 | Wrong comparator |
| Jones 2017 | Physical activity, sedentary behavior and their correlates in children with Autism Spectrum Disorder: A systematic review. | PLoS One | 12 | 2 | e0172482 | 10.1371/journal.pone.0172482 | Other systematic review or meta-analysis |
| Kahathuduwa 2019 | The risk of overweight and obesity in children with autism spectrum disorders: A systematic review and meta-analysis. | Obes Rev | 20 | 12 | 1667-1679 | 10.1111/obr.12933 | Meta-analysis of interest |
| Kahathuduwa 2022 | Autism spectrum disorder is associated with an increased risk of development of underweight in children and adolescents: A systematic review and meta-analysis | Research in Autism Spectrum Disorders | 94 |  |  | 10.1016/j.rasd.2022.101969 | Meta-analysis of interest |
| Kalayci 2021 | Autistic Traits and Social Responsiveness: The Relationship Between Autistic Traits and Comorbid Psychiatric Symptoms in Adolescents with Anorexia Nervosa. | Noro Psikiyatr Ars | 58 | 4 | 283-288 | 10.29399/npa.27175 | No frequency of weight categories |
| Kalyva 2009 | Comparison of eating attitudes between adolescent girls with and without Asperger syndrome: daughters' and mothers' reports. | J Autism Dev Disord | 39 | 3 | 480-6 | 10.1007/s10803-008-0648-5 | No frequency of weight categories |
| Karjalainen 2016 | Eating disorders and eating pathology in young adult and adult patients with ESSENCE. | Compr Psychiatry | 66 |  | 79-86 | 10.1016/j.comppsych.2015.12.009 | Wrong comparator |
| Karjalainen 2019 | Do autism spectrum disorder and anorexia nervosa have some eating disturbances in common? | Eur Child Adolesc Psychiatry | 28 | 1 | 69-78 | 10.1007/s00787-018-1188-y | No frequency of weight categories |
| Katoda 1991 | Height and weight of Tokyo schoolchildren with and without intellectual handicaps. | Ann Hum Biol | 18 | 4 | 327-39 | 10.1080/03014469100001642 | No frequency of weight categories |
| Kawicka 2013 | How nutritional status, diet and dietary supplements can affect autism. A review. | Rocz Panstw Zakl Hig | 64 | 1 | 1-12 |  | Wrong study design |
| Kerr-Gaffney 2020 | The social responsiveness scale is an efficient screening tool for autism spectrum disorder traits in adults with anorexia nervosa. | Eur Eat Disord Rev | 28 | 4 | 433-444 | 10.1002/erv.2736 | No frequency of weight categories |
| Kerr-Gaffney 2020 | Autism spectrum disorder traits are associated with empathic abilities in adults with anorexia nervosa. | J Affect Disord | 266 |  | 273-281 | 10.1016/j.jad.2020.01.169 | No frequency of weight categories |
| Kerr-Gaffney 2021 | Autism symptoms in anorexia nervosa: a comparative study with females with autism spectrum disorder. | Mol Autism | 12 | 1 | 47 | 10.1186/s13229-021-00455-5 | No frequency of weight categories |
| Kim 2022 | Investigating socioecological obesogenic factors in children with Autism Spectrum Disorder. | Front Public Health | 10 |  | 867456 | 10.3389/fpubh.2022.867456 | No frequency of weight categories |
| Kinnaird 2019 | Exploring Self-Reported Eating Disorder Symptoms in Autistic Men. | Autism Adulthood | 1 | 4 | 306-310 | 10.1089/aut.2019.0017 | No frequency of weight categories |
| Kinnaird 2020 | Pragmatic Sensory Screening in Anorexia Nervosa and Associations with Autistic Traits. | J Clin Med | 9 | 4 |  | 10.3390/jcm9041182 | Wrong comparator |
| Kinnaird 2020 | Interoception in Anorexia Nervosa: Exploring Associations With Alexithymia and Autistic Traits. | Front Psychiatry | 11 |  | 64 | 10.3389/fpsyt.2020.00064 | Wrong comparator |
| Kinter 2023 | The Sensory Profiles, Eating Behaviors, and Quality of Life of Children with Autism Spectrum Disorder and Avoidant/Restrictive Food Intake Disorder | Psychiatric Quarterly |  |  |  | 10.1007/s11126-023-10063-6 | No frequency of weight categories |
| Kirkovski 2025 | Autism spectrum disorder and anorexia nervosa: Investigating the behavioural and neurocognitive overlap. | Psychiatry Res | 344 |  | 116285 | 10.1016/j.psychres.2024.116285 | No frequency of weight categories |
| Kitajima 2022 | Sensory processing in children and adolescents shortly after the onset of anorexia nervosa: a pilot study. | Biopsychosoc Med | 16 | 1 | 27 | 10.1186/s13030-022-00256-z | Inadequate definition of ASD |
| Kittana 2023 | Nutritional Status and Feeding Behavior of Children with Autism Spectrum Disorder in the Middle East and North Africa Region: A Systematic Review. | Nutrients | 15 | 3 |  | 10.3390/nu15030711 | Other systematic review or meta-analysis |
| Koch 2015 | Autism spectrum disorder in individuals with anorexia nervosa and in their first- and second-degree relatives: Danish nationwide register-based cohort-study. | Br J Psychiatry | 206 | 5 | 401-7 | 10.1192/bjp.bp.114.153221 | No frequency of weight categories |
| Kupis 2021 | Altered patterns of brain dynamics linked with body mass index in youth with autism. | Autism Res | 14 | 5 | 873-886 | 10.1002/aur.2488 | No frequency of weight categories |
| Kwon 2022 | Association of 3-hydroxy-3-methylglutaryl-coenzyme A reductase gene polymorphism with obesity and lipid metabolism in children and adolescents with autism spectrum disorder. | Metab Brain Dis | 37 | 2 | 319-328 | 10.1007/s11011-021-00877-3 | No frequency of weight categories |
| Kyler 2022 | Prevalence and Severity of Chronic Conditions among Adolescents with Obesity | Childhood Obesity |  |  |  | 10.1089/chi.2022.0217 | Wrong comparator |
| Li 2018 | Weight status, diet quality, perceived stress, and functional health of caregivers of children with autism spectrum disorder. | J Spec Pediatr Nurs | 23 | 1 |  | 10.1111/jspn.12205 | Wrong comparator |
| Li 2020 | Global prevalence of obesity, overweight and underweight in children, adolescents and adults with autism spectrum disorder, attention-deficit hyperactivity disorder: A systematic review and meta-analysis. | Obes Rev | 21 | 12 | e13123 | 10.1111/obr.13123 | Other systematic review or meta-analysis |
| Li 2021 | A Systematic Review and Meta-Analysis of Moderate-to-Vigorous Physical Activity Levels in Children and Adolescents With and Without ASD in Inclusive Schools | Frontiers in Pediatrics | 9 |  |  | 10.3389/fped.2021.726942 | Other systematic review or meta-analysis |
| Lin 2024 | The relationship between hair cortisol concentration and autism diagnosis. | J Psychiatr Res | 176 |  | 68-76 | 10.1016/j.jpsychires.2024.05.052 | Wrong study design |
| Liu 2017 | The Relationship of Motor Proficiency, Nutrition Knowledge and BMI in Children with Autism Spectrum Disorder | Journal of Sport & Exercise Psychology | 39 |  | S78-S78 |  | Wrong comparator |
| Liu 2018 | Parent-reported prevalence and persistence of 19 common child health conditions. | Arch Dis Child | 103 | 6 | 548-556 | 10.1136/archdischild-2017-313191 | No frequency of weight categories |
| Liu 2019 | Motor Proficiency, Sensory Processing, and BMI in Children with Autism Spectrum Disorder | Journal of Sport & Exercise Psychology | 41 |  | S15-S16 |  | Wrong study design |
| Lourenço 2014 | The Influence Of 20 Week Trampoline Training On Body Mass Index And Motor Proficiency In Children With Autism Spectrum Disorders | Medicine and Science in Sports and Exercise | 46 | 5 | 234-234 | 10.1249/01.mss.0000493890.80494.52 | Wrong study design |
| Ludyga 2021 | Muscle strength and executive function in children and adolescents with autism spectrum disorder. | Autism Res | 14 | 12 | 2555-2563 | 10.1002/aur.2587 | No frequency of weight categories |
| Magaña 2023 | Associations between parenting strategies and BMI percentile among Latino children and youth with intellectual and developmental disabilities. | Front Pediatr | 11 |  | 1189686 | 10.3389/fped.2023.1189686 | Wrong comparator |
| Malhi 2017 | Feeding Problems and Nutrient Intake in Children with and without Autism: A Comparative Study. | Indian J Pediatr | 84 | 4 | 283-288 | 10.1007/s12098-016-2285-x | No frequency of weight categories |
| Mansour 2016 | Emotions mediate the relationship between autistic traits and disordered eating: A new autistic-emotional model for eating pathology | Psychiatry Research | 245 |  | 119-126 | 10.1016/j.psychres.2016.08.021 | No frequency of weight categories |
| Mansour 2017 | ADHD severity as it relates to comorbid psychiatric symptomatology in children with Autism Spectrum Disorders (ASD) | Research in Developmental Disabilities | 60 |  | 52-64 | 10.1016/j.ridd.2016.11.009 | No frequency of weight categories |
| Marí-Bauset 2015 | Anthropometric measurements and nutritional assessment in autism spectrum disorders: A systematic review | Research in Autism Spectrum Disorders | 9 |  | 130-143 | 10.1016/j.rasd.2014.08.012 | Other systematic review or meta-analysis |
| Marí-Bauset 2016 | Fat intake in children with autism spectrum disorder in the Mediterranean region (Valencia, Spain) | Nutritional Neuroscience | 19 | 9 | 377-386 | 10.1179/1476830515Y.0000000029 | No frequency of weight categories |
| Martinez-Cayuelas 2024 | Sleep Problems, Circadian Rhythms, and Their Relation to Behavioral Difficulties in Children and Adolescents with Autism Spectrum Disorder. | J Autism Dev Disord | 54 | 5 | 1712-1726 | 10.1007/s10803-023-05934-7 | Wrong comparator |
| McCarron 2019 | Weight Status in Children with Autism Spectrum Disorder. | Ir Med J | 112 | 3 | 904 |  | Wrong study design |
| McCoy 2015 | Obesity, Physical Activity And Sedentary Behavior In Youth With And Without Autism Spectrum Disorder | Medicine and Science in Sports and Exercise | 47 | 5 | 922-922 | 10.1249/01.mss.0000479232.74031.c4 | Wrong study design |
| Meguid 2017 | Dietary adequacy of Egyptian children with autism spectrum disorder compared to healthy developing children. | Metab Brain Dis | 32 | 2 | 607-615 | 10.1007/s11011-016-9948-1 | No frequency of weight categories |
| Mellström 2020 | Methylphenidate and Reduced Overweight in Children With ADHD | Journal of Attention Disorders | 24 | 2 | 246-254 | 10.1177/1087054718808045 | Wrong study design |
| Micai 2023 | Prevalence of co-occurring conditions in children and adults with autism spectrum disorder: A systematic review and meta-analysis. | Neurosci Biobehav Rev | 155 |  | 105436 | 10.1016/j.neubiorev.2023.105436 | Other systematic review or meta-analysis |
| Moludi 2019 | Comparison of dietary macro and micronutrient intake with physical activity levels among children with and without autism: a case-control study | Progress in Nutrition | 21 |  | 49-55 | 10.23751/pn.v21i2-S.6578 | No frequency of weight categories |
| Moses 2014 | Metabolic profiles in adults with autism spectrum disorder and intellectual disabilities. | Eur Psychiatry | 29 | 7 | 397-401 | 10.1016/j.eurpsy.2013.05.005 | No frequency of weight categories |
| Mouridsen 2002 | Body mass index in male and female children with infantile autism. | Autism | 6 | 2 | 197-205 | 10.1177/1362361302006002006 | Inadequate definition of ASD |
| Muscatello 2022 | Diagnostic and Physical Effects in Parasympathetic Response to Social Evaluation in Youth With and Without Autism Spectrum Disorder. | J Autism Dev Disord | 52 | 8 | 3427-3442 | 10.1007/s10803-021-05224-0 | No frequency of weight categories |
| Myers 2017 | Minor physical anomalies in neurodevelopmental disorders: a twin study. | Child Adolesc Psychiatry Ment Health | 11 |  | 57 | 10.1186/s13034-017-0195-y | No frequency of weight categories |
| Nagai 2020 | Primitive Reflexes in Very Low Birth Weight Infants Later Diagnosed with Autism Spectrum Disorder | Minerva Pediatrics |  |  |  | 10.23736/S0026-4946.20.05784-9 | Perinatal weight |
| Nasef 2017 | Prevalence of obesity in children with autism- A West Wales Study | European Journal of Pediatrics | 176 | 11 | 1542-1542 |  | Wrong study design |
| Nazar 2018 | An examination of the clinical outcomes of adolescents and young adults with broad autism spectrum traits and autism spectrum disorder and anorexia nervosa: A multi centre study. | Int J Eat Disord | 51 | 2 | 174-179 | 10.1002/eat.22823 | Wrong study design |
| Neumeyer 2013 | Bone density in peripubertal boys with autism spectrum disorders. | J Autism Dev Disord | 43 | 7 | 1623-9 | 10.1007/s10803-012-1709-3 | No frequency of weight categories |
| Neumeyer 2017 | Bone microarchitecture in adolescent boys with autism spectrum disorder. | Bone | 97 |  | 139-146 | 10.1016/j.bone.2017.01.009 | No frequency of weight categories |
| Neumeyer 2018 | Nutrition and Bone Density in Boys with Autism Spectrum Disorder. | J Acad Nutr Diet | 118 | 5 | 865-877 | 10.1016/j.jand.2017.11.006 | No frequency of weight categories |
| Nickel 2019 | Systematic Review: Overlap Between Eating, Autism Spectrum, and Attention-Deficit/Hyperactivity Disorder. | Front Psychiatry | 10 |  | 708 | 10.3389/fpsyt.2019.00708 | Other systematic review or meta-analysis |
| Nielsen 2015 | Effects of autism spectrum disorders on outcome in teenage-onset anorexia nervosa evaluated by the Morgan-Russell outcome assessment schedule: a controlled community-based study. | Mol Autism | 6 |  | 14 | 10.1186/s13229-015-0013-4 | No frequency of weight categories |
| Nielsen 2022 | Effects of autism on 30-year outcome of anorexia nervosa. | J Eat Disord | 10 | 1 | 4 | 10.1186/s40337-021-00518-1 | No frequency of weight categories |
| Nuyttens 2024 | A longitudinal study of autism spectrum disorder characteristics in adolescents with restrictive type anorexia nervosa during and after underweight. | Eur Eat Disord Rev | 32 | 2 | 310-321 | 10.1002/erv.3042 | Wrong study design |
| OjedaBenítez 2013 | Perfil nutricional de niños y adolescentes con trastornos del espectro autista del área metropolitana de Asunción | Pediatría (Asunción) | 40 | 2 | 133-143 |  | Wrong comparator |
| Padmanabhan 2020 | The relationship between sensory integration challenges and the dietary intake and nutritional status of children with Autism Spectrum Disorders in Mumbai, India | International Journal of Developmental Disabilities | 66 | 2 | 142-152 | 10.1080/20473869.2018.1522816 | Wrong comparator |
| Page 2022 | Correlates of Feeding Difficulties Among Children with Autism Spectrum Disorder: A Systematic Review. | J Autism Dev Disord | 52 | 1 | 255-274 | 10.1007/s10803-021-04947-4 | Other systematic review or meta-analysis |
| Pan 2016 | Objectively Measured Physical Activity and Health-Related Physical Fitness in Secondary School-Aged Male Students With Autism Spectrum Disorders. | Phys Ther | 96 | 4 | 511-20 | 10.2522/ptj.20140353 | No frequency of weight categories |
| Pan 2023 | Genetic and environmental contributions to co-occurring physical health conditions in autism spectrum condition and attention-deficit/hyperactivity disorder | Molecular Autism | 14 | 1 |  | 10.1186/s13229-023-00548-3 | Wrong study design |
| Peng 2022 | Meta-analysis and systematic review of physical activity on neurodevelopment disorders, depression, and obesity among children and adolescents. | Front Psychol | 13 |  | 940977 | 10.3389/fpsyg.2022.940977 | Other systematic review or meta-analysis |
| Pham 2020 | Obesity and Severe Obesity in Children with Autism Spectrum Disorder: Prevalence and Risk Factors. | South Med J | 113 | 4 | 168-175 | 10.14423/SMJ.0000000000001068 | Wrong comparator |
| Pitchford 2021 | Differences in Motor Incoordination Among Youth With Autism Spectrum Disorder by Sex and Clinical Obesity | Journal of Sport & Exercise Psychology | 43 |  | S17-S17 |  | Wrong study design |
| Poletti 2019 | Autism spectrum disorder presenting with acute anorectic symptomatology: a diagnostic challenge | Journal of Psychopathology | 25 | 1 | 45-47 |  | Wrong study design |
| Postorino 2017 | Investigation of Autism Spectrum Disorder and Autistic Traits in an Adolescent Sample with Anorexia Nervosa. | J Autism Dev Disord | 47 | 4 | 1051-1061 | 10.1007/s10803-016-3023-y | Wrong study design |
| Ptomey 2020 | Weight status and associated comorbidities in children and adults with Down syndrome, autism spectrum disorder and intellectual and developmental disabilities. | J Intellect Disabil Res | 64 | 9 | 725-737 | 10.1111/jir.12767 | Wrong comparator |
| Ranjan 2015 | Nutritional status of individuals with autism spectrum disorders: do we know enough? | Adv Nutr | 6 | 4 | 397-407 | 10.3945/an.114.007914 | Wrong study design |
| Råstam 2003 | Outcome of teenage-onset anorexia nervosa in a Swedish community-based sample. | Eur Child Adolesc Psychiatry | 12 Suppl 1 |  | I78-90 | 10.1007/s00787-003-1111-y | No frequency of weight categories |
| Remeis 2023 | Neighborhood Factors Influencing Body Mass Index in Children With Autism Spectrum Disorder | American Journal of Occupational Therapy | 77 |  |  | 10.5014/ajot.2023.77S2-PO31 | Wrong study design |
| Rosa 2016 | Socioeconomic status and intelligence quotient as predictors of psychiatric disorders in children and adolescents with high-functioning autism spectrum disorder and in their siblings | Autism | 20 | 8 | 963-972 | 10.1177/1362361315617881 | No frequency of weight categories |
| Rothery 1988 | Anorexia nervosa and infantile autism. | Br J Psychiatry | 153 |  | 714 | 10.1192/bjp.153.5.714 | Wrong study design |
| Ruggieri 2017 | Prevalence of obesity in autistic youth from 2003 to 2011: trends in environmental and behavioral risk factors | Annals of Behavioral Medicine | 51 |  | S2687-S2688 |  | Wrong study design |
| Rutter 1995 | Relationships between mental disorders in childhood and adulthood. | Acta Psychiatr Scand | 91 | 2 | 73-85 | 10.1111/j.1600-0447.1995.tb09745.x | Wrong study design |
| Rydzewska 2021 | Umbrella systematic review of systematic reviews and meta-analyses on comorbid physical conditions in people with autism spectrum disorder. | Br J Psychiatry | 218 | 1 | 10-19 | 10.1192/bjp.2020.167 | Other systematic review or meta-analysis |
| Şahin 2023 | Evaluation of carotid intima media thickness as an early marker of atherosclerosis in children and adolescents with autism spectrum disorder: a case-control study. | Int J Dev Disabil | 69 | 6 | 825-834 | 10.1080/20473869.2021.2024954 | No frequency of weight categories |
| Sammels 2022 | Autism Spectrum Disorder and Obesity in Children: A Systematic Review and Meta-Analysis. | Obes Facts | 15 | 3 | 305-320 | 10.1159/000523943 | Meta-analysis of interest |
| Santos 2023 | Anorexia Nervosa and Autism Spectrum Disorder: what links them? | European Psychiatry | 66 |  | S523-S523 | 10.1192/j.eurpsy.2023.1110 | Wrong study design |
| Sapkota 2023 | Accommodation and disability-specific differences in nutritional status of children with disabilities in Kathmandu, Nepal: A cross-sectional study. | BMC Public Health | 23 | 1 | 315 | 10.1186/s12889-023-14999-z | Wrong comparator |
| Saure 2021 | Anorexia nervosa and comorbid autism spectrum disorders. | Curr Opin Psychiatry | 34 | 6 | 569-575 | 10.1097/YCO.0000000000000742 | Wrong study design |
| Saure 2022 | Atypical Sensory Processing Is Associated With Lower Body Mass Index and Increased Eating Disturbance in Individuals With Anorexia Nervosa. | Front Psychiatry | 13 |  | 850594 | 10.3389/fpsyt.2022.850594 | Wrong comparator |
| Schaumberg 2021 | Prospective associations between childhood social communication processes and adolescent eating disorder symptoms in an epidemiological sample. | Eur Child Adolesc Psychiatry | 30 | 12 | 1929-1938 | 10.1007/s00787-020-01655-9 | No frequency of weight categories |
| Schmitt 2008 | A Comparison of Nutrient Intake and Eating Behaviors of Boys With and Without Autism | Topics in Clinical Nutrition | 23 | 1 | 23-31 | 10.1097/01.TIN.0000312077.45953.6c | No frequency of weight categories |
| Schreck 2004 | A Comparison of Eating Behaviors Between Children with and Without Autism | Journal of Autism and Developmental Disorders | 34 | 4 | 433-438 | 10.1023/B:JADD.0000037419.78531.86 | No frequency of weight categories |
| Schwarz 2024 | Body mass index is an overlooked confounding factor in existing clustering studies of 3D facial scans of children with autism spectrum disorder. | Sci Rep | 14 | 1 | 9873 | 10.1038/s41598-024-60376-0 | No frequency of weight categories |
| Seiverling 2011 | The Screening Tool of Feeding Problems applied to children (STEP-CHILD): psychometric characteristics and associations with child and parent variables. | Res Dev Disabil | 32 | 3 | 1122-9 | 10.1016/j.ridd.2011.01.012 | No frequency of weight categories |
| Sharp 2014 | Weight Status and Growth Patterns Among Children With Autism Living in North Carolina | Topics in Clinical Nutrition | 29 | 1 | 10-14 | 10.1097/01.TIN.0000443022.75534.3b | Wrong comparator |
| Shinawi 2011 | 11p14.1 microdeletions associated with ADHD, autism, developmental delay, and obesity. | Am J Med Genet A | 155A | 6 | 1272-80 | 10.1002/ajmg.a.33878 | Wrong study design |
| Shmaya 2015 | Nutritional deficiencies and overweight prevalence among children with autism spectrum disorder. | Res Dev Disabil | 38 |  | 1-6 | 10.1016/j.ridd.2014.11.020 | No frequency of weight categories |
| Shuster 2023 | Neurobehavioral and Medical Correlates of Autism Screening: 2-Year Outcomes for Infants Born Very Preterm | Journal of Pediatrics | 260 |  |  | 10.1016/j.jpeds.2023.113536 | Wrong comparator |
| SilvaDVD 2020 | EXCESS WEIGHT AND GASTROINTESTINAL SYMPTOMS IN A GROUP OF AUTISTIC CHILDREN. | Rev Paul Pediatr | 38 |  | e2019080 | 10.1590/1984-0462/2020/38/2019080 | Wrong comparator |
| Simpson 2024 | Safety and Efficacy of Paliperidone Palmitate in Pediatric Patients with Autism and Intellectual Disability. | J Autism Dev Disord |  |  |  | 10.1007/s10803-024-06372-9 | Wrong comparator |
| Sobanski 1999 | Further evidence for a low body weight in male children and adolescents with Asperger's disorder. | Eur Child Adolesc Psychiatry | 8 | 4 | 312-4 | 10.1007/s007870050106 | Wrong comparator |
| Soncini 2020 | Association Between Prematurity and Diagnosis of Neurodevelopment Disorder: A Case-Control Study | Journal of Autism and Developmental Disorders | 50 | 1 | 145-152 | 10.1007/s10803-019-04235-2 | Perinatal weight |
| Souza 2012 | Intestinal permeability and nutritional status in developmental disorders. | Altern Ther Health Med | 18 | 2 | 19-24 |  | Wrong comparator |
| SPadmanabhan 2018 | The relationship between sensory integration challenges and the dietary intake and nutritional status of children with Autism Spectrum Disorders in Mumbai, India. | Int J Dev Disabil | 66 | 2 | 142-152 | 10.1080/20473869.2018.1522816 | Wrong comparator |
| Steinhausen 2021 | Comorbid mental disorders during long-term course in a nationwide cohort of patients with anorexia nervosa. | Int J Eat Disord | 54 | 9 | 1608-1618 | 10.1002/eat.23570 | No frequency of weight categories |
| Strang-Karlsson 2011 | Autism- and Asperger-Related Traits in Young Adults Born Preterm: The Helsinki Study of Very Low Birth Weight Adults | Journal of Developmental Origins of Health and Disease | 2 |  | S146-S146 |  | Wrong study design |
| Sun 2013 | Nutritional status survey of children with autism and typically developing children aged 4-6 years in Heilongjiang Province, China. | J Nutr Sci | 2 |  | e16 | 10.1017/jns.2013.9 | No frequency of weight categories |
| Sung 2020 | Body composition of school-aged children with disabilities. | Pediatr Int | 62 | 8 | 962-969 | 10.1111/ped.14248 | Inadequate definition of ASD |
| Tahech 2023 | Differences in Mealtime Behaviors, Food Security, and Weight Concerns Between Neurotypical Youth and Youth with Autism Spectrum Disorder | Advances in Neurodevelopmental Disorders |  |  |  | 10.1007/s41252-023-00329-2 | No frequency of weight categories |
| Tahech 2024 | Differences in Mealtime Behaviors, Food Security, and Weight Concerns Between Neurotypical Youth and Youth with Autism Spectrum Disorder | Advances in Neurodevelopmental Disorders | 8 | 2 | 262-270 | 10.1007/s41252-023-00329-2 | No frequency of weight categories |
| Tchanturia 2013 | Exploring autistic traits in anorexia: a clinical study. | Mol Autism | 4 | 1 | 44 | 10.1186/2040-2392-4-44 | No frequency of weight categories |
| Thorsteinsdottir 2022 | Odds of fussy eating are greater among children with obesity and anxiety. | Obes Sci Pract | 8 | 1 | 91-100 | 10.1002/osp4.548 | Wrong comparator |
| Thorsteinsdottir 2023 | Body Composition in Fussy-Eating Children, with and without Neurodevelopmental Disorders, and Their Parents, Following a Taste Education Intervention. | Nutrients | 15 | 12 |  | 10.3390/nu15122788 | Wrong comparator |
| Tonacci 2019 | Autistic traits impact on olfactory processing in adolescent girls with Anorexia Nervosa restricting type. | Psychiatry Res | 274 |  | 20-26 | 10.1016/j.psychres.2019.02.026 | No frequency of weight categories |
| Toy 2016 | Autistic traits in women with primary dysmenorrhea: a case-control study. | Neuropsychiatr Dis Treat | 12 |  | 2319-25 | 10.2147/NDT.S114439 | Wrong comparator |
| Tyler 2014 | Physical activity and physical fitness of school-aged children and youth with autism spectrum disorders. | Autism Res Treat | 2014 |  | 312163 | 10.1155/2014/312163 | No frequency of weight categories |
| Ugur 2018 | Investigation of autism spectrum disorder and autistic symptoms in obese children and adolescents | Anatolian Journal of Psychiatry | 19 | 5 | 518-525 | 10.5455/apd.289937 | Wrong comparator |
| Usui 2020 | VLDL-specific increases of fatty acids in autism spectrum disorder correlate with social interaction. | EBioMedicine | 58 |  | 102917 | 10.1016/j.ebiom.2020.102917 | No frequency of weight categories |
| vanderLubbe 2024 | Novel Insights into Obesity in Preschool Children with Autism Spectrum Disorder. | Child Psychiatry Hum Dev |  |  |  | 10.1007/s10578-024-01679-1 | No frequency of weight categories |
| vanderLubbe 2025 | Hair Cortisol in Young Children with Autism and Their Parents: Associations with Child Mental Health, Eating Behavior and Weight Status. | J Autism Dev Disord |  |  |  | 10.1007/s10803-024-06672-0 | Wrong comparator |
| Vanwong 2020 | Risperidone-Induced Obesity in Children and Adolescents With Autism Spectrum Disorder: Genetic and Clinical Risk Factors. | Front Pharmacol | 11 |  | 565074 | 10.3389/fphar.2020.565074 | Wrong study design |
| Wallace 2018 | Autism spectrum disorder and food neophobia: clinical and subclinical links. | Am J Clin Nutr | 108 | 4 | 701-707 | 10.1093/ajcn/nqy163 | Wrong comparator |
| Ward 2023 | Increased rates of chronic physical health conditions across all organ systems in autistic adolescents and adults. | Mol Autism | 14 | 1 | 35 | 10.1186/s13229-023-00565-2 | Wrong comparator |
| Weir 2021 | Increased prevalence of non-communicable physical health conditions among autistic adults. | Autism | 25 | 3 | 681-694 | 10.1177/1362361320953652 | No frequency of weight categories |
| Westwood 2016 | Using the Autism-Spectrum Quotient to Measure Autistic Traits in Anorexia Nervosa: A Systematic Review and Meta-Analysis. | J Autism Dev Disord | 46 | 3 | 964-77 | 10.1007/s10803-015-2641-0 | Other systematic review or meta-analysis |
| Whiteley 2004 | Body mass index of children from the United Kingdom diagnosed with pervasive developmental disorders. | Pediatr Int | 46 | 5 | 531-3 | 10.1111/j.1442-200x.2004.01946.x | Wrong comparator |
| Xia 2010 | A preliminary study on nutritional status and intake in Chinese children with autism. | Eur J Pediatr | 169 | 10 | 1201-6 | 10.1007/s00431-010-1203-x | Wrong comparator |
| Xiong 2009 | The physical status of children with autism in China. | Res Dev Disabil | 30 | 1 | 70-6 | 10.1016/j.ridd.2007.11.001 | Wrong comparator |
| Yılmaz 2024 | A comparison of the physical fitness of individuals with intellectually disabilities autism spectrum disorders and Down syndrome diagnosis. | Int J Dev Disabil | 70 | 3 | 397-405 | 10.1080/20473869.2022.2102882 | Wrong comparator |
| Young 2022 | The Relationship Between Autism Diagnostic Criteria and Problematic Eating Behaviors | Advances in Neurodevelopmental Disorders | 6 | 1 | 53-64 | 10.1007/s41252-021-00231-9 | No frequency of weight categories |
| Zhao 2024 | Underweight, overweight, obesity and associated factors in children and adolescents with autism spectrum disorder in China | Research in Autism Spectrum Disorders | 115 |  |  | 10.1016/j.rasd.2024.102414 | Wrong comparator |
| Zheng 2017 | Association among obesity, overweight and autism spectrum disorder: a systematic review and meta-analysis. | Sci Rep | 7 | 1 | 11697 | 10.1038/s41598-017-12003-4 | Meta-analysis of interest |
| Zhong 2022 | Correlates of Physical Activity of Children and Adolescents with Autism Spectrum Disorder in Low- and Middle-Income Countries: A Systematic Review of Cross-Sectional Studies. | Int J Environ Res Public Health | 19 | 23 |  | 10.3390/ijerph192316301 | Other systematic review or meta-analysis |
| Zou 2024 | Correlation of Co-Morbidities with Symptom Severity of Children with Autism Spectrum Disorder: A Cross-Sectional Survey. | Nutrients | 16 | 17 |  | 10.3390/nu16172960 | No frequency of weight categories |
| ZuritaOrtega 2010 | Aportaciones a la determinación de la prevalencia de la obesidad entre el alumnado de educación especial | Pediatría Atención Primaria | 12 | 45 | 15-31 | 10.4321/s1139-76322010000100003 | Wrong comparator |

Table S5 Included studies with multiple reports

Studies are named following the first author and year of the key report for that study. All reports identified as pertaining to the same study are presented under the header of its study in alphabetical order. Bolded references indicate the key report for a given study.

| **Al-Kindi 2016** | |
| --- | --- |
|  | Al-Kindi, N. M., Al-Farsi, Y. M., Al-Bulushi, B., Ali, A., Rizvi, S. G. A., & Essa, M. M. (2020). Food Selection and Preferences of Omani Autistic Children (pp. 505–523). https://doi.org/10.1007/978-3-030-30402-7_16 |
|  | Al-Kindi, N. M., Al-Farsi, Y. M., Waly, M. I., A-Shafaee, M. S., & Bakheit, C. S. (2016). Dietary Intake and Food Preferences of Autistic Children Versus Children with Typical Development: A Comparative Cross-Sectional Study. EC Nutrition, 6, 72–85. |
| **Ames 2022** | |
|  | Ames, J. L., Morgan, E. H., Giwa Onaiwu, M., Qian, Y., Massolo, M. L., & Croen, L. A. (2022). Racial/Ethnic Differences in Psychiatric and Medical Diagnoses Among Autistic Adults. Autism in Adulthood, 4(4), 290‑305. https://doi.org/10.1089/aut.2021.0083 |
|  | Croen, L. A., Zerbo, O., Qian, Y., Massolo, M. L., Rich, S., Sidney, S., & Kripke, C. (2015). The health status of adults on the autism spectrum. Autism, 19(7), 814‑823. https://doi.org/10.1177/1362361315577517 |
| **Bandini 2013** | |
|  | Bandini, L. G., Anderson, S. E., Curtin, C., Cermak, S., Evans, E. W., Scampini, R., Maslin, M., & Must, A. (2010). Food Selectivity in Children with Autism Spectrum Disorders and Typically Developing Children. The Journal of Pediatrics, 157(2), 259–264. https://doi.org/10.1016/j.jpeds.2010.02.013 |
|  | Bandini, L. G., Gleason, J., Curtin, C., Lividini, K., Anderson, S. E., Cermak, S. A., Maslin, M., & Must, A. (2013). Comparison of physical activity between children with autism spectrum disorders and typically developing children. Autism, 17(1), 44–54. https://doi.org/10.1177/1362361312437416 |
|  | Evans, E. W., Must, A., Anderson, S. E., Curtin, C., Scampini, R., Maslin, M., & Bandini, L. (2012). Dietary patterns and body mass index in children with autism and typically developing children. Research in Autism Spectrum Disorders, 6(1), 399–405. https://doi.org/10.1016/j.rasd.2011.06.014 |
|  | Must, A., Phillips, S. M., Curtin, C., Anderson, S. E., Maslin, M., Lividini, K., & Bandini, L. G. (2014). Comparison of sedentary behaviors between children with autism spectrum disorders and typically developing children. Autism, 18(4), 376–384. https://doi.org/10.1177/1362361313479039 |
| **Błażewicz 2020** | |
|  | Błażewicz, A., Szymańska, I., Dolliver, W., Suchocki, P., Turło, J., Makarewicz, A., & Skórzyńska-Dziduszko, K. (2020). Are Obese Patients with Autism Spectrum Disorder More Likely to Be Selenium Deficient? Research Findings on Pre- and Post-Pubertal Children. Nutrients, 12(11), 3581. https://doi.org/10.3390/nu12113581 |
|  | Skórzyńska-Dziduszko, K. E., Makarewicz, A., & Błażewicz, A. (2023). Peripubertal Alterations of Leptin Levels in Patients with Autism Spectrum Disorder and Elevated or Normal Body Weight. International Journal of Molecular Sciences, 24(5), 4878. https://doi.org/10.3390/ijms24054878 |
| **Buro 2022** | |
|  | Buro, A. W., Salinas-Miranda, A., Marshall, J., Gray, H. L., & Kirby, R. S. (2022). Correlates of obesity in adolescents with and without autism spectrum disorder : The 2017–2018 National Survey of Children’s Health. Disability and Health Journal, 15(2), 101221. https://doi.org/10.1016/j.dhjo.2021.101221 |
|  | Buro, A. W., Salinas‐Miranda, A., Marshall, J., Gray, H. L., & Kirby, R. S. (2022). Obesity and neurodevelopmental and mental health conditions among adolescents aged 10–17 years : The National Survey of Children’s Health 2017–2018. Journal of Paediatrics and Child Health, 58(10), 1753‑1759. https://doi.org/10.1111/jpc.16081 |
|  | Buro, A. W., Salinas-Miranda, A., Marshall, J., Gray, H. L., & Kirby, R. S. (2023). Autism Spectrum Disorder Diagnosis and Other Child, Family, and Community Risk Factors for Obesity among Children and Adolescents Aged 10–17 Years in the United States : A Mediation Analysis. Childhood Obesity, 19(1), 57‑67. https://doi.org/10.1089/chi.2021.0260 |
|  | Must, A., Eliasziw, M., Bandini, L. G., Curtin, C., Magaña, S., & Rancaño, K. M. (2025). The Full Range of Weight Status by Race and Ethnicity in Children with and without Autism in the United States : A Cross-Sectional Study. The Journal of Pediatrics, 280, 114482. https://doi.org/10.1016/j.jpeds.2025.114482 |
| **Curtin 2010** | |
|  | Chen, A. Y., Kim, S. E., Houtrow, A. J., & Newacheck, P. W. (2010). Prevalence of Obesity Among Children With Chronic Conditions. Obesity, 18(1), 210‑213. https://doi.org/10.1038/oby.2009.185 |
|  | Curtin, C., Anderson, S. E., Must, A., & Bandini, L. (2010). The prevalence of obesity in children with autism : A secondary data analysis using nationally representative data from the National Survey of Children’s Health. BMC Pediatrics, 10(1), 11. https://doi.org/10.1186/1471-2431-10-11 |
| **Davignon 2018** | |
|  | Davignon, M. N., Qian, Y., Massolo, M., & Croen, L. A. (2018). Psychiatric and Medical Conditions in Transition-Aged Individuals With ASD. Pediatrics, 141(Supplement_4), S335‑S345. https://doi.org/10.1542/peds.2016-4300K |
|  | Malow, B. A., Qian, Y., Ames, J. L., Alexeeff, S., & Croen, L. A. (2023). Health conditions in autism : Defining the trajectory from adolescence to early adulthood. Autism Research, 16(7), 1437‑1449. https://doi.org/10.1002/aur.2960 |
| **Garcia 2023** | |
|  | Garcia, J. M., & Odahowski, C. L. (2023). An urban versus rural comparison of obesity between youth with and without autism spectrum disorder. Autism Research, 16(1), 200‑207. https://doi.org/10.1002/aur.2856 |
|  | Must, A., Eliasziw, M., Bandini, L. G., Curtin, C., Magaña, S., & Rancaño, K. M. (2025). The Full Range of Weight Status by Race and Ethnicity in Children with and without Autism in the United States : A Cross-Sectional Study. The Journal of Pediatrics, 280, 114482. https://doi.org/10.1016/j.jpeds.2025.114482 |
| **Mari-Bauset 2015** | |
|  | Marí-Bauset, S., Llopis-González, A., Zazpe, I., Mari-Sanchis, A., & Morales-Suárez-Varela, M. (2015). Anthropometric measures of Spanish children with autism spectrum disorder. Research in Autism Spectrum Disorders, 9, 26–33. https://doi.org/10.1016/j.rasd.2014.09.013 |
|  | Marí-Bauset, S., Llopis-González, A., Zazpe, I., Marí-Sanchis, A., & Morales Suárez-Varela, M. (2017). Comparison of nutritional status between children with autism spectrum disorder and typically developing children in the Mediterranean Region (Valencia, Spain). Autism, 21(3), 310–322. https://doi.org/10.1177/1362361316636976 |
|  | Marí-Bauset, S., Llopis-González, A., Zazpe-García, I., Marí-Sanchis, A., & Morales-Suárez-Varela, M. (2015). Nutritional Status of Children with Autism Spectrum Disorders (ASDs): A Case–Control Study. Journal of Autism and Developmental Disorders, 45(1), 203–212. https://doi.org/10.1007/s10803-014-2205-8 |
|  | Bauset, S. M., Zazpe, I., Sanchis, A. M., González, A. L., & Suárez-Varela, M. M. (2013). Are There Anthropometric Differences Between Autistic and Healthy Children? Journal of Child Neurology, 28(10), 1226‑1232. https://doi.org/10.1177/0883073812458832 |
| **Levy 2019** | |
|  | Levy, S. E., Pinto-Martin, J. A., Bradley, C. B., Chittams, J., Johnson, S. L., Pandey, J., Pomykacz, A., Ramirez, A., Reynolds, A., Rubenstein, E., Schieve, L. A., Shapira, S. K., Thompson, A., Young, L., & Kral, T. V. E. (2019). Relationship of Weight Outcomes, Co-Occurring Conditions, and Severity of Autism Spectrum Disorder in the Study to Explore Early Development. The Journal of Pediatrics, 205, 202‑209. https://doi.org/10.1016/j.jpeds.2018.09.003 |
|  | Kral, T. V., Chittams, J., Bradley, C. B., Daniels, J. L., DiGuiseppi, C. G., Johnson, S. L., Pandey, J., Pinto-Martin, J. A., Rahai, N., Ramirez, A., Schieve, L. A., Thompson, A., Windham, G., York, W., Young, L., & Levy, S. E. (2019). Early life influences on child weight outcomes in the Study to Explore Early Development. Autism, 23(4), 954‑962. https://doi.org/10.1177/1362361318791545 |
| **McCoy 2020** | |
|  | Healy, S., Aigner, C. J., & Haegele, J. A. (2019). Prevalence of overweight and obesity among US youth with autism spectrum disorder. Autism, 23(4), 1046–1050. https://doi.org/10.1177/1362361318791817 |
|  | McCoy, S. M., & Morgan, K. (2020). Obesity, physical activity, and sedentary behaviors in adolescents with autism spectrum disorder compared with typically developing peers. Autism, 24(2), 387–399. https://doi.org/10.1177/1362361319861579 |
|  | Must, A., Eliasziw, M., Bandini, L. G., Curtin, C., Magaña, S., & Rancaño, K. M. (2025). The Full Range of Weight Status by Race and Ethnicity in Children with and without Autism in the United States : A Cross-Sectional Study. The Journal of Pediatrics, 280, 114482. https://doi.org/10.1016/j.jpeds.2025.114482 |
|  | Tybor, D. J., Eliasziw, M., Kral, T. V. E., Segal, M., Sherwood, N. E., Sikich, L., Stanish, H., Bandini, L., Curtin, C., & Must, A. (2019). Parental concern regarding obesity in children with autism spectrum disorder in the United States : National Survey of Children’s Health 2016. Disability and Health Journal, 12(1), 126‑130. https://doi.org/10.1016/j.dhjo.2018.09.004 |
| **McCoy 2016** | |
|  | Corvey, K., Menear, K. S., Preskitt, J., Goldfarb, S., & Menachemi, N. (2016). Obesity, Physical Activity and Sedentary Behaviors in Children with an Autism Spectrum Disorder. Maternal and Child Health Journal, 20(2), 466‑476. https://doi.org/10.1007/s10995-015-1844-5 |
|  | Dreyer Gillette, M. L., Borner, K. B., Nadler, C. B., Poppert, K. M., Odar Stough, C., Swinburne Romine, R., & Davis, A. M. (2015). Prevalence and Health Correlates of Overweight and Obesity in Children with Autism Spectrum Disorder. Journal of Developmental & Behavioral Pediatrics, 36(7), 489–496. https://doi.org/10.1097/DBP.0000000000000198 |
|  | McCoy, S. M., Jakicic, J. M., & Gibbs, B. B. (2016). Comparison of Obesity, Physical Activity, and Sedentary Behaviors Between Adolescents With Autism Spectrum Disorders and Without. Journal of Autism and Developmental Disorders, 46(7), 2317‑2326. https://doi.org/10.1007/s10803-016-2762-0 |
|  | Must, A., Eliasziw, M., Phillips, S. M., Curtin, C., Kral, T. V. E., Segal, M., Sherwood, N. E., Sikich, L., Stanish, H. I., & Bandini, L. G. (2017). The Effect of Age on the Prevalence of Obesity among US Youth with Autism Spectrum Disorder. Childhood Obesity, 13(1), 25‑35. https://doi.org/10.1089/chi.2016.0079 |
|  | Voulgarakis, H., Bendell-Estroff, D., & Field, T. (2017). Prevalence of obesity and autism spectrum disorder. Behavioral Development Bulletin, 22(1), 209‑214. https://doi.org/10.1037/bdb0000054 |
| **Mendive 2022** | |
|  | Mendive Dubourdieu, P., & Guerendiain, M. (2022). Dietary Intake, Nutritional Status and Sensory Profile in Children with Autism Spectrum Disorder and Typical Development. Nutrients, 14(10), 2155. https://doi.org/10.3390/nu14102155 |
|  | Mendive Dubourdieu, P., & Guerendiain, M. (2023). Understanding the link between gut microbiota, dietary intake, and nutritional status in children with autism and typical development. Frontiers in Nutrition, 10, 1202948. https://doi.org/10.3389/fnut.2023.1202948 |
| **Mehra 2024** | |
|  | Mehra, S., Salinas-Miranda, A. A., Buro, A. W., Marshall, J., & Kirby, R. S. (2024). The role of adverse childhood experiences in obesity among adolescents with autism spectrum disorder : National survey of Children’s health 2018-2019. Disability and Health Journal, 17(2), 101550. https://doi.org/10.1016/j.dhjo.2023.101550 |
|  | Must, A., Eliasziw, M., Bandini, L. G., Curtin, C., Magaña, S., & Rancaño, K. M. (2025). The Full Range of Weight Status by Race and Ethnicity in Children with and without Autism in the United States : A Cross-Sectional Study. The Journal of Pediatrics, 280, 114482. https://doi.org/10.1016/j.jpeds.2025.114482 |
| **Must 2023** | |
|  | Must, A., Eliasziw, M., Stanish, H., Curtin, C., Bandini, L. G., & Bowling, A. (2023). Passive and social screen time in children with autism and in association with obesity. Frontiers in Pediatrics, 11, 1198033. https://doi.org/10.3389/fped.2023.1198033 |
|  | Must, A., Eliasziw, M., Bandini, L. G., Curtin, C., Magaña, S., & Rancaño, K. M. (2025). The Full Range of Weight Status by Race and Ethnicity in Children with and without Autism in the United States : A Cross-Sectional Study. The Journal of Pediatrics, 280, 114482. https://doi.org/10.1016/j.jpeds.2025.114482 |

Table S6 Data issues found in studies during data extraction

| **Study** | **Description of issues found** |
| --- | --- |
| Hammouda 2018 (see table on Excluded studies with reasons for full bibliographic details). | In Figure 1 of this article, the bars do not match the sample size reported in the text. In the text, the total sample size indicated is 30 subjects, but in Figure 1, when the number of subjects for each category is summed (underweight, normal weight, and obese), there are more than 30 subjects.  Additionally, it is unclear whether the term "obesity" used by the authors refers to both obesity and overweight or to obesity alone.  Authors were contacted twice without response so the article was finally excluded. |

Table S7 Additional details on the overall samples of participants

Age data typically refers to the age of measurement of BMI

| **Author year** | **Other age data (years)** | **Socio-economic status** | **Ethnicity** | **Operational definition of obesity** | **Operational definition of overweight** | **Operational definition of low weight** |
| --- | --- | --- | --- | --- | --- | --- |
| Ahlberg 2022 |  |  |  | Other/Unclear | Category not reported | Category not reported |
| Al-Kindi 2016 | Range: 4-13 |  |  | >pc95 | >pc85 | <pc5 |
| Alkhalidy 2021 | Range: 3-6 |  |  | >pc95 | >pc85 | <pc5 |
| Ames 2022 | >18 | Insurance payer: Autistic group: 75.1% private, 24.9% government; Non-autistic group: 98.6% private, 1.4% government | White: 45.94%, Hispanic: 17.11%, Black: 7.22%, Asian: 15.77%, Other: 13.96% | Other/Unclear | Category not reported | Category not reported |
| Amin 2022 | Range: 5-12 |  |  | >pc95 | >pc85 | <pc5 |
| Arija 2023 | Range: 3-6&10-12 |  |  | >Z2 | >Z1.5 | Category not reported |
| Bandini 2013 |  |  | White 79.34% | >pc95 | >pc85 | <pc5 |
| Barnhill 2017 |  |  |  | >pc95 | >pc85 | <pc5 |
| Bener 2014 | Range: 3-8 | Monthly income ($ USD): $3,000: 31.50%, $3,000-$6,000: 38.39%, >$6,000: 30.12% | Qatari : autism children 35.0%, control children 47.2%; Non‑Qatari/Arabs autism children 65.0%, control children 52.8% | >pc95 | >pc85 | Category not reported |
| Błazewicz 2020 |  |  |  | >Z2 | >Z1.5 | <Z2 |
| Broder-Fingert 2014 | Range: 2-20 | Autism group: 55.1% had public insurance and 44.9% had private insurance; Asperger syndrome group: 44.8% had public insurance and 55.2% had private insurance; Controls: 53.0% had public insurance and 47.0% had private insurance. | White 64.33%, Black 11.2%, Hispanic 16.6%, Other 7.8% | >pc95 | >pc85 | Category not reported |
| Buro 2022 | Range: 10-17 | 0-99% Federal Poverty Level (FPL): 11.7; 100-199% FPL: 15.67; 200-399% FPL: 29.69; 400% FPL or greater: 42.85 | Hispanic 11.43%, White 69.7%, Black 6.78%, Multiracial/other 12.07% | Other/Unclear | Category not reported | Category not reported |
| Castro 2016 |  |  |  | >Z2 | >Z1.5 | <Z2 |
| Chen 2016 |  |  |  | Other/Unclear | Category not reported | Category not reported |
| Coppola 2024 | Range: 1.5-7 |  | Caucasian | >Z2 | >Z1.5 | <Z2 |
| Corbett 2021 | Range: 10-13 |  | 7.9% African-American, 83.0% Caucasian, 0.4% Asian, and 8.7% Mixed | >pc95 | >pc85 | <pc5 |
| Criado 2018 | Range: 4-17 | Parent’s highest education: Less than ninth grade: 0.98%, Some high school: 4.02%, High school or GED equivalent: 15.85%, Some college: 30.61%, College degree or higher: 48.54% | Non-Hispanic White: 70.37%, Non-Hispanic African American: 10.98%, Hispanic: 8.54%, Other: 10.12% | >pc95 | >pc85 | Category not reported |
| Curtin 2010 | Range: 3-17 |  |  | >pc95 | Category not reported | Category not reported |
| Davignon 2018 | Range: 14-25 |  | White, non-Hispanic 9710  White, Hispanic 1341  African American 2272  Asian 4559  Other 6856 | Other/Unclear | Other/Unclear | Category not reported |
| Eliasziw 2021 |  | Household income ≤$40,000: 40.2%, Household income $40,001 to $100,000: 38.2%, Household income ≥$100,000: 21.6%. | White 52.3%, Black 12.5%, Hispanic 25.1%, and Other 10.0%. | >pc95 | >pc85 | Category not reported |
| Esposito 2019 |  |  |  | >pc95 | >pc85 | <pc5 |
| Esteban-Figuerola 2021 | |  |  | >Z2 | >Z1.5 | <Z2 |
| FlygareWallen 2018 | Range: 0-85 |  |  | Other/Unclear | Category not reported | Category not reported |
| Fuentes-Albero 2024 |  |  |  | >pc95 | >pc85 | <pc5 |
| Garcia 2023 | Range: 10–17 | Below FLP (Total: 4530940) At or above FLP (Total: 22439488) | White (Total: 13614798) Others (Total: 14030937) | >pc95 | >pc85 | <pc5 |
| Hand 2020 | Range: 65 or older (65 - >84) |  | White: 84.08%, Black: 7.46%, Hispanic: 1.61%, Other or unknown: 6.94% | Other/Unclear | Category not reported | Category not reported |
| Healy 2017 |  |  |  | Other/Unclear | Other/Unclear | Category not reported |
| Herndon 2009 |  |  |  | >pc95 | >pc85 | <pc5 |
| Hill 2015 | Range: 2-17 |  |  | >pc95 | >pc85 | <pc5 |
| Hyman 2012 | Range: 2-11 |  |  | >pc95 | >pc85 | <pc5 |
| Islam 2024 | Range: 4-14 | Mother’s education: Below secondary: 36.1%; Secondary/higher secondary: 27.0%; Bachelor and above: 36.9%; Father’s education: Below secondary: 33.7%; Secondary/higher secondary: 18.3%; Bachelor and above: 48.0%. Mother’s occupation: Services: 5.5%; Housewife: 84.3%; Others: 10.2%. Father’s occupation: services: 42.2%; Business: 33.1%; others: 24.7%. Monthly family income: < 40,000: 34.0 %; 40,000–70,000: 34.6%; > 70,000: 31.4% |  | >Z2 | >Z1.5 | <Z2 |
| Kerekes 2015 | Range: 9-12 |  |  | >Z2 | >Z1.5 | <Z2 |
| Kral 2015 | Range: 4-6 |  |  | Category not reported | >pc85 | Category not reported |
| Kummer 2016 |  |  |  | >pc95 | >pc85 | <pc5 |
| Levy 2019 |  |  |  | >pc95 | >pc85 | <pc5 |
| Liu 2016 |  |  | Above 95% Han nationality | >Z2 | Category not reported | <Z2 |
| Mari-Bauset 2015 |  |  |  | >pc95 | >pc85 | <pc5 |
| Mathew 2022 | Mostly children |  |  | >pc95 | >pc85 | <pc5 |
| Mayes 2024 | Range: 5-17 ASD group & 6-12 control group |  |  | Category not reported | Other/Unclear | Other/Unclear |
| McCoy 2016 |  |  | White 76.23% | >pc95 | >pc85 | <pc5 |
| McCoy 2020 |  |  | White 76.25% | >pc95 | >pc85 | <pc5 |
| Mehra 2024 | Range: 10-17 | 0-99% Federal Poverty Level (FPL): 11.07; 100-199% FPL: 16.28, 200-399% FPL: 30.79, 400% FPL or greater: 41.88 | Hispanic 11.64%, White 69.93%, Black 6.45%, Multiracial/other 11.75% | Category not reported | >pc85 | Category not reported |
| Mendive 2022 | Range: 3-12 |  |  | >Z2 | >Z1.5 | Category not reported |
| Mills 2007 |  | Mother´s education: Some high school: 1.54%, High school graduate: 18.46%, Some college: 28.46%, College graduate or higher: 48.46% | White 90%, Black 5.38%, Asian .77 %, Other 3.4% | >pc95 | >pc85 | Category not reported |
| MMetwally 2024 |  |  |  | >Z2 | >Z1.5 | <Z2 |
| Molina-Lopez 2021 |  |  |  | >pc95 | >pc85 | <pc5 |
| Must 2023a | Range: 4-17 | Annual household income: <$50,000: 13%, $50,000–$100,000: 39.8%, >$100,000: 42.7% | White, non-Hispanic 75.1%, Other non-Hispanic 13.6% Hispanic 11.3% | >pc95 | Category not reported | Category not reported |
| Must 2023b | Range: 9-10 | SES Low: 12.4%, Medium: 26.9%, High: 60.7% | White (53.8%), with 15.6% being Hispanic, 13.4% Black, and 1.6% Asian. | >pc95 | Category not reported | Category not reported |
| Phillips 2014 | Range: 12-17 |  |  | >pc95 | >pc85 | <pc5 |
| Raspini 2021 |  |  |  | >pc95 | >pc85 | <pc5 |
| Riccio 2020 | Range: 2-11 | 100 % Middle socioeconomic status | All subjects were recruited from the same geographic area (Mediterranean coast of Campania region—Southern Italy) and were Caucasian from middle socioeconomic status. | >pc95 | >pc85 | <pc5 |
| Rimmer 2010 | Range: 12-17 |  |  | >pc95 | >pc85 | <pc5 |
| Rouphael 2024 | Range: 3-18 |  |  | >Z2 | >Z1.5 | <Z2 |
| Schott 2022 | Range: 18-64 |  | White: 62.6%  Black: 17.3%  Asian/Pacific Islander: 2.5%  Hispanic/Latino: 8.2%  Other/Missing: 9.4% | Other/Unclear | Category not reported | Category not reported |
| Sedgewick 2020 | Range: 18-81 |  | Autistic: white 73.43%, Asian 2.38%, Black 0.9%, Latin 0.9%, Mixed 4.18%, No answer 17.61%. Non-autistic: White 262%, Asian 7.27%, Black 0.91%, Latin 0.3 %, Mixed 2.73%, No answer 9.39% | Other/Unclear | Other/Unclear | Other/Unclear |
| Shea 2024 |  |  |  | Other/Unclear | Category not reported | Category not reported |
| Shedlock 2016 | Range: 2-17 |  |  | >pc95 | Category not reported | Category not reported |
| Trinh 2023 | >2 |  |  | >Z2 | Category not reported | <Z2 |
| Tyler 2011 |  |  | More than 75% Caucasian | >Z2 | >Z1.5 | <Z2 |
| Wei 2018 | Range: 2-5 |  |  | Other/Unclear | Category not reported | Other/Unclear |
| Weir 2021 |  |  | Predominantly composed of white individuals | >Z2 | >Z1.5 | <Z2 |
| Zhu 2020 | Range: 2-6 |  | Han (95.29%) & Others (4.71%) | >Z2 | Category not reported | Category not reported |
| Zimmer 2012 |  |  |  | Category not reported | Category not reported | <pc5 |
| Zurita 2020 |  |  |  | >Z2 | >Z1.5 | <Z2 |

Table S8 Description of the ASD group in studies

Age data typically refers to the age of measurement of BMI

| **Author year** | **Mean age** | **Other age data (years)** | **Gender (% M)** | **Socio-economic status** | **Ethnicity** |
| --- | --- | --- | --- | --- | --- |
| Ahlberg 2022 | 16.26 |  | 64.95 |  |  |
| Al-Kindi 2016 |  |  | 79.1 | Monthly income level: <500: 25.8%; 500-1000: 47.9%; 1000-1500: 11.7%; >1500: 14.7% |  |
| Alkhalidy 2021 | 4.4 |  | 71.2 |  |  |
| Ames 2022 |  | >18 | 73.1 |  | White 65.6%; Hispanic 9.5%; Non-hispanic black 7.4%; Asian 10.6%; Other race/ethnicity 7% |
| Amin 2022 | 8.77 |  | 73.3 |  |  |
| Arija 2023 | 8.12 | Range: 3-6&10-12 | 81.2 |  |  |
| Bandini 2013 | 6.6 |  | 83 |  | White 83% |
| Barnhill 2017 | 5.52 |  | 92 |  |  |
| Bener 2014 | 5.51 | Range: 3-8 | 64.9 | Monthly income ($ USD): <$3,000 (28.0); $3,000-$6,000 (39.4); >$6,000 (32.7) | Qatari: 35.0% Non-Qatari/Arabs: 65.0% |
| Błazewicz 2020 | 7.97 |  | 83.55 |  |  |
| Broder-Fingert 2014 | 11.47 | Range: 2-20 | 79.00 |  | White 80.74%; Black 4.93%; Hispanic 7.09%; Other 7.02% |
| Buro 2022 | 13.73 | Range: 10-17 | 75.3 | 0-99% Federal Poverty Level (FPL): 23.8; 100-199% FPL: 30.6; 200-399% FPL: 22.4; 400% FPL or greater: 23.2 | Hispanic 33.7%; White, non-Hispanic 47% ; Black, non-Hispanic 12.6%; Multi-racial/Other, non-Hispanic: 6.8% |
| Castro 2016 | 10.5 |  | 100 |  |  |
| Chen 2016 | 15.25 |  | 80 |  |  |
| Coppola 2024 | 4.16 |  | 79 | Family socio-economic status: low (19), medium (59), high (22) | Caucasian |
| Corbett 2021 | 11.42 |  | 74 | M(SD): 15.68 (2.98) |  |
| Criado 2018 |  | Median 7.5; IQR: 5.83-9.42; Range: 4-17 | 84.4 |  | Non hispanic caucasian 69.9%; Non hispanic african american 10.9%; Hispanic 8.7%; Other 10.7% |
| Curtin 2010 |  | Range: 3-17 | 79 | Poverty to income ratio <1 18.1 (4.70) 1 - <2 21.9 (3.29) 2 - <4 32.9 (3.57) >4 27.0 (3.05) | Hispanic: 10.0 (3.18) Non-Hispanic White: 70.4 (4.34) Non-Hispanic Black: 15.5 (3.86) Multi-racial: 1.5 (0.55) Other: 2.6 (0.93) |
| Davignon 2018 | 18.4 | Range: 14-25 | 80.67 |  | White, non-Hispanic 2208 (53.55)  White, Hispanic 286 (6.94) African American 339 (8.22) Asian 649 (15.74) Other 641 (15.55) |
| Eliasziw 2021 | 6.03 |  | 80.7 | 27.1% lived in households with incomes of $40 000 to 100 000, 31.8% >100000$, <40 000$ 41.1% | 59.2% White race, 6.4% Black, 22% hispanic, 12.4% others |
| Esposito 2019 | 4.5 | Range: 2-8 | 84 |  |  |
| Esteban-Figuerola 2021 | 8.23 |  | 83.54 |  | Autochthon: preschool age children 84.2%, school age children 87.8% |
| FlygareWallen 2018 |  | Range: 0-85 | 65.63 |  |  |
| Fuentes-Albero 2024 | 9.95 |  | 80 |  |  |
| Garcia 2023 | 13.55 | Range: 10-17 | 80.22 | Below Family Poverty Level (urban: 20.08%, rural: 36.22%) At or above FPL (urban: 79.92%, rural: 63.78%) | White (urban: 48.96%, rural: 26.30%). Others (urban: 51.04%, rural: 73.7%) |
| Hand 2020 | 71.2 | Range: 65-84 | 67.77 | Household income median, thousands in $ (IQR): 31, (10-83) | White 88.9% Black 7.1% Hispanic 0.7% Other or unknown 3.3% |
| Healy 2017 | 13 |  | 79.1 | 18,507 Household annual income |  |
| Herndon 2009 | 4.66 | Range: 3-8 | 95.7 |  | Caucasian 71.7%, Hispanic 13%, African American 4.3%, Asian 2.2%, Bi-racial 2.2%, Unknown 6.5% |
| Hill 2015 |  |  | 84.5 |  | Hispanic (9,77%), non-hispanic (85,98%), missing (4,23%) |
| Hyman 2012 | 5.37 |  | 86 |  |  |
| Islam 2024 | 8 | Range: 4-14 | 70.4 | Mother’s education: Below secondary 5.8%; Secondary/higher secondary 26.7%; Bachelor and above 67.4%; Father’s education: Below secondary 4.7%; Secondary/higher secondary 11.6%; Bachelor and above 83.7%; Mother’s occupation: Services 5.2%; Housewife 83.1%; Others 11.6%; Father’s occupation: Services 46.5%; Business 27.9%; Others 25.6%; Monthly family income (BDT): < 40,000 8.7%; 40,000–70,000 38.4%; > 70,000 52.9%. |  |
| Kerekes 2015 | 10.81 |  | 73.18 |  |  |
| Kral 2015 | 5 | Range: 4-6 | 72 |  | Hispanic 16%, Non-hispanic 84% |
| Kummer 2016 | 8.4 |  | 86.9 |  |  |
| Levy 2019 | 59.3 |  | 81.6 | Below federal poverty level: 10.7% | White 61.1%, Black 18.4%, Asian 8.7%, American Indian or Pacific islander 0.8%, Multiracial 4%, Hispanic 3.1% |
| Liu 2016 | 5.21 | IQR: 3.85, 6.33 | 91.6 |  |  |
| Mari-Bauset 2015 | 7.81 |  | 87 |  |  |
| Mathew 2022 | 7.6 | Range: 2–5 (32.5%); 6–12 (60.4%); ≥13 (7.1%) | 79 |  |  |
| Mayes 2024 | 8.1 | Range: 5-17 | 79.4 | One or both parents have a professional or managerial occupation: 35.1% | White 92.1% |
| McCoy 2016 | 13.32 | Range: 10-17 | 81 | Household income: poor 21.1%, near poor 8.6%, not poor 62.5%, missing 7.8% | White 74% |
| McCoy 2020 | 13.7 |  | 80.4 | Poor 18.1%, near poor 8.9%, not poor 73% | White 73.6% |
| Mehra 2024 |  | Range: 10-17 | 79.3 | 0-99% Federal Poverty Level (FPL): 23.5; 100-199% FPL: 27.1; 200-399% FPL: 27; 400% FPL or greater: 22.3 | Hispanic 28.1%; White, non-Hispanic: 51%; Black, non-Hispanic 13.5%; Multi-racial/Other, non-hispanic: 7.3% |
| Mendive 2022 | 5.83 |  |  |  |  |
| Mills 2007 | 6.6 |  | 100 | Mother´s education: Some high school: 1 (1.5%), High school graduate: 13 (19.1%), Some college: 20 (29.4%), College graduate or higher: 34 (50%) | White 88%, Black 6.7%, Asian 1.4%, Other 5.3% |
| MMetwally 2024 | 6.6 |  |  | Social class: low (25.6%), middle (36.5%), high (37.9%) |  |
| Molina-Lopez 2021 | 9.57 |  |  |  |  |
| Must 2023a | 9.2 | Range: 4-17 | 86.6 | Annual household income: <$50,000: 13%, $50,000–$100,000: 39.8%, >$100,000: 42.7% | White, non-Hispanic 75.1%, Other non-Hispanic 13.6% Hispanic 11.3% |
| Must 2023b | 9.55 | Range: 9-10 | 85.5 | SES Low: 12.4%, Medium: 26.9%, High: 60.7% | Hispanic 15.6% White, non-Hispanic 53.8% Black, non-Hispanic 13.4% Asian, non-Hispanic 1.6% Other or Multi, non-Hispanic 15.6 |
| Phillips 2014 |  | Range: 12-17 | 79.7 |  | Non-Hispanic White 71.3%, Non-Hispanic Black 13%, American Indian/ Native American 0%, Asian 2.5%, Other 4.6%, Hispanic 8.6% |
| Raspini 2021 | 3.6 |  | 87.7 |  |  |
| Riccio 2020 | 3.94 | Range: 2-11 | 70.2 | 100% Middle socioeconomic status | Caucasian 100% |
| Rimmer 2010 | 14.7 | Range: 12-18 |  |  |  |
| Rouphael 2024 | 10.4 |  | 86 |  |  |
| Schott 2022 | 28.48 | Range: 18-64 | 74.15 |  | White 62.66 Black 16.95 Asian/Pacific Islander 2.45 Hispanic/Latino 7.93 Other 10.01 |
| Sedgewick 2020 | 34.06 | Range: 18-71 | 15.82 |  | White 73.43%, Asian 2.38%, Black 0.9%, Latin 0.9%, Mixed 4.18%, No answer 17.61%. |
| Shea 2024 | 23 |  | 0 | Medicaid eligibility group. Poverty: 23.3%, disablity: 42.1%, Other: 34.6% | American Indian and Alaskan native: 1.3%; Asian or other pacific islander: 1.5%, Black: 24.7%; Hispanic or Latino: 10.1; Multiracial: 0.6; White: 57.2%; Missing: 4.6%. |
| Shedlock 2016 |  | Range: 2-17 Median: 10.42 IQR: 7.56-13.43 | 78.55 |  |  |
| Trinh 2023 |  | >2 | 69.9 |  |  |
| Tyler 2011 | 28.8 |  | 71.3 |  | Caucasian 76.9%  African American 13.9%  Hispanic 1.9 %  Other 7.4% |
| Wei 2018 |  | Range: 2-5 | 90.36 |  |  |
| Weir 2021 | 41.04 |  | 36.94 |  | White 88.33%  Non-White 11.42% Mixed Race 6.51%  Asian 1.52%   Latin American/Hispanic 0.59%  Arab/Middle Eastern 0%  Jewish 1.35%  African/Black/Caribbean 0.51%  Other 0.93%  Missing 0.25% |
| Zhu 2020 | 4.34 | Range: 2-6 | 85.23 |  | Han (94.99) & Others (5.01) |
| Zimmer 2012 | 8.2 |  | 91 |  | Caucasian 95%, Black 5% |
| Zurita 2020 | 8.92 |  | 96 | Family income: $1500 or more 4%, $700 to $1499 72%, $200 to $600 24% | Mestizo 100% |

Table S9 Description of the Non-ASD group in studies

Age data typically refers to the age of measurement of BMI

| **Author year** | **Mean age (year)** | **Other age data** | **Gender (% M)** | **Socio-economic status** | **Ethnicity** |
| --- | --- | --- | --- | --- | --- |
| Ahlberg 2022 | 21.22 |  | 51.77 |  |  |
| Al-Kindi 2016 |  |  | 49.1 | Monthly income level: <500: 29.7%, 500-1000: 50.5%, 1000-1500: 13.2%, >1500: 6.6% |  |
| Alkhalidy 2021 | 4.18 |  | 51 |  |  |
| Ames 2022 |  | >18 | 73.1 |  | White 44% Hispanic 17.9% Non-hispanic black 7.2% Asian 16.3% Other race/ethnicity 14.7% |
| Amin 2022 | 9.42 |  | 46.7 |  |  |
| Arija 2023 | 8.51 | Range: 3-6&10-12 | 56.72 |  |  |
| Bandini 2013 | 6.7 |  | 78 |  | White 76% |
| Barnhill 2017 | 6.2 |  | 82 |  |  |
| Bener 2014 | 5.76 | Range: 3-8 | 56.7 | Monthly income ($ USD): <$3,000 (35.0); $3,000-$6,000 (37.4); >$6,000 (27.6) | Qatari 47.2%; Non-Qatari/Arabs 52.8% |
| Błazewicz 2020 | 8.33 |  | 58 |  |  |
| Broder-Fingert 2014 | 11.09 | Range: 2-20 | 50.1 |  | White 51.1%, Black 16.3%, Hispanic 24.3%, other 8.4% |
| Buro 2022 | 13.81 | Range: 10-17 | 50.3 | 0-99% Federal Poverty Level (FPL): 20, 100-199% FPL: 21.5, 200-399% FPL: 27, 400% FPL or greater: 31.5 | Hispanic: 25.6%;  White, non-Hispanic: 49.7%;  Black, non-Hispanic: 14.8%;  Multiracial/other, non-Hispanic: 9.9%  Other or Multi, non-Hispanic 15.6 |
| Castro 2016 | 10.02 |  |  |  |  |
| Chen 2016 | 15.25 |  | 80 |  |  |
| Coppola 2024 | 4.15 | 49.8 months | 75.2 | Family socio-economic status: low (22), medium (58), high (20) | Caucasian |
| Corbett 2021 | 11.73 |  | 55.3 | M(SD): 17.34 (2.17) |  |
| Criado 2018 |  | Median 7.5, IQR: 5.79-9.42. Range: 4-17 | 84.4 |  | Non-Hispanic white (70.6%), Non-Hispanic African american (11%), Hispanic (8.5%), Other (9.9%) |
| Curtin 2010 |  | Range: 3-17 | 51 | Poverty to income ratio <1 17.0 (0.28)  1 - <2 22.7 (0.28)  2 - <4 33.4 (0.29)  >4 26.8 (0.26) | Hispanic: 16.9 (0.25) Non-Hispanic White: 61.4 (0.31) Non-Hispanic Black: 14.6 (0.23) Multi-racial: 2.9 (0.10) Other: 4.2 (0.18) |
| Davignon 2018 |  | Range: 14-25 | 80.67 |  | White, non-Hispanic 7502 (36.39) White, Hispanic 1055 (5.12) African American 1933 (9.38) Asian 3910 (18.97) Other 6215 (30.15) |
| Eliasziw 2021 | 6.1 |  | 51.1 | incomes <40 000$: 40.2%, from 40 000 to 100 000$: 38.2%; > 100 000$: 21.6% | White 52.3%, Black 12.6%, Hispanic 25.1%, Others 10% |
| Esposito 2019 | 4.67 | Range 2-8 | 78 |  |  |
| Esteban-Figuerola 2021 | 8.55 |  | 57.10 |  | Autochthon: preschool age children 74.1%, school age children 86.3% |
| FlygareWallen 2018 |  | Range: 0->85 | 49.29 |  |  |
| Fuentes-Albero 2024 | 9.85 |  | 80 |  |  |
| Garcia 2023 | 13.49 | Range: 10-17 | 50.12 | Below FPL (urban: 18.11, rural: 23.17) At or above FPL (Urban: 81.89%, Rural: 76.83%) | White (urban: 46.36%, rural: 71.07%) Others (urban: 53.64%, rural: 28.93%) |
| Hand 2020 | 71.2 | Range: 65 - >84 | 67.77 | Household income median, thousands in $ (IQR): 28, (8-77) | White 83.6% Black 7.5%  Hispanic 1.7%  Other 7.3% |
| Healy 2017 | 13 |  | 52.7 | 16.87 Household annual income |  |
| Herndon 2009 | 4.99 | Range: 2-7 | 74.2 |  | Caucasian 83.9%, Hispanic 6.5%, African American 0%, Asian 0%, Bi-racial 6.5%, Unknown 3.2% |
| Hill 2015 |  |  |  |  |  |
| Hyman 2012 |  |  |  |  |  |
| Islam 2024 | 7.8 | Range: 4-14 | 70.4 | Mother’s education: Below secondary 66.3%; Secondary/higher secondary 27.3%; Bachelor and above 6.4%; Father’s education: Below secondary 62.8%; Secondary/higher secondary 25.0%; Bachelor and above 12.2%; Mother’s occupation: Services 5.8%; Housewife 85.5%; Others 8.7%; Father’s occupation: Services 37.8%; Business 38.4%; Others 23.8%; Monthly family income (BDT): < 40,000 59.3%; 40,000–70,000 30.8%; > 70,000 9.9%. |  |
| Kerekes 2015 | 10.56 |  |  |  |  |
| Kral 2015 | 5.2 | Range: 4-6 | 47 |  | Hispanic 3%, Non-Hispanic 97% |
| Kummer 2016 | 8.6 |  |  |  |  |
| Levy 2019 | 59.2 |  | 52.3 | Below federal poverty level: 5,7% | White 73.1%, Black 10.6%, Asian 4.4%, American Indian or pacific islander 0.6%, multiracial 4.1%, Hispanic 2.2% |
| Liu 2016 | 4.83 | IQR: 4.41, 5.75 | 91.8 |  |  |
| Mari-Bauset 2015 | 7.96 |  | 54 |  |  |
| Mathew 2022 | 7.74 | Range 2–5: siblings (38.0%), not relatives (39.4%); range 6–12: siblings (47.9%), not relatives (43.7%); range ≥13: siblings (14.1%), not relatives (16.9%) | 50.5 |  |  |
| Mayes 2024 | 8.7 | Range: 6-12 | 52.6 | One or both parents have a professional or managerial occupation: 48.9% | White 80.5% |
| McCoy 2016 | 13.65 | Range: 10-17 | 51.1 | Household income: poor 17.2%, near poor 6.7%, not poor 67.1%, missing 9% | White non-Hispanic 68% |
| McCoy 2020 | 13.8 |  | 49.9 | poor 13.4%, near poor 7.7 %, not poor 78.9% | White non-Hispanic: 0,714% |
| Mehra 2024 |  | Range: 10-17 | 48.6 | 0-99% Federal Poverty Level (FPL): 18.9; 100-199% FPL: 22.1; 200-399% FPL: 27.9; 400% FPL or greater: 31.1 | Hispanic: 26%;  White, non-Hispanic: 49.5%;  Black, non-Hispanic: 14.2%;  Multi-racial/other, non-Hispanic: 10.3% |
| Mendive 2022 | 7.19 |  |  |  |  |
| Mills 2007 | 6.5 |  | 100 | Mother´s education: Some high school: 1 (1.7%), High school graduate: 11 (19%), Some college: 17 (29.3%), College graduate or higher: 29 (50%) | White 93.2%, Black 3.4%, Asian 0%, Other 3.4% |
| MMetwally 2024 | 6,8 |  |  | Social class: low (30.8), middle (34.8), high (34.4) |  |
| Molina-Lopez 2021 | 12.8 |  |  |  |  |
| Must 2023a | 9.2 | Range: 4-17 | 46 | Annual household income: <$50,000: 13%, $50,000–$100,000: 39.8%, >$100,000: 42.7% | White, non-Hispanic 75.1%, Other non-Hispanic 13.6% Hispanic 11.3% |
| Must 2023b | 9.55 | Range: 9-10 | 85.5 | SES Low: 12.4%, Medium: 26.9%, High: 60.7% | Hispanic 15.6% White, non-Hispanic 53.8% Black, non-Hispanic 13.4% Asian, non-Hispanic 1.6% Other or Multi, non-Hispanic 15.6 |
| Phillips 2014 |  | Range: 12-17 | 47 |  | Non-Hispanic White 56.9%, Non-Hispanic Black 13.6%, American Indian/ Native American 0.6%, Asian 4.5%, Other 2.9%, Hispanic 21.5% |
| Raspini 2021 | 3.7 |  | 58.5 |  |  |
| Riccio 2020 | 5.44 | Range: 2-11 | 56.82 | 100% Middle socioeconomic status | Caucasian 100% |
| Rimmer 2010 |  | Range: 12-18 |  |  |  |
| Rouphael 2024 | 10.4 |  | 86 |  |  |
| Schott 2022 | 31.13 | Range: 18-64 | 74.08 |  | White 62.44% Black 18.45% Asian/Pacific Islander 2.51% Hispanic/Latino 8.40% Other 8.19% |
| Sedgewick 2020 | 32.67 | Range: 18-81 | 16.06 |  | White 79.39%, Asian 7.27%, Black 0.91%, Latin 0.3 %, Mixed 2.73%, No answer 9.39% |
| Shea 2024 | 26.4 |  | 0 | Medicaid eligibility group. Poverty: 56.1%, disability: 5.9%, Other: 38% | American Indian and Alaskan native: 1.5%; Asian or other pacific islander: 3.4%, Black: 22.8%; Hispanic or Latino: 29.1; Multiracial: 0.4%; White: 39.4%; Missing: 3.4%. |
| Shedlock 2016 |  | Range 2-17, Median: 10.24 IQR: 7.76-12.68 | 80.04 |  |  |
| Trinh 2023 |  | >2 | 61.3 |  |  |
| Tyler 2011 | 29.5 |  | 71.8 |  | Caucasian 78.6%  African American 14.1%  Hispanic 1.9%  Other 5.3% |
| Wei 2018 |  | Range: 2-5 | 90.36 |  |  |
| Weir 2021 | 41.86 |  | 31.42 |  | White 84.78% Non-White 15.21%  Mixed Race 6.07% Asian 3.57%  Latin American/Hispanic 1.91%  Arab/Middle Eastern 1.41% Jewish 1.41%  African/Black/Caribbean 0.75% Other 0.08% Missing 0% |
| Zhu 2020 | 4.4 | Range: 2-6 | 50.33 |  | Han (96.03%) & Others (3.97%) |
| Zimmer 2012 | 8.1 |  | 45 |  | Caucasian 77%, Black 23% |
| Zurita 2020 | 8.46 |  | 91.4 | Family income: $1500 or more 42.9%, $700 to $1499 42.9%, $200 to $600 14% | Mestizo 100% |

*Table S10 Risk of Bias rating (Newcastle-Ottawa scale)*

|  | **S&R1** | **S&R2** | **S&R3** | **S&R4** | **C1** | **O1** | **O2** | **O3** | **O4** | **O5** |
| --- | --- | --- | --- | --- | --- | --- | --- | --- | --- | --- |
|  | **Selection and Representativeness** | | | | **Comparability** | **Outcomes** | | | | |
|  | **Representativeness of the overall sample** | **Representativeness of the ASD group** | **Selection of the non-ASD group** | **Ascertainment of ASD** | **Comparability of the ASD and non ASD group** | **Sample size** | **Ascertainment of weight** | **Clear definition of obesity** | **Clear definition of overweight** | **Clear definition of underweight** |
| Ahlberg 2022 | 1 | 1 | 1 | 1 | 0 | 1 | 1 | 0 | NA | NA |
| Al-Kindi 2016 | 0 | 0 | 0 | 1 | 0 | 0 | 1 | 1 | 1 | 1 |
| Alkhalidy 2021 | 0 | 0 | 0 | 1 | 0 | 0 | 1 | 1 | 1 | 1 |
| Ames 2022 | 1 | 1 | 1 | 1 | 1 | 1 | 1 | 0 | NA | NA |
| Amin 2022 | 0 | 0 | 0 | 0 | 0 | 0 | 1 | 0 | 0 | 0 |
| Arija 2023 | 1 | 1 | 1 | 1 | 0 | 1 | 1 | 1 | 1 | NA |
| Bandini 2013 | 0 | 0 | 0 | 1 | 0 | 0 | 1 | NA | 1 | 1 |
| Barnhill 2017 | 0 | 0 | 0 | 1 | 1 | 0 | 1 | 1 | 1 | 1 |
| Bener 2014 | 0 | 0 | 0 | 1 | 1 | 1 | 1 | 1 | 1 | NA |
| Błazewicz 2020 | 0 | 0 | 0 | 1 | 0 | 1 | 1 | 1 | 1 | 1 |
| Broder-Fingert 2014 | 1 | 1 | 1 | 1 | 0 | 1 | 1 | 1 | 1 | NA |
| Buro 2022 | 1 | 1 | 1 | 0 | 0 | 1 | 0 | 0 | NA | NA |
| Castro 2016 | 0 | 0 | 1 | 1 | 1 | 0 | 1 | 1 | 1 | 1 |
| Chen 2016 | 1 | 1 | 1 | 1 | 1 | 1 | 1 | 0 | NA | NA |
| Coppola 2024 | 0 | 0 | 0 | 1 | 1 | 0 | 1 | 1 | 1 | 1 |
| Corbett 2021 | 0 | 0 | 1 | 1 | 0 | 0 | 1 | 1 | 1 | 1 |
| Criado 2018 | 0 | 0 | 0 | 1 | 1 | 1 | 1 | 1 | 1 | NA |
| Curtin 2010 | 1 | 1 | 1 | 0 | NA | 1 | 0 | 1 | NA | NA |
| Davignon 2018 | 1 | 1 | 1 | 1 | 0 | 1 | 1 | 0 | 0 | NA |
| Eliasziw 2021 | 1 | 1 | 1 | 0 | NA | 1 | 1 | 1 | 1 | NA |
| Esposito 2019 | 0 | 0 | 1 | 1 | 0 | 0 | 0 | 1 | 1 | 1 |
| Esteban-Figuerola 2021 | 0 | 0 | 1 | 1 | 0 | 1 | 1 | 1 | 1 | 1 |
| FlygareWallen 2018 | 1 | 1 | 1 | 1 | NA | 1 | 1 | 0 | NA | NA |
| Fuentes-Albero 2024 | 0 | 0 | 0 | 1 | 1 | 0 | 0 | 1 | 1 | 1 |
| Garcia 2023 | 1 | 1 | 1 | 0 | 1 | 1 | 0 | 1 | 1 | 1 |
| Hand 2020 | 1 | 1 | 1 | 1 | 1 | 1 | 1 | 0 | NA | NA |
| Healy 2017 | 1 | 1 | 1 | 0 | 0 | 0 | 1 | 0 | NA | NA |
| Herndon 2009 | 0 | 0 | 1 | 1 | 0 | 0 | 1 | 1 | 1 | 1 |
| Hill 2015 | 0 | 0 | 0 | 1 | 0 | 1 | 1 | 1 | 1 | NA |
| Hyman 2012 | 0 | 0 | 0 | 1 | 1 | 1 | 1 | 1 | 1 | 1 |
| Islam 2024 | 0 | 0 | 0 | 1 | 1 | 1 | 1 | 1 | 1 | 1 |
| Kerekes 2015 | 0 | 0 | 1 | 1 | NA | 1 | 0 | 1 | 1 | 1 |
| Kral 2015 | 0 | 0 | 0 | 1 | 1 | 1 | 1 | NA | 1 | NA |
| Kummer 2016 | 0 | 0 | 0 | 1 | 1 | 0 | 1 | 1 | 1 | 1 |
| Levy 2019 | 0 | 0 | 1 | 1 | 0 | 1 | 1 | 1 | 1 | 1 |
| Liu 2016 | 0 | 0 | 0 | 1 | 1 | 0 | 1 | 1 | NA | 1 |
| Mari-Bauset 2015 | 0 | 0 | 1 | 1 | 0 | 1 | 1 | 1 | 1 | 1 |
| Mathew 2022 | 0 | 0 | 1 | 1 | 1 | 0 | 0 | 1 | 1 | 1 |
| Mayes 2024 | 1 | 1 | 0 | 1 | NA | 1 | 0 | NA | 0 | 0 |
| McCoy 2016 | 1 | 1 | 1 | 0 | 0 | 1 | 0 | 1 | 1 | 1 |
| McCoy 2020 | 1 | 1 | 1 | 0 | 0 | 1 | 0 | 1 | 1 | 1 |
| Mehra 2024 | 1 | 1 | 1 | 0 | 0 | 1 | 0 | NA | 1 | NA |
| Mendive 2022 | 0 | 0 | 1 | 1 | 0 | 1 | 1 | 1 | 1 | NA |
| Mills 2007 | 0 | 0 | 1 | 1 | 1 | 0 | 1 | 1 | 1 | NA |
| MMetwally 2024 | 1 | 1 | 1 | 1 | 1 | 0 | 1 | 1 | 1 | 1 |
| Molina-Lopez 2021 | 0 | 0 | 1 | 1 | 0 | 0 | 1 | 1 | 1 | 1 |
| Must 2023-CO | 1 | 1 | 1 | 1 | 0 | 1 | 1 | 1 | NA | NA |
| Must 2023-FP | 1 | 0 | 1 | 0 | 1 | 1 | 1 | 1 | NA | NA |
| Phillips 2014 | 1 | 1 | 1 | 0 | 0 | 0 | 0 | 1 | 1 | 1 |
| Raspini 2021 | 0 | 0 | 0 | 1 | 0 | 0 | 1 | 1 | 1 | 1 |
| Riccio 2020 | 0 | 0 | 1 | 1 | 0 | 0 | 1 | 1 | 1 | 1 |
| Rimmer 2010 | 0 | 0 | 0 | 0 | 1 | 1 | 0 | 1 | 1 | 1 |
| Rouphael 2024 | 0 | 0 | 1 | 1 | 1 | 0 | 1 | 1 | 1 | 1 |
| Schott 2022 | 0 | 0 | 1 | 1 | 1 | 1 | 1 | 0 | NA | NA |
| Sedgewick 2020 | 0 | 0 | 1 | 0 | 0 | 1 | 0 | 0 | 0 | 0 |
| Shea 2024 | 0 | 0 | 1 | 1 | 1 | 1 | 1 | 0 | NA | NA |
| Shedlock 2016 | 0 | 0 | 1 | 1 | 1 | 1 | 1 | 1 | NA | NA |
| Trinh 2023 | 0 | 0 | 0 | 1 | 1 | 0 | 1 | 1 | NA | 1 |
| Tyler 2011 | 1 | 1 | 1 | 1 | 1 | 1 | 1 | 1 | 1 | 1 |
| Wei 2018 | 0 | 0 | 1 | 1 | 1 | 0 | 1 | 0 | NA | 0 |
| Weir 2021 | 0 | 0 | 1 | 0 | 0 | 1 | 0 | 1 | 1 | 1 |
| Zhu 2020 | 0 | 0 | 1 | 1 | 0 | 1 | 1 | 1 | NA | NA |
| Zimmer 2012 | 0 | 0 | 0 | 1 | 0 | 0 | 1 | NA | NA | 1 |
| Zurita 2020 | 0 | 0 | 1 | 1 | 1 | 0 | 1 | 1 | 1 | 1 |

Table S11 Outcomes: number of individuals per weight category in the group with and without ASD

a included in overweight

b included in normal weight

c Excluded in the study

|  | **ASD** | | | | **No ASD** | | | |
| --- | --- | --- | --- | --- | --- | --- | --- | --- |
| **Author year** | **Obesity** | **Overweight** | **Normal** | **Underweight** | **Obesity** | **Overweight** | **Normal** | **Underweight** |
| Ahlberg 2022 | 1954 | b | 35190 | b | 59866 | b | 3044695 | b |
| Al-Kindi 2016 | 3 | 22 | 91 | 47 | 2 | 17 | 126 | 67 |
| Alkhalidy 2021 | 11 | 3 | 33 | 5 | 5 | 8 | 37 | 1 |
| Ames 2022 | 509 | b | 998 | b | 4073 | b | 10997 | b |
| Amin 2022 | 28 | 7 | 24 | 1 | 28 | 14 | 54 | 24 |
| Arija 2023 | 22 | 29 | 66 | b | 47 | 81 | 205 | b |
| Bandini 2013 | A | 14 | 38 | 1 | a | 13 | 45 | 0 |
| Barnhill 2017 | 8 | 9 | 65 | 4 | 4 | 10 | 39 | 3 |
| Bener 2014 | 5 | 22 | 227 | b | 9 | 41 | 204 | b |
| Błazewicz 2020 | 84 | 18 | 24 | 26 | 23 | 30 | 69 | 13 |
| Broder-Fingert 2014 | 709 | 407 | 1860 | b | 233 | 403 | 3060 | b |
| Buro 2022 | 194 | b | 674 | b | 3242 | b | 21993 | b |
| Castro 2016 | 18 | 5 | 15 | 11 | 9 | 12 | 25 | 3 |
| Chen 2016 | 166 | b | 5956 | b | 273 | b | 24215 | b |
| Coppola 2024 | 24 | 11 | 65 | 0 | 8 | 11 | 77 | 4 |
| Corbett 2021 | 38 | 19 | 73 | 6 | 16 | 12 | 69 | 3 |
| Criado 2018 | 59 | 58 | 159 | b | 65 | 77 | 402 | b |
| Curtin 2010 | 138 | b | 316 | b | 19935 | b | 64534 | b |
| Davignon 2018 | 1037 | 784 | 2302 | b | 3164 | 3482 | 13969 | b |
| Eliasziw 2021 | 10 | 20 | 70 | c | 1280 | 1540 | 6830 | c |
| Esposito 2019 | 6 | 5 | 31 | 9 | 3 | 8 | 35 | 4 |
| Esteban-Figuerola 2021 | 18 | 18 | 35 | 8 | 49 | 89 | 166 | 48 |
| FlygareWallen 2018 | 1170 | b | 12751 | b | 94592 | b | 1914615 | b |
| Fuentes-Albero 2024 | 5 | 6 | 7 | 2 | 4 | 4 | 7 | 5 |
| Garcia 2023 | 416774 | b | 522186 | b | 8302850 | b | 18403926 | b |
| Hand 2020 | 676 | b | 4009 | b | 4846 | b | 42004 | b |
| Healy 2017 | 5 | 17 | 41 | b | 1 | 17 | 55 | b |
| Herndon 2009 | 4 | 7 | 30 | 2 | 1 | 3 | 24 | 3 |
| Hill 2015 | 909 | 789 | 3355 | b | 1477 | 2812 | 4555 | b |
| Hyman 2012 | 54 | 50 | 232 | 26 | 81 | 54 | 401 | 23 |
| Islam 2024 | 45 | 45 | 73 | 9 | 17 | 17 | 116 | 22 |
| Kerekes 2015 | 16 | 71 | 244 | 12 | 245 | 1501 | 9047 | 219 |
| Kral 2015 | A | 11 | 14 | b | a | 6 | 24 | b |
| Kummer 2016 | 15 | 13 | 37 | 5 | 1 | 0 | 14 | 4 |
| Levy 2019 | 83 | 101 | 454 | 30 | 69 | 103 | 656 | 56 |
| Liu 2016 | 15 | 12 | 122 | 5 | 15 | 10 | 48 | 0 |
| Mari-Bauset 2015 | 8 | 6 | 79 | 12 | 23 | 53 | 399 | 20 |
| Mathew 2022 | 18 | 25 | 107 | 4 | 18 | 43 | 143 | 9 |
| Mayes 2024 | A | 97 | 657 | 233 | a | 93 | 507 | 65 |
| McCoy 2016 | 203 | 155 | 482 | 69 | 5905 | 6198 | 27431 | 2345 |
| McCoy 2020 | 267 | 190 | 599 | 88 | 3896 | 4395 | 20976 | 1901 |
| Mehra 2024 | A | 383 | 527 | b | a | 8098 | 19324 | b |
| Mendive 2022 | A | 11 | 20 | b | a | 9 | 20 | b |
| Mills 2007 | 20 | 14 | 37 | b | 9 | 4 | 46 | b |
| MMetwally 2024 | 49 | 31 | 200 | 5 | 4 | 8 | 211 | 1 |
| Molina-Lopez 2021 | 8 | 10 | 22 | 9 | 8 | 17 | 65 | 3 |
| Must 2023-CO | 336 | b | 1042 | c | 273 | b | 1105 | c |
| Must 2023-FP | 35 | b | 151 | b | 134 | b | 796 | b |
| Phillips 2014 | 30 | 19 | 40 | 4 | 1066 | 1482 | 5308 | 285 |
| Raspini 2021 | 4 | 15 | 42 | 4 | 1 | 10 | 58 | 13 |
| Riccio 2020 | 6 | 15 | 24 | 2 | 0 | 4 | 34 | 6 |
| Rimmer 2010 | 39 | 29 | 91 | b | 1686 | 1946 | 9341 | b |
| Rouphael 2024 | 30 | 17 | 37 | 2 | 8 | 9 | 56 | 13 |
| Schott 2022 | 15344 | b | 140273 | b | 35994 | b | 430857 | b |
| Sedgewick 2020 | 104 | 86 | 126 | 18 | 61 | 71 | 189 | 9 |
| Shea 2024 | 2005 | b | 11581 | b | 32351 | b | 406206 | b |
| Shedlock 2016 | 4004 | b | 44758 | b | 11402 | b | 232408 | b |
| Trinh 2023 | A | 39 | 51 | 3 | a | 4 | 89 | 0 |
| Tyler 2011 | 29 | 25 | 24 | 5 | 36 | 50 | 51 | 5 |
| Wei 2018 | 3 | b | 80 | 0 | 2 | b | 81 | 0 |
| Weir 2021 | 316 | 308 | 459 | 73 | 241 | 300 | 608 | 35 |
| Zhu 2020 | 67 | b | 671 | b | 30 | b | 272 | b |
| Zimmer 2012 | A | b | 20 | 2 | a | b | 21 | 1 |
| Zurita 2020 | 1 | 3 | 19 | 2 | 0 | 2 | 32 | 0 |

Table S12 Outcomes: number and prevalence of individuals with obesity in the group with and without ASD

| **Author year** | **N with ASD&Obesity** | **N with ASD (No Obesity)** | **N with Obesity (no ASD)** | **N without ASD or Obesity** | **Prevalence (%)**  **Obesity in ASD** | **Prevalence (%)**  **Obesity in controls** |
| --- | --- | --- | --- | --- | --- | --- |
| Ahlberg 2022 | 1945 | 35190 | 59866 | 3044695 | 5.24 | 1.93 |
| Al-Kindi 2016 | 3 | 160 | 2 | 210 | 1.84 | 0.94 |
| Alkhalidy 2021 | 11 | 41 | 5 | 46 | 21.15 | 9.8 |
| Ames 2022 | 509 | 998 | 4073 | 10997 | 33.78 | 27.03 |
| Amin 2022 | 28 | 32 | 28 | 92 | 46.67 | 23.33 |
| Arija 2023 | 22 | 95 | 47 | 286 | 18.8 | 14.11 |
| Barnhill 2017 | 8 | 78 | 4 | 52 | 9.3 | 7.14 |
| Bener 2014 | 5 | 249 | 9 | 245 | 1.97 | 3.54 |
| Błazewicz 2020 | 84 | 68 | 23 | 112 | 55.26 | 17.04 |
| Broder-Fingert 2014 | 709 | 2267 | 233 | 3463 | 23.82 | 6.3 |
| Buro 2022 | 194 | 674 | 3242 | 21993 | 22.35 | 12.85 |
| Castro 2016 | 18 | 31 | 9 | 40 | 36.73 | 18.37 |
| Chen 2016 | 166 | 5956 | 273 | 24215 | 2.71 | 1.11 |
| Coppola 2024 | 24 | 76 | 8 | 92 | 24 | 8 |
| Corbett 2021 | 38 | 98 | 16 | 84 | 27.94 | 16 |
| Criado 2018 | 59 | 217 | 65 | 479 | 21.38 | 11.95 |
| Curtin 2010 | 138 | 316 | 19935 | 64534 | 30.4 | 23.6 |
| Davignon 2018 | 1037 | 3086 | 3164 | 17451 | 25.15 | 15.35 |
| Eliasziw 2021 | 10 | 90 | 1280 | 8370 | 10 | 13.26 |
| Esposito 2019 | 6 | 45 | 3 | 47 | 11.76 | 6 |
| Esteban-Figuerola 2021 | 18 | 61 | 49 | 303 | 22.78 | 13.92 |
| FlygareWallen 2018 | 1170 | 12751 | 94592 | 1914615 | 8.4 | 4.71 |
| Fuentes-Albero 2024 | 5 | 15 | 4 | 16 | 25 | 20 |
| Garcia 2023 | 416774 | 522186 | 8302850 | 18403926 | 44.39 | 31.09 |
| Hand 2020 | 676 | 4009 | 4846 | 42004 | 14.43 | 10.34 |
| Healy 2017 | 5 | 58 | 1 | 72 | 7.94 | 1.37 |
| Herndon 2009 | 4 | 39 | 1 | 30 | 9.3 | 3.23 |
| Hill 2015 | 909 | 4144 | 1477 | 7367 | 17.99 | 16.7 |
| Hyman 2012 | 54 | 308 | 81 | 478 | 14.92 | 14.49 |
| Islam 2024 | 45 | 127 | 17 | 155 | 26.16 | 9.88 |
| Kerekes 2015 | 16 | 327 | 245 | 10767 | 4.66 | 2.22 |
| Kummer 2016 | 15 | 54 | 1 | 18 | 21.7 | 5.32 |
| Levy 2019 | 83 | 585 | 69 | 815 | 12.43 | 7.81 |
| Liu 2016 | 15 | 139 | 15 | 58 | 9.74 | 20.55 |
| Mari-Bauset 2015 | 8 | 97 | 23 | 472 | 7.62 | 4.65 |
| Mathew 2022 | 18 | 136 | 18 | 195 | 11.69 | 8.45 |
| McCoy 2016 | 203 | 706 | 5905 | 35974 | 22.33 | 14.1 |
| McCoy 2020 | 267 | 877 | 3896 | 27272 | 23.3 | 12.5 |
| Mills 2007 | 20 | 51 | 9 | 50 | 28.17 | 15.25 |
| MMetwally 2024 | 49 | 236 | 4 | 220 | 17.19 | 1.79 |
| Molina-Lopez 2021 | 8 | 41 | 8 | 85 | 16.33 | 8.6 |
| Must 2023-CO | 336 | 1042 | 273 | 1105 | 24.38 | 19.81 |
| Must 2023-FP | 35 | 151 | 134 | 796 | 18.82 | 14.41 |
| Phillips 2014 | 30 | 63 | 1066 | 7075 | 32.26 | 13.09 |
| Raspini 2021 | 4 | 61 | 1 | 81 | 6.15 | 1.22 |
| Riccio 2020 | 6 | 41 | 0 | 44 | 12.77 | 0 |
| Rimmer 2010 | 39 | 120 | 1686 | 11287 | 24.53 | 13 |
| Rouphael 2024 | 30 | 56 | 8 | 78 | 34.9 | 9.3 |
| Schott 2022 | 15344 | 140273 | 35994 | 430857 | 9.86 | 7.71 |
| Sedgewick 2020 | 104 | 230 | 61 | 269 | 31.14 | 18.48 |
| Shea 2024 | 2005 | 11581 | 32351 | 406206 | 14.76 | 7.38 |
| Shedlock 2016 | 4004 | 44758 | 11402 | 232408 | 8.21 | 4.68 |
| Tyler 2011 | 29 | 54 | 36 | 106 | 34.94 | 25.35 |
| Wei 2018 | 3 | 80 | 2 | 81 | 3.61 | 2.41 |
| Weir 2021 | 316 | 840 | 241 | 943 | 27.34 | 20.35 |
| Zhu 2020 | 67 | 671 | 30 | 272 | 9.08 | 9.93 |
| Zurita 2020 | 1 | 24 | 0 | 34 | 4 | 0 |

Table S13 Outcomes: number and prevalence of individuals with overweight in the group with and without ASD

Overweight refers to those with overweight or obesity (i.e. those above the 85 percentile)

| **Author year** | **N with ASD&Overweight** | **N with ASD (No Overweight)** | **N with Overweight (no ASD)** | **N without ASD or Overweight** | **Prevalence (%)**  **Overweight in ASD** | **Prevalence (%)**  **Overweight in controls** |
| --- | --- | --- | --- | --- | --- | --- |
| Al-Kindi 2016 | 25 | 138 | 19 | 193 | 15.34 | 8.96 |
| Alkhalidy 2021 | 14 | 38 | 13 | 38 | 26.92 | 25.49 |
| Amin 2022 | 35 | 25 | 42 | 78 | 58.33 | 35 |
| Arija 2023 | 51 | 66 | 128 | 205 | 43.59 | 38.44 |
| Bandini 2013 | 14 | 39 | 13 | 45 | 26.42 | 22.41 |
| Barnhill 2017 | 17 | 69 | 14 | 42 | 19.77 | 25 |
| Bener 2014 | 27 | 227 | 50 | 204 | 10.63 | 19.69 |
| Błazewicz 2020 | 102 | 50 | 53 | 82 | 67.11 | 39.26 |
| Broder-Fingert 2014 | 1116 | 1860 | 636 | 3060 | 37.5 | 17.21 |
| Castro 2016 | 23 | 26 | 21 | 28 | 46.94 | 42.86 |
| Coppola 2024 | 35 | 65 | 19 | 81 | 35 | 19 |
| Corbett 2021 | 57 | 79 | 28 | 72 | 41.91 | 28 |
| Criado 2018 | 117 | 159 | 142 | 402 | 42.39 | 26.1 |
| Davignon 2018 | 1821 | 2302 | 6646 | 13969 | 44.17 | 32.24 |
| Eliasziw 2021 | 30 | 70 | 2820 | 6830 | 30 | 29.22 |
| Esposito 2019 | 11 | 40 | 11 | 39 | 21.57 | 22 |
| Esteban-Figuerola 2021 | 36 | 43 | 138 | 214 | 45.57 | 39.2 |
| Fuentes-Albero 2024 | 11 | 9 | 8 | 12 | 55 | 40 |
| Healy 2017 | 22 | 41 | 18 | 55 | 34.92 | 24.66 |
| Herndon 2009 | 11 | 32 | 4 | 27 | 25.58 | 12.9 |
| Hill 2015 | 1698 | 3355 | 4289 | 4555 | 33.6 | 48.5 |
| Hyman 2012 | 104 | 258 | 135 | 424 | 28.73 | 24.15 |
| Islam 2024 | 90 | 82 | 34 | 138 | 52.33 | 19.77 |
| Kerekes 2015 | 87 | 256 | 1746 | 9266 | 25.36 | 15.86 |
| Kral 2015 | 11 | 14 | 6 | 24 | 44 | 20 |
| Kummer 2016 | 28 | 41 | 1 | 18 | 40 | 5.32 |
| Levy 2019 | 184 | 484 | 172 | 712 | 27.54 | 19.46 |
| Liu 2016 | 27 | 127 | 25 | 48 | 17.53 | 34.25 |
| Mari-Bauset 2015 | 14 | 91 | 76 | 419 | 13.33 | 15.35 |
| Mathew 2022 | 43 | 111 | 61 | 152 | 27.92 | 28.64 |
| Mayes 2024 | 97 | 890 | 93 | 572 | 9.83 | 13.98 |
| McCoy 2016 | 358 | 551 | 12103 | 29776 | 39.38 | 28.9 |
| McCoy 2020 | 456 | 688 | 8291 | 22877 | 39.9 | 26.6 |
| Mehra 2024 | 383 | 527 | 8098 | 19324 | 42.09 | 29.53 |
| Mendive 2022 | 11 | 20 | 9 | 20 | 35.48 | 31.03 |
| Mills 2007 | 34 | 37 | 13 | 46 | 47.89 | 22.03 |
| MMetwally 2024 | 80 | 205 | 12 | 212 | 28.07 | 5.36 |
| Molina-Lopez 2021 | 18 | 31 | 25 | 68 | 36.73 | 26.88 |
| Phillips 2014 | 49 | 44 | 2548 | 5593 | 52.69 | 31.3 |
| Raspini 2021 | 19 | 46 | 11 | 71 | 29.23 | 13.41 |
| Riccio 2020 | 21 | 26 | 4 | 40 | 44.68 | 9.09 |
| Rimmer 2010 | 68 | 91 | 3632 | 9341 | 42.77 | 28 |
| Rouphael 2024 | 47 | 39 | 17 | 69 | 54.7 | 19.8 |
| Sedgewick 2020 | 190 | 144 | 132 | 198 | 56.89 | 40 |
| Trinh 2023 | 39 | 54 | 4 | 89 | 41.94 | 4.3 |
| Tyler 2011 | 54 | 29 | 86 | 56 | 65.06 | 60.56 |
| Weir 2021 | 624 | 532 | 541 | 643 | 53.98 | 45.69 |
| Zurita 2020 | 4 | 21 | 2 | 32 | 16 | 5.88 |

Table S14 Outcomes: number and prevalence of individuals with underweight in the group with and without ASD

| **Author year** | **N with ASD&Underweight** | **N with ASD (No underweight)** | **N with underweight (no ASD)** | **N without ASD or underweight** | **Prevalence (%) underweight in ASD** | **Prevalence (%) underweight in controls** |
| --- | --- | --- | --- | --- | --- | --- |
| Al-Kindi 2016 | 47 | 116 | 67 | 145 | 28.83 | 31.6 |
| Alkhalidy 2021 | 5 | 47 | 1 | 50 | 9.62 | 1.96 |
| Amin 2022 | 1 | 59 | 24 | 96 | 1.67 | 20 |
| Bandini 2013 | 1 | 52 | 0 | 58 | 1.89 | 0 |
| Barnhill 2017 | 4 | 82 | 3 | 53 | 4.65 | 5.36 |
| Błazewicz 2020 | 26 | 126 | 13 | 122 | 17.11 | 9.63 |
| Castro 2016 | 11 | 38 | 3 | 46 | 22.45 | 6.12 |
| Coppola 2024 | 0 | 100 | 4 | 96 | 0 | 4 |
| Corbett 2021 | 6 | 130 | 3 | 97 | 4.41 | 3 |
| Esposito 2019 | 9 | 42 | 4 | 46 | 17.65 | 8 |
| Esteban-Figuerola 2021 | 8 | 71 | 48 | 304 | 10.13 | 13.64 |
| Fuentes-Albero 2024 | 2 | 18 | 5 | 15 | 10 | 25 |
| Herndon 2009 | 2 | 41 | 3 | 28 | 4.65 | 9.68 |
| Hyman 2012 | 26 | 336 | 23 | 536 | 7.18 | 4.11 |
| Islam 2024 | 9 | 163 | 22 | 150 | 5.23 | 12.79 |
| Kerekes 2015 | 12 | 331 | 219 | 10793 | 3.5 | 1.99 |
| Kummer 2016 | 5 | 64 | 4 | 15 | 6.7 | 21 |
| Levy 2019 | 30 | 638 | 56 | 828 | 4.49 | 6.33 |
| Liu 2016 | 5 | 149 | 0 | 73 | 3.25 | 0 |
| Mari-Bauset 2015 | 12 | 93 | 20 | 475 | 11.43 | 4.04 |
| Mathew 2022 | 4 | 150 | 9 | 204 | 2.6 | 4.23 |
| Mayes 2024 | 233 | 754 | 65 | 600 | 23.61 | 9.77 |
| McCoy 2016 | 69 | 840 | 2345 | 39534 | 7.59 | 5.6 |
| McCoy 2020 | 88 | 1056 | 1901 | 29267 | 7.7 | 6.1 |
| MMetwally 2024 | 5 | 280 | 1 | 223 | 1.75 | 0.45 |
| Molina-Lopez 2021 | 9 | 40 | 3 | 90 | 18.37 | 3.23 |
| Phillips 2014 | 4 | 89 | 285 | 7856 | 4.3 | 3.5 |
| Raspini 2021 | 4 | 61 | 13 | 69 | 6.15 | 15.85 |
| Riccio 2020 | 2 | 45 | 6 | 38 | 4.26 | 13.64 |
| Rouphael 2024 | 2 | 84 | 13 | 73 | 2.3 | 15.1 |
| Sedgewick 2020 | 18 | 316 | 9 | 321 | 5.39 | 2.73 |
| Trinh 2023 | 3 | 90 | 0 | 93 | 3.23 | 0 |
| Tyler 2011 | 5 | 78 | 5 | 137 | 6.02 | 3.52 |
| Wei 2018 | 0 | 83 | 0 | 83 | 0 | 0 |
| Weir 2021 | 73 | 1083 | 35 | 1149 | 6.31 | 2.96 |
| Zimmer 2012 | 2 | 20 | 1 | 21 | 9.09 | 4.55 |
| Zurita 2020 | 2 | 23 | 0 | 34 | 8 | 0 |

Figure S1 Risk of obesity in ASD, funnel plot

Figure S2 Risk of obesity in ASD, leave-one-out forest plot

Figure S3 Risk of obesity in ASD, community-based studies only, forest plot

Figure S4 Risk of obesity in ASD, hospital-based studies only, forest plot

Figure S5 Risk of obesity in ASD, quality diagnosis of ASD studies only, forest plot

Figure S6 Risk of obesity in ASD, quality definition of weight studies only, forest plot

Figure S7 Risk of obesity in ASD, eliminating studies with 0 cells only, forest plot

Figure S8 Risk of obesity in ASD, limiting analysis to studies with at least 5 participants per cell, forest plot

Figure S9 Risk of obesity in ASD, Meta-regression with percentage of males in overall sample

Figure S10 Risk of obesity in ASD, Meta-regression with percentage of males in ASD sample

Figure S11 Risk of obesity in ASD, Meta-regression with mean age

Figure S12 Risk of overweight in ASD, funnel plot

Figure S13 Risk of overweight in ASD, leave-one-out forest plot

Figure S14 Risk of overweight in ASD, community-based studies only, forest plot

Figure S15 Risk of overweight in ASD, hospital-based studies only, forest plot

Figure S16 Risk of overweight in ASD, quality diagnosis of ASD studies only, forest plot

Figure S17 Risk of overweight in ASD, quality definition of weight studies only, forest plot

Figure S18 Risk of overweight in ASD, eliminating studies with 0 cells only, forest plot

Figure S19 Risk of overweight in ASD, limiting analysis to studies with at least 5 participants per cell, forest plot

Figure S20 Risk of overweight in ASD, Meta-regression with percentage of males in overall sample

Figure S21 Risk of overweight in ASD, Meta-regression with percentage of males in ASD sample

Figure S22 Risk of overweight in ASD, Meta-regression with mean age

Figure S23 Risk of underweight in ASD, funnel plot

Figure S24 Risk of underweight in ASD, leave-one-out forest plot

Figure S25 Risk of underweight in ASD, community-based studies only, forest plot

Figure S26 Risk of underweight in ASD, hospital-based studies only, forest plot

Figure S27 Risk of underweight in ASD, quality diagnosis of ASD studies only, forest plot

Figure S28 Risk of underweight in ASD, quality definition of weight studies only, forest plot

Figure S29 Risk of underweight in ASD, eliminating studies with 0 cells only, forest plot

Figure S30 Risk of underweight in ASD, limiting analysis to studies with at least 5 participants per cell, forest plot

Figure S31 Risk of underweight in ASD, Meta-regression with percentage of males in overall sample

Figure S32 Risk of underweight in ASD, Meta-regression with percentage of males in ASD sample

Figure S33 Risk of underweight in ASD, Meta-regression with mean age
